# Supplementary material for: The ribosome‐associated chaperone Zuo1 controls translation upon TORC1 inhibition
Source: EMBO J. 2023 Nov 20;42(24):e113240. doi: 10.15252/embj.2022113240 (PMC10711665; doi:10.15252/embj.2022113240)
Supplement: Supplementary file 1 — Expanded View Figures PDF [file EMBJ-42-e113240-s007.pdf]

Expanded View Figures

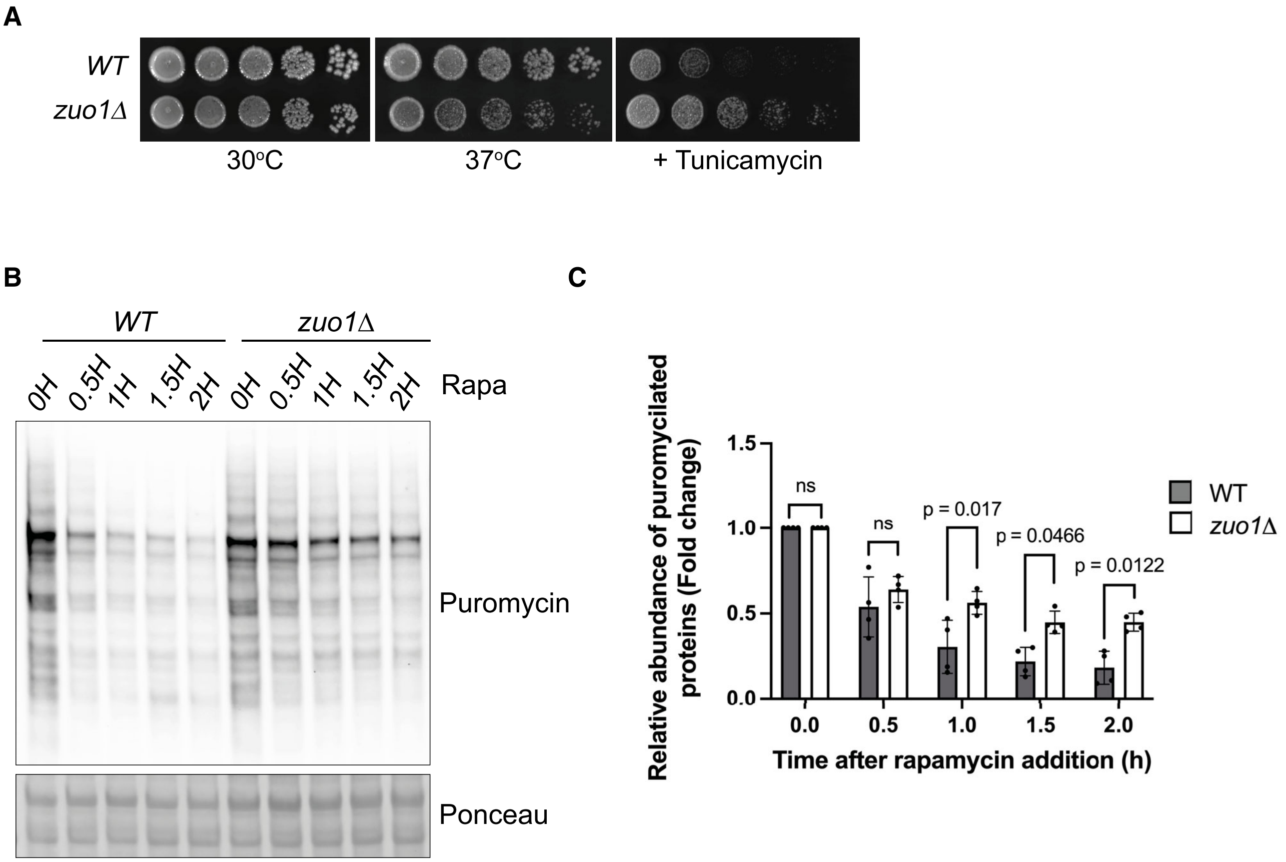

Figure EV1. Sensitivity of Zuo1 to other stresses.

A Fivefold serial dilutions of the indicated strains grown on YEPD plates with or without 0.75 μg/ml tunicamycin for 4 days at 30 or 37°C, where indicated.

B Immunoblot analysis of lysates from WT and *zuo1Δ* cells pulse-chased with 0.5 mM puromycin for 15 min before being treated with 35 μg/ml cycloheximide for the indicated time. Ponceau staining served as the loading control.

C Graph shows densitometry analysis (mean ± s.d.) of the relative abundance of puromycylated proteins (normalised to Pgk1 levels) from (B) relative to the 0H time point. Statistical significance was assessed using two-way ANOVA *t*-test ($n = 4$ independent biological replicates). n.s. (not significant).

Source data are available online for this figure.

© 2023 The Authors

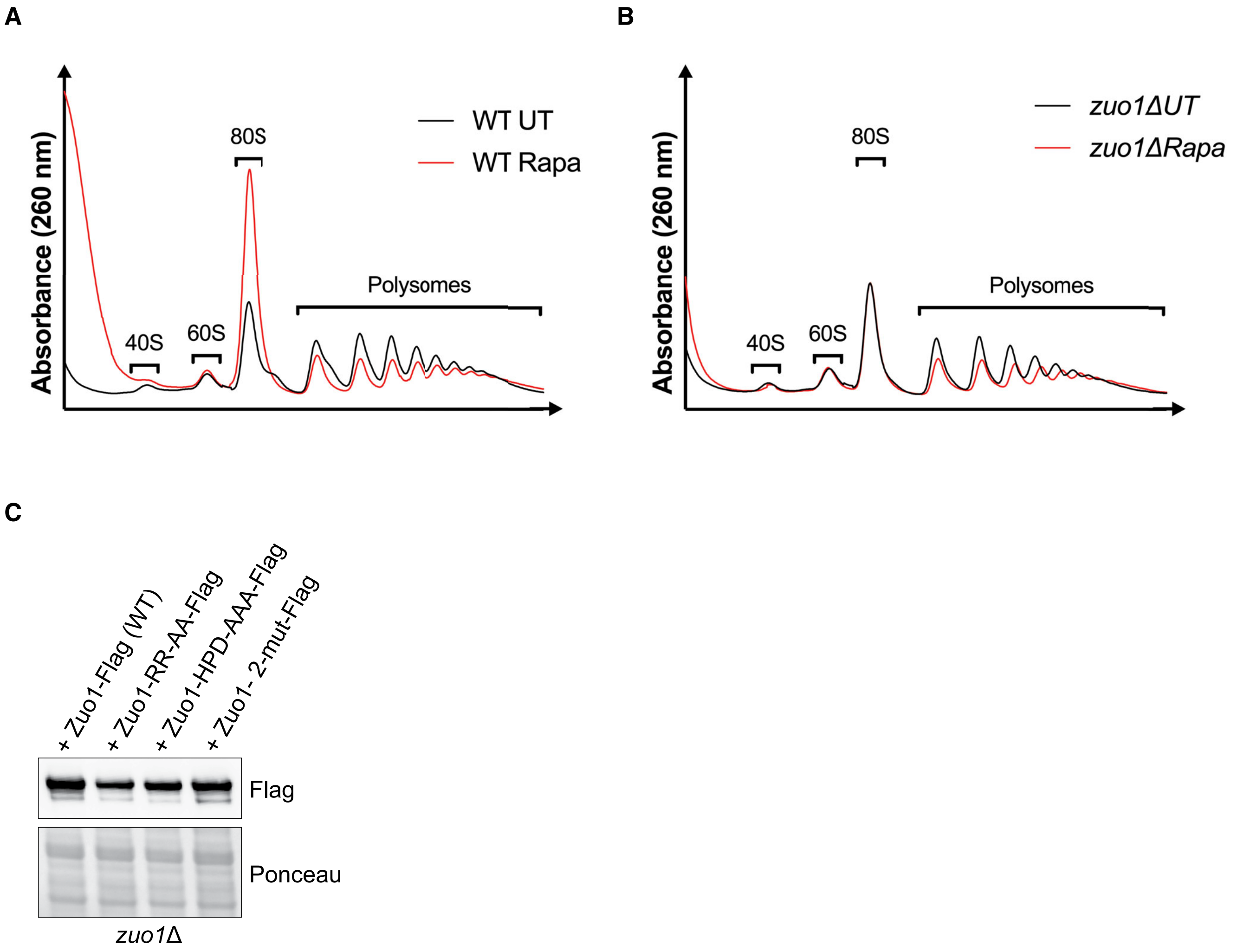

Figure EV2. Polysome profiling analysis of WT and *zuo1*Δ cells.

A, B Polysome profiles of WT (A) and *zuo1*Δ (B) strains either treated with 200 nM rapamycin for 2 h or left untreated. Total extracts were separated on 10–50% sucrose gradients and subsequently the A_{260} was monitored during fractionation.

C Immunoblot analysis of lysates from *zuo1Δ* cells expressing Flag-tagged WT and mutants Zuo1 on a plasmid. Ponceau staining served as the loading control.

Source data are available online for this figure.

Figure EV3. Gene ontology analysis of Zuo1 interactors.

A Protein–protein interaction network of Zuo1 interactors having at least 50% peptide coverage.

B Gene ontology analysis of Zuo1 interactors having at least 50% peptide coverage.

C Table showing Zuo1 interactors having their total protein level significantly increased or decreased by 1.5-fold based on multiplexed quantitative proteomics (https://www.science.org/doi/10.1126/scisignal.2002548). Zuo1 interactors more and less bound to Zuo1-GFP upon rapamycin treatment are shown in red and blue respectively.

D Immunoblot analysis of lysates from WT cells containing Ura7-5xFLAG at the endogenous locus treated with 200 nM rapamycin for 2 h or left untreated. Ponceau staining served as the loading control.

Source data are available online for this figure.

© 2023 The Authors

A

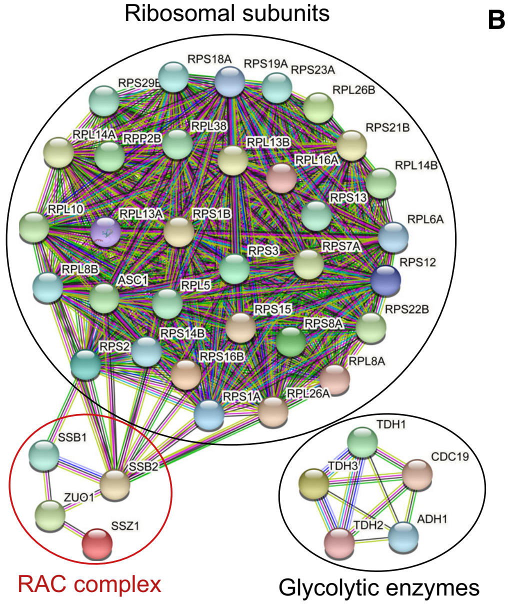

B

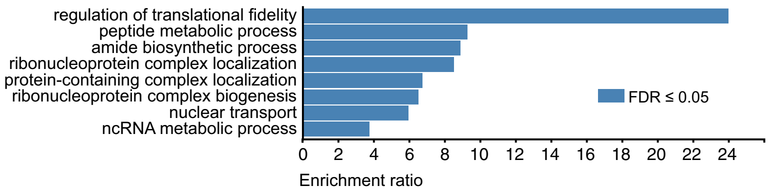

C

Hits induced by rapamycin	Hits unafected by rapamycin	Hits reduced by rapamycin	Hits not detected
CPS1	LSP1	TIF4631	ARO10
CPA2	HYR1	URA7	BAT2
TDH1	TRP3		ADH5
AIM41	VPS4		SNA2
HSP26	FMP52		
PET10	GTT1		
SDH1	DAK1		
SRY1	ADE8		
HFD1	PIL1		
SDH2	TUM1		
PEP4	TDH2		
ALT1	ACO1		
TSL1	APA1		
GGA1	LYS20		
PNC1	MAE1		
DLD3			
HSP78			
DUR1,2			
DCS1			
PYC1			
MPM1			
LYS21			

D

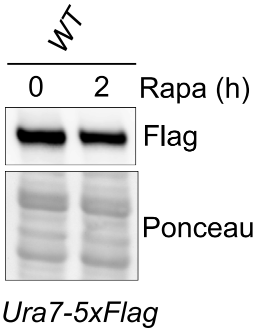

Figure EV3.

 © 2023 The Authors

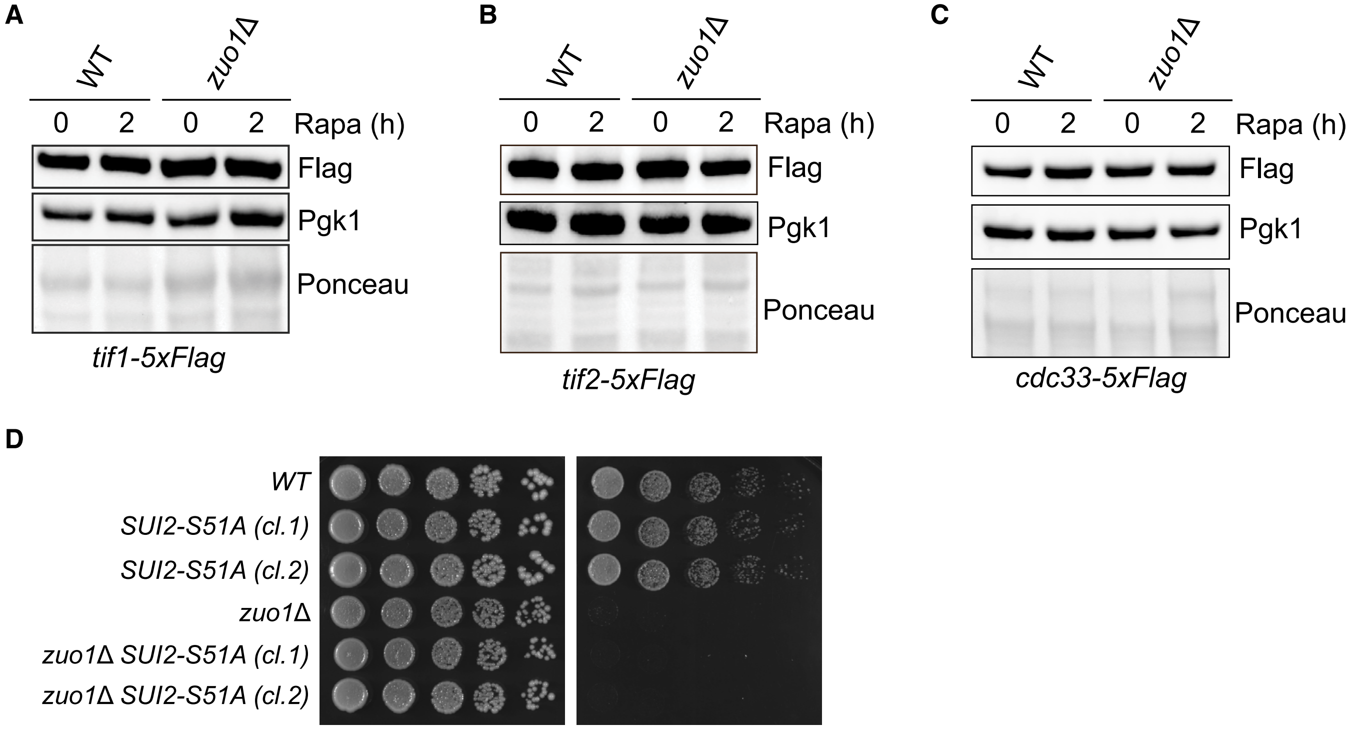

Figure EV4. Abundance of eIF4F subunits upon TORC1 inhibition.

A–C Immunoblot analysis of lysates from WT and *zuo1*Δ cells containing TIF1-5xFLAG (A), TIF2-5xFLAG (B), or CDC33-5xFLAG (C) at the endogenous locus treated with 200 nM rapamycin for 2 h or left untreated. Ponceau and Pgk1 staining served as the loading control.

D Fivefold serial dilutions of the indicated strains grown on YEPD plates with or without 20 ng/ml rapamycin for 4 days at 30°C.

Source data are available online for this figure.

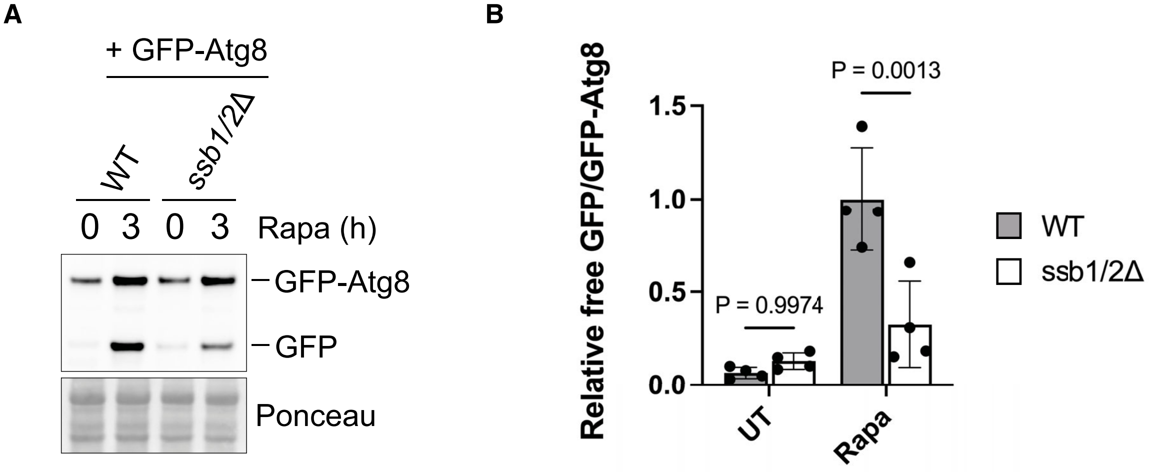

Figure EV5. Autophagy analysis in *ssb1/2Δ* cells.

A Immunoblot analysis of lysates from WT and *ssb1/2Δ* cells expressing GFP-Atg8 on a plasmid treated with 200 nM rapamycin for 3 h or left untreated. Ponceau staining served as the loading control.

B Graph shows densitometry analysis (mean ± s.d.) of the relative abundance of GFP-Atg8 and free GFP from (A), relative to the 0 h time point. Statistical significance was assessed using two-way ANOVA *t*-test (n = 4 independent biological replicates). n.s. (not significant).

Source data are available online for this figure.

 © 2023 The Authors
