# Supplementary material for: The ribosome‐associated chaperone Zuo1 controls translation upon TORC1 inhibition
Source: EMBO J. 2023 Nov 20;42(24):e113240. doi: 10.15252/embj.2022113240 (PMC10711665; doi:10.15252/embj.2022113240)
Supplement: Supplementary file 5 — PDF+ [file EMBJ-42-e113240-s013.pdf]

Article

SOURCE DATA | TRANSPARENT PROCESS | OPEN ACCESS

THE EMBO JOURNAL

The ribosome-associated chaperone Zuo1 controls translation upon TORC1 inhibition

Ailsa Black, Thomas D Williams, Flavie Soubigou, Ifeoluwapo M Joshua, Houjiang Zhou, Frederic Lamoliatte & Adrien Rousseau*

Abstract

Protein requirements of eukaryotic cells are ensured by proteostasis, which is mediated by tight control of TORC1 activity. Upon TORC1 inhibition, protein degradation is increased and protein synthesis is reduced through inhibition of translation initiation to maintain cell viability. Here, we show that the ribosome-associated complex (RAC)/Ssb chaperone system, composed of the HSP70 chaperone Ssb and its HSP40 co-chaperone Zuo1, is required to maintain proteostasis and cell viability under TORC1 inhibition in *Saccharomyces cerevisiae*. In the absence of Zuo1, translation does not decrease in response to the loss of TORC1 activity. A functional interaction between Zuo1 and Ssb is required for proper translational control and proteostasis maintenance upon TORC1 inhibition. Furthermore, we have shown that the rapid degradation of eIF4G following TORC1 inhibition is mediated by autophagy and is prevented in zuo1Δ cells, contributing to decreased survival in these conditions. We found that autophagy is defective in zuo1Δ cells, which impedes eIF4G degradation upon TORC1 inhibition. Our findings identify an essential role for RAC/Ssb in regulating translation in response to changes in TORC1 signalling.

Keywords proteostasis; ribosome-associated chaperones; stress; TORC1; translation

Subject Categories Autophagy & Cell Death; RNA Biology; Translation & Protein Quality

DOI 10.15252/embj.2022113240 | Received 8 December 2022 | Revised 23 October 2023 | Accepted 26 October 2023 | Published online 20 November 2023

The EMBO Journal (2023) 42: e113240

Introduction

Cells must regularly adapt their proteome in order to respond to changing environmental conditions. The coordination of cellular processes to maintain a stable and functional proteome is known as protein homeostasis (proteostasis), the dysregulation of which has been linked to various disease states, including neurodegenerative disorders (Labbadia & Morimoto, 2015; Klaips *et al*, 2017).

A key regulator of the proteostasis network is the target of rapamycin complex 1 (TORC1), which adapts the equilibrium between catabolic and anabolic processes in response to environmental signals (Ben-Sahra & Manning, 2017). In nutrient-rich conditions, TORC1 promotes transcription, translation and ribosome biogenesis (Cardenas *et al*, 1999; Powers & Walter, 1999; Lippman & Broach, 2009), while restricting proteasomal and autophagic activity (Kamada *et al*, 2010; Rousseau & Bertolotti, 2016; Williams *et al*, 2022), to ensure cellular protein requirements for growth are met. Additionally, a network of chaperones ensure that proteins reach their native conformation and prevent the accumulation of protein aggregates (Hartl *et al*, 2011). Nascent polypeptide chains are particularly vulnerable to misfolding and aggregation. Therefore, eukaryotes utilise a specialised chaperone system composed of the ribosome-associated complex (RAC) and the Ssb class of Hsp70 chaperones in yeast (cytosolic Hsp70 in mammalian cells) (Gautschi *et al*, 2002; Jaiswal *et al*, 2011; Zhang *et al*, 2017). The RAC is anchored near the ribosome exit tunnel and consists of the Hsp40 chaperone, Zuo1 (ZRF1/MPP11 in mammals), and the atypical Hsp70, Ssz1 (HSP70L1 in mammals). The RAC recruits Ssb1 and Ssb2 (collectively known as Ssb) and stimulates their ATPase activity, facilitating their interaction with nascent chains as they emerge from the exit tunnel, protecting them from misfolding and aggregation (Willmund *et al*, 2013; Döring *et al*, 2017).

In response to stress, the proteome must be rapidly rewired to execute stress survival mechanisms (Causton *et al*, 2001; Kolkman *et al*, 2006; Shenton *et al*, 2006). The inhibition of TORC1 under various stresses including nutrient deprivation, assists with this essential rewiring and stress response. Protein degradation is increased through upregulation of proteasome activity and autophagy induction, increasing the pool of available amino acids which can be redirected towards the synthesis of stress response proteins (Kamada *et al*, 2010; Rousseau & Bertolotti, 2018). Complementing this is a global reduction in translation, which is often accompanied by translational reprogramming, resulting in only selective translation of proteins required for stress survival (Ashe *et al*, 2000; Preiss *et al*, 2003; Melamed *et al*, 2008; Simpson & Ashe, 2012; Williams *et al*, 2022). In addition to that, most cytosolic chaperones are transcriptionally upregulated in response to a variety of stresses to enable protein folding to occur under more difficult conditions, with several having a well-documented role in responding to proteotoxic stress (Gillies *et al*, 2012). Notably, this is not the case for Zuo1 which is coregulated with the translational machinery and has not yet been

MRC Protein Phosphorylation and Ubiquitylation Unit, School of Life Sciences, University of Dundee, Dundee, UK
*Corresponding author. Tel: +44 1382 384109; E-mail: arousseau@dundee.ac.uk

© 2023 The Authors. Published under the terms of the CC BY 4.0 license

implicated in the refolding of stress-denatured proteins. Interestingly, it has been found that *zuo1Δ* cells are sensitive to the selective inhibitor of TORC1, rapamycin (Gillies *et al*, 2012). However, the role of Zuo1 in responding to TORC1 inhibition is unknown.

Ternary complex formation and eIF4F complex formation are two main aspects of translation regulation upon TORC1 inhibition. Ternary complex formation is probably the most well-characterised mode of regulation. Upon cell stress, the α subunit of eIF2 referred to as SUI2 is phosphorylated on a conserved serine residue (Serine 51), which increases its affinity for its guanine nucleotide exchange factor (GEF) eIF2B (Krishnamoorthy *et al*, 2001). This inhibits eIF2B GEF activity, preventing the exchange of GDP for GTP on eIF2. The GTP-bound form of eIF2 has a much greater affinity for Met-tRNAi than eIF2-GDP and so ternary complex regeneration is severely restricted by phosphorylation of eIF2α (Kapp & Lorsch, 2004). In yeast, Gcn2 is responsible for eIF2α phosphorylation. Loss of TORC1 activity or binding by uncharged tRNA results in dephosphorylation and activation of Gcn2 which phosphorylates eIF2α at S51. This induces a global reduction of protein synthesis. The second regulatory mechanism affects mRNA recognition by the eIF4F complex. In yeast TORC1 positively regulates the protein levels of both eIF4G proteins (eIF4G1 and 2 also known as TIF4631 and TIF4632 respectively), which are the least abundant member of the eIF4F complex. Upon TORC1 inactivation, eIF4G is rapidly degraded by autophagy (Berset *et al*, 1998; Powers & Walter, 1999). Complex assembly, and consequently mRNA recognition and translation initiation, is thus impeded by loss of eIF4G. The regulation of translation factor stability by TORC1 allows for their rapid depletion in adverse conditions, which helps modulate translation in response to changes in TORC1 activity.

Here we investigate the importance of Zuo1 for maintaining proteostasis following TORC1 inhibition in *S. cerevisiae*. We demonstrate a role of Zuo1 in attenuating the rate of translation following TORC1 inhibition. This is dependent on its role as a ribosome-associated co-chaperone for Ssb. We further identify Zuo1 interaction partners and found that one candidate, the translation initiation factor eIF4G2, is misregulated following TORC1 inactivation in *zuo1Δ* cells. This contributes to the loss of cell growth upon TORC1 inhibition. We further found that autophagy is defective in *zuo1Δ* cells, which impairs eIF4G degradation upon TORC1 inhibition. Together, these results define that the RAC-Ssb complex is fine-tuning the rate of translation upon TORC1 inhibition, so that proteostasis, and thereby cell viability, is maintained.

Results

Proteostasis is impaired upon TORC1 inhibition in *zuo1Δ* cells

It has previously been shown that loss of Zuo1 sensitises cells to the TORC1 inhibitor rapamycin (Gillies *et al*, 2012). In agreement with this, we find that functionality of the RAC is important for surviving rapamycin challenge, as loss of either complex member results in increased sensitivity to rapamycin, with *zuo1Δ* cells displaying a more acute sensitivity than *ssz1Δ* cells (Fig 1A and B). This appears to be specific for TORC1 inhibition by rapamycin as *zuo1Δ* cells displayed only very mild sensitivity to heat shock and were more resistant to tunicamycin-mediated endoplasmic reticulum (ER)-stress than WT cells (Fig EV1). This may be due to TORC1 being still active at 37°C and tunicamycin mainly relying on the Ire1 branch for stress survival.

As TORC1 is a central coordinator of the protein homeostasis network, we analysed if proteostasis was disrupted by loss of Zuo1, by assessing the load of polyubiquitinated conjugates. A decrease in polyubiquitinated conjugates was seen in WT cells following TORC1 inhibition (Fig 1C), resulting from increased protein degradation and translation inhibition (Rousseau & Bertolotti, 2018; Advani & Ivanov, 2019; Williams & Rousseau, 2022). However, this decrease in polyubiquitinated proteins following rapamycin treatment was not observed in *zuo1*Δ cells. This was despite TORC1 being inhibited to a similar extent as in WT cells, with TORC1 activity assessed by phosphorylation of its target Rps6 (Fig 1C), as previously described (Yerlikaya *et al*, 2016). To examine the clearance of misfolded cytosolic proteins, we utilised the misfolded mutant of carboxypeptidase Y (CPY*) fused to GFP and lacking its endoplasmic reticulum targeting signal (Δss-CPY*GFP) as a reporter (Medicherla *et al*, 2004). This reporter is continually degraded by the proteasome but becomes stabilised in cells with a defect in the ubiquitin proteasome system. In agreement with the impaired clearance of ubiquitinated proteins observed, clearance of Δss-CPY*GFP, is delayed in *zuo1Δ* cells, following rapamycin treatment (Fig 1D and E). To monitor overall protein degradation, we performed a pulse-chase experiment using puromycin. The pulse-chase of puromycin generates a pool of puromycylated proteins that can be subsequently assessed for degradation over time. Similar to Δss-CPY*GFP, the clearance of puromycylated proteins was compromised in *zuo1Δ* cells upon rapamycin treatment (Fig EV1B and C). Together, this indicates that *zuo1Δ* cells are unable to properly adapt their proteostasis network in response to TORC1 inhibition by rapamycin.

Proteasomal degradation is unaffected in *zuo1Δ* cells

Impaired clearance of polyubiquitinated proteins can be a sign of defective protein degradation by the proteasome. Following TORC1 inhibition, cells boost their degradative capacity by increasing proteasome assembly and activity (Rousseau & Bertolotti, 2016; Waite *et al*, 2022; Williams *et al*, 2023). To assess proteasome function in *zuo1Δ* cells, we performed an in-gel peptidase assay. The advantage of this method is that it can resolve the activity of all proteasomal species separated on a native gel after adding an internally quenched fluorogenic peptide. Fluorescence from the peptide is only observed after cleavage by the proteasome, and hence fluorescence intensity mirrors proteasome activity. The increase in proteasome activity following rapamycin treatment is not compromised in *zuo1Δ* cells, and this was true for both singly- and doubly capped proteasomes (RP-CP and RP_2-CP respectively) (Fig 2A). In agreement, when ribosomal translation is inhibited by cycloheximide the degradation of the proteasome reporter substrate ΔssCPY*GFP is comparable in WT and *zuo1Δ* cells (Fig 2B and C). Thus, the loss of proteostasis in *zuo1Δ* cells is not due to a defect in proteasomal degradation.

Translation is not reduced in *zuo1Δ* mutants following TORC1 inhibition

A possible explanation as to why Δss-CPY*GFP levels remain higher in rapamycin-treated *zuo1*Δ cells, compared to WT cells, despite the

 © 2023 The Authors

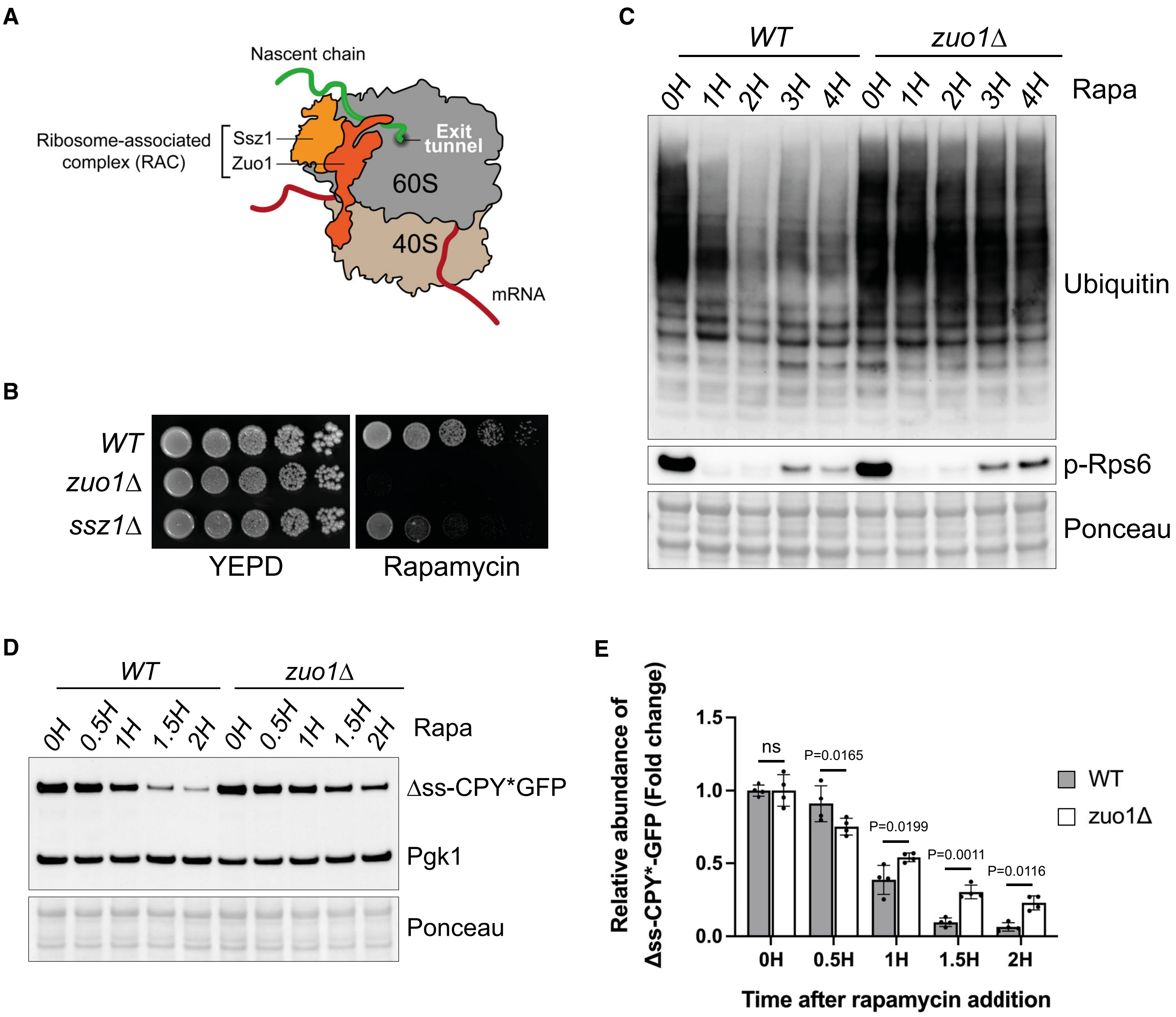

Figure 1. Zuo1 controls protein homeostasis upon TORC1 inhibition.

A Cartoon depicting the ribosome-associated complex. Zuo1 interacts with both 40S and 60S ribosomal subunits and Ssz1 remains associated with the ribosome through its interaction with Zuo1. The RAC sits near the polypeptide exit tunnel where it can interact with nascent chains.

B Fivefold serial dilutions of the indicated strains grown on YEPD plates with or without 20 ng/ml rapamycin for 4 days at 30°C.

C Immunoblot analysis of lysates from WT and *zuo1Δ* cells treated with 200 nM rapamycin for the indicated time. Ponceau staining served as the loading control.

D WT and *zuo1Δ* cells expressing Δss-CPY*GFP from a plasmid were treated with 200 nM rapamycin for the indicated time. The resulting lysates were analysed by immunoblotting. Ponceau and Pgk1 staining served as the loading control.

E Graph shows densitometry analysis (mean ± s.d.) of the relative abundance of Δss-CPY*GFP (normalised to Pgk1 levels) from (D) relative to the 0H time point. Statistical significance was assessed using two-way ANOVA *t*-test ($n = 4$ independent biological replicates). n.s. (not significant).

Source data are available online for this figure.

clearance of this reporter being unimpeded, is that translation may be continuing despite TORC1 inhibition. Failure to reduce translation following rapamycin treatment would lead to excess production of unwanted proteins, overwhelming the proteostasis network. To test this hypothesis, we employed the SUnSET assay in which the aminoacyl tRNA analogue, puromycin, is incorporated into nascent chains, reflecting the rate of translation *in vivo* (Schmidt *et al*, 2009). Following rapamycin treatment, translation decreases in WT cells, correlating with TORC1 inhibition (Fig 3A). Strikingly, rapamycin treatment fails to decrease translation in *zuo1Δ* cells following rapamycin-induced TORC1 inhibition. Correspondingly, a decrease in polysome levels is seen in WT cells following rapamycin treatment (Fig EV2A). While *zuo1Δ* cells have lower polysome levels in basal conditions (Albanèse *et al*, 2010),

© 2023 The Authors

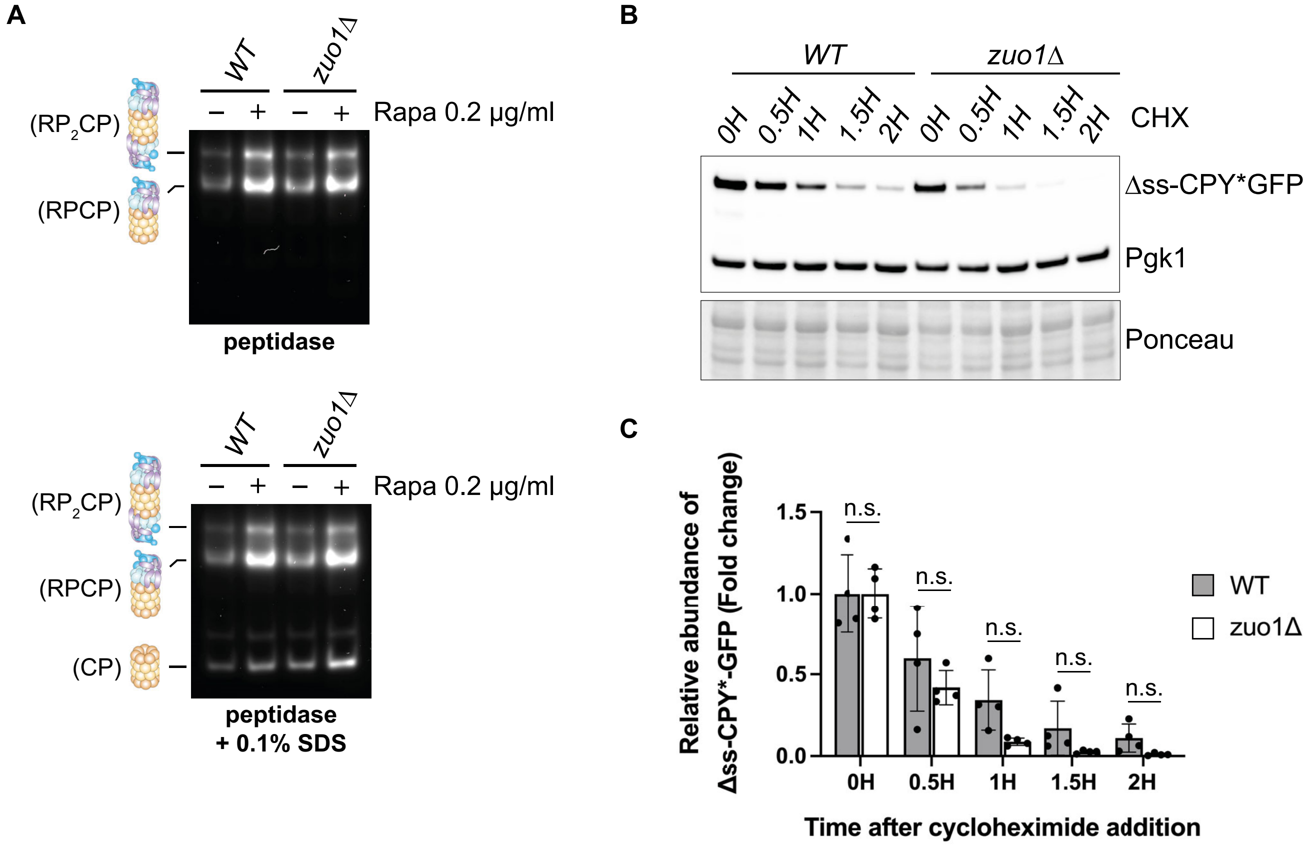

Figure 2. Proteasome homeostasis is not impaired in *zuo1Δ* cells.

A Yeast extracts from cells treated with 200 nM rapamycin for 3 h or left untreated were separated by native-PAGE (3.8–5% gradient) and peptidase activity detected using the fluorogenic substrate Suc-LLVY-AMC in the presence or absence of 0.1% SDS. RP_2CP, double-capped proteasome; RPCP, single-capped proteasome; and CP, core particle complexes are indicated.

B Immunoblot analysis of lysates from WT and *zuo1Δ* cells expressing Δss-CPY*GFP from a plasmid treated with 35 µg/ml cycloheximide for the indicated time. Ponceau and Pgk1 staining served as the loading control.

C Graph shows densitometry analysis (mean ± s.d.) of the relative abundance of Δss-CPY*GFP (normalised to Pgk1 levels) from (D) relative to the 0H time point. Statistical significance was assessed using two-way ANOVA *t*-test (n = 4 independent biological replicates). n.s. (not significant).

Source data are available online for this figure.

this does not seem to affect the *in vivo* translation rate (Fig 3A). This is consistent with previous studies reporting that overall translation rate is not impaired in RAC-deleted cells, despite polysome levels being lower (Albanèse *et al*, 2010; Willmund *et al*, 2013; Hanebuth *et al*, 2016). Furthermore, the decrease in polysome levels observed in WT cells upon TORC1 inhibition, is not present in *zuo1Δ* cells (Fig EV2B), suggesting that a failure to properly regulate translation may comprise the proteostasis defect detected. Confirming this,

Figure 3. Translation is not reduced in *zuo1Δ* cells upon TORC1 inhibition.

A Immunoblots of lysates from WT and zuo1Δ cells grown in the presence of 0.5 mM puromycin, with or without 200 nM rapamycin for 2 h. Ponceau staining served as the loading control.

B Immunoblot analysis of lysates from WT and *zuo1Δ* cells grown in the presence 0.5 mM puromycin and treated with 200 nM rapamycin and/or 35 µg/ml cycloheximide as indicated, for 2 h. Ponceau staining served as the loading control.

C Immunoblot analysis of lysates from WT and *zuo1Δ* cells treated with 200 nM rapamycin and/or 35 µg/ml cycloheximide as indicated, for 2 h. Ponceau staining served as the loading control.

D Immunoblot analysis of lysates from WT and *zuo1Δ* cells expressing Δss-CPY*GFP from a plasmid treated with 200 nM rapamycin and/or 35 µg/ml cycloheximide for the indicated time. Ponceau and Pgk1 staining served as the loading control.

E Graph shows densitometry analysis (mean ± s.d.) of the relative abundance of Δss-CPY*GFP (normalised to Pgk1 levels) from WT and *zuo1Δ* cells treated with 200 nM rapamycin and/or 35 µg/ml cycloheximide as indicated, for 2 h. Statistical significance was assessed using two-way ANOVA *t*-test (n = 4 independent biological replicates). n.s. (not significant).

Source data are available online for this figure.

 © 2023 The Authors

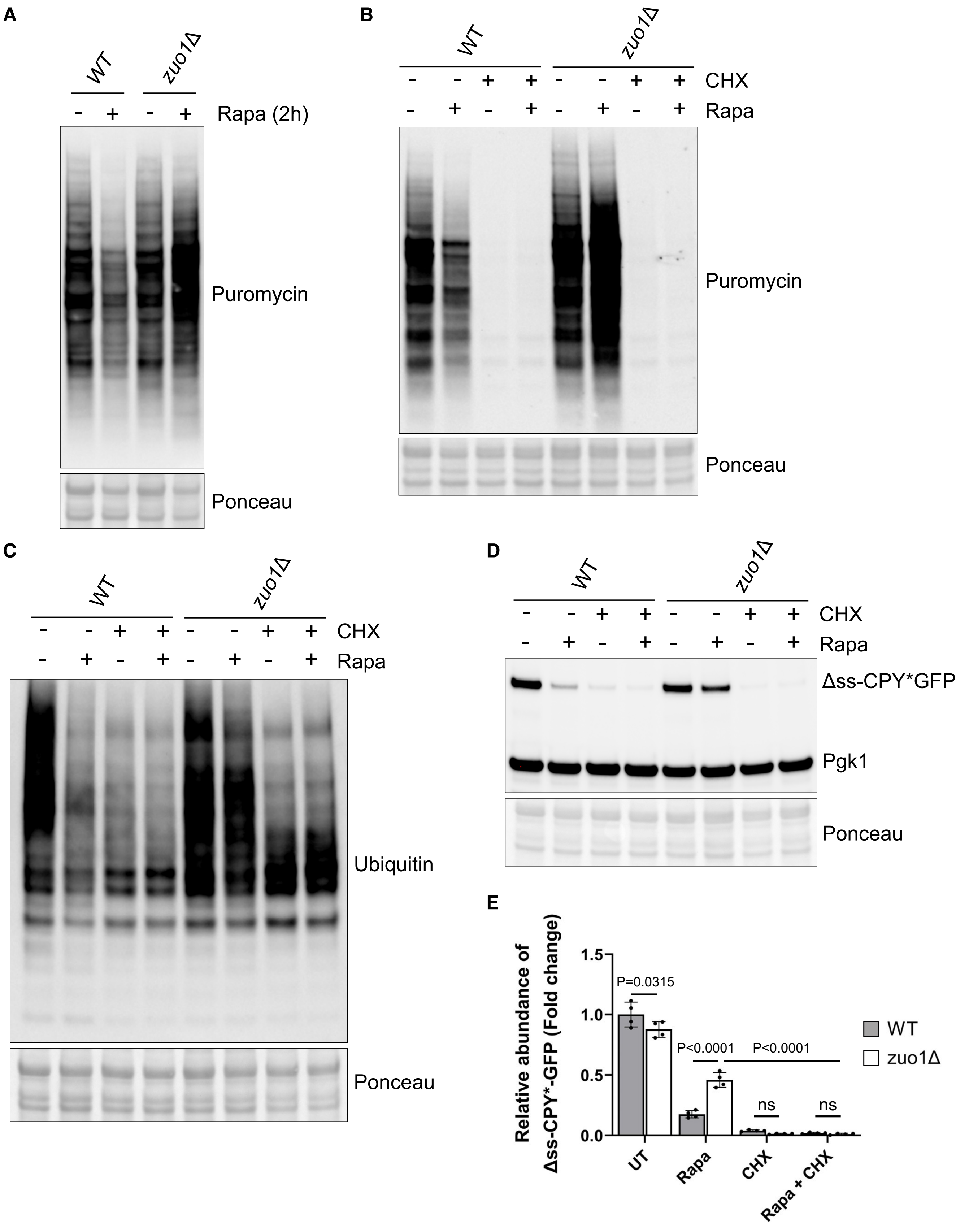
A
WT
zuo1Δ
- + - + Rapa (2h)
Puromycin
Ponceau
B
WT
zuo1Δ
- - + + - - + + CHX
- + - + - + - + Rapa
Puromycin
Ponceau
C
WT
zuo1Δ
- - + + - - + + CHX
- + - + - + - + Rapa
Ubiquitin
Ponceau
D
WT
zuo1Δ
- - + + - - + + CHX
- + - + - + - + Rapa
Δss-CPY*GFP
Pgk1
Ponceau
E
Relative abundance of Δss-CPY*-GFP (Fold change)
1.5
1.0
0.5
0.0
P=0.0315
P<0.0001
P<0.0001
ns
ns
UT
Rapa
CHX
Rapa + CHX
WT
zuo1Δ

Figure 3.

cycloheximide treatment inhibited translation in both WT and *zuo1Δ* cells, even in the presence of rapamycin (Fig 3B), and partly rescued the impaired clearance of polyubiquitinated proteins observed in *zuo1*Δ cells following TORC1 inhibition (Fig 3C). Similarly, while clearance of Δss-CPY*GFP is impeded in *zuo1Δ* cells treated with rapamycin alone, cotreatment with cycloheximide restores the clearance of the misfolded proteasome reporter in *zuo1*Δ cells following TORC1 inhibition (Figs 1D and E, and 3D and E). Together, these data demonstrate that the failure to shut down translation in response to TORC1 inhibition likely underpins the protein homeostasis defect in *zuo1Δ* cells.

Zuo1 controls translation upon TORC1 inhibition through Ssb chaperones

Having established the importance of Zuo1 in mediating a translational decrease following TORC1 inhibition, we set out to define how it achieves this. Zuo1, as a key component of the RAC, functions as a cochaperone for Ssb. However, Zuo1 is also reported to have additional functions (Fig 4A). The first is in recruitment of Ltn1, a key E3 ligase of ribosome-associated quality control (Ghosh & Shcherbik, 2020), and the second is in the induction of pleiotropic drug resistance, through the Pdr1 transcription factor (Eisenman & Craig, 2004). Deletion of Ltn1 or Pdr1 does not confer sensitivity to rapamycin (Fig 4B) or result in impaired clearance of polyubiquitinated proteins (Fig 4C), suggesting that these roles of Zuo1 are dispensable for its role in adapting the proteostasis network in response to TORC1 inhibition. Deletion of both homologues of Ssb (ssb1 and ssb2), however, results in acute sensitivity to rapamycin and is accompanied by a defect in clearing polyubiquitinated proteins following TORC1 inhibition, analogous to *zuo1Δ* cells (Fig 4B and C). Cells lacking Ssb also fail to shut down translation in response to TORC1 inhibition (Fig 4D). As in *zuo1Δ* cells, this seems to underpin the proteostasis defect, as restoring translation inhibition with cycloheximide in *ssb1/2Δ* cells improves their clearance of polyubiquitinated proteins upon rapamycin treatment (Fig 4E).

zuo1Δ and *ssb1/2Δ* cells share sensitivity to many of the same stresses and drugs, such as translation inhibitors and cold stress (Hundley *et al*, 2002). For many of these, it has been demonstrated that the sensitivity of *zuo1Δssb1/2Δ* cells is the same as that of *zuo1Δ* cells and is not additive (Hundley *et al*, 2002). Likewise, we have found that the phenotype of *zuo1Δssb1/2Δ* cells resembles that of cells lacking either Zuo1 or Ssb, displaying similarly low levels of growth and impaired polyubiquitin clearance upon rapamycin treatment (Fig 5A and B). Additionally, translation is not shut down in *zuo1Δssb1/2Δ* cells following TORC1 inhibition, but the overall phenotype is not more severe than either *zuo1*Δ cells or *ssb1/2Δ* cells (Fig 5C). This further emphasises that the role of Zuo1 in maintaining proteostasis upon TORC1 inhibition is dependent on its role as a ribosome-associated co-chaperone.

To confirm this result, we utilised two mutations in Zuo1 which impair crucial functions in regulating Ssb: a ribosome-binding mutant in which two key arginine residues (R247/R251) are mutated to alanine, destabilising the interaction of Zuo1 with ribosomes (RR-AA) (Kaschner *et al*, 2015), and a HPD-AAA mutant in which the conserved HPD motif in the J domain of Zuo1 has been mutated, inhibiting its ability to stimulate the ATPase activity of Ssb (Fig 5D) (Huang *et al*, 2005; Gumiero *et al*, 2016). Expression of WT Zuo1 on a plasmid complemented the growth defect of *zuo1Δ* cells in response to rapamycin, whereas expression of either of the RR-AA or HPD-AAA Zuo1 mutants did not (Fig 5E). All mutants were efficiently expressed in *zuo1Δ* cells with only Zuo1-RR-AA having lower level of expression, which could also account for its lower ability to rescue *zuo1Δ* cells (Fig EV2). However, the rapamycin sensitivity of *zuo1Δ* cells re-expressing either of these Zuo1 mutants is not as severe as cells expressing the control plasmid (EV: Empty Vector). Similarly, relative to WT cells, the clearance of ubiquitinated proteins is impaired in *zuo1Δ* cells expressing either the ribosome binding or chaperone mutant of Zuo1, but to a lesser extent than in *zuo1Δ* cells harbouring the control plasmid (Fig 5F). Translation is also not fully shut down following TORC1 inhibition in cells expressing the single Zuo1 mutants; however, the phenotype is not as severe as that displayed by *zuo1Δ* cells (Fig 5G). We next generated a double mutant (RR-AA/HPD-AAA; zuo1-2-mut) lacking both ribosome binding and a functional J domain, as Zuo1 with single mutation is known to keep residual function (Hundley *et al*, 2002). Zuo1-2mut showed similar level of expression as WT Zuo1 (Fig EV2). Cells expressing Zuo1-2mut displayed greater sensitivity to rapamycin than those expressing the single mutants, more akin to deletion of the protein (Fig 5E). Accordingly, expression of Zuo1-2mut was not able to rescue the defective clearance of polyubiquitinated proteins and shutdown of translation observed in *zuo1Δ* cells upon TORC1 inhibition (Fig 5F and G). This confirms the importance of Zuo1 function in regulating Ssb chaperones for maintaining proteostasis in response to TORC1 inhibition.

Identification of Zuo1 partners upon TORC1 inhibition

We have determined that of the known roles of Zuo1, only its role in activating Ssb at the ribosome appears to be necessary for

Figure 4. Ssb reduces translation upon TORC1 inhibition.

A Cartoon illustrating the known roles of Zuo1. Zuo1 is the cochaperone of Ssb, recruiting it to the ribosome and stimulating its ATPase activity. The recruitment of a key ubiquitin ligase in ribosome-associated quality control, Ltn1, to the ribosome is facilitated by Zuo1. The transcription factor Pdr1 can be activated by Zuo1 when it is not ribosome associated and the C-terminal 4-helix bundle is unfolded.

B Cells of the indicated strains were spotted in five-fold serial dilutions onto YEPD plate ± 20 ng/ml rapamycin and grown for 4 days at 30°C.

C Immunoblot analysis of lysates from cells of the indicated genotype treated with 200 nM rapamycin for 4 h or left untreated. Ponceau staining served as the loading control.

D Immunoblot analysis of lysates from WT, *zuo1*Δ, and *ssb1/2*Δ cells grown in the presence of 0.5 mM puromycin, with or without 200 nM rapamycin for 2 h. Ponceau staining served as the loading control.

E Immunoblot analysis of lysates from cells of the indicated genotype treated with 200 nM rapamycin and/or 35 μg/ml cycloheximide as indicated, for 2 h. Ponceau staining served as the loading control.

Source data are available online for this figure.

 © 2023 The Authors

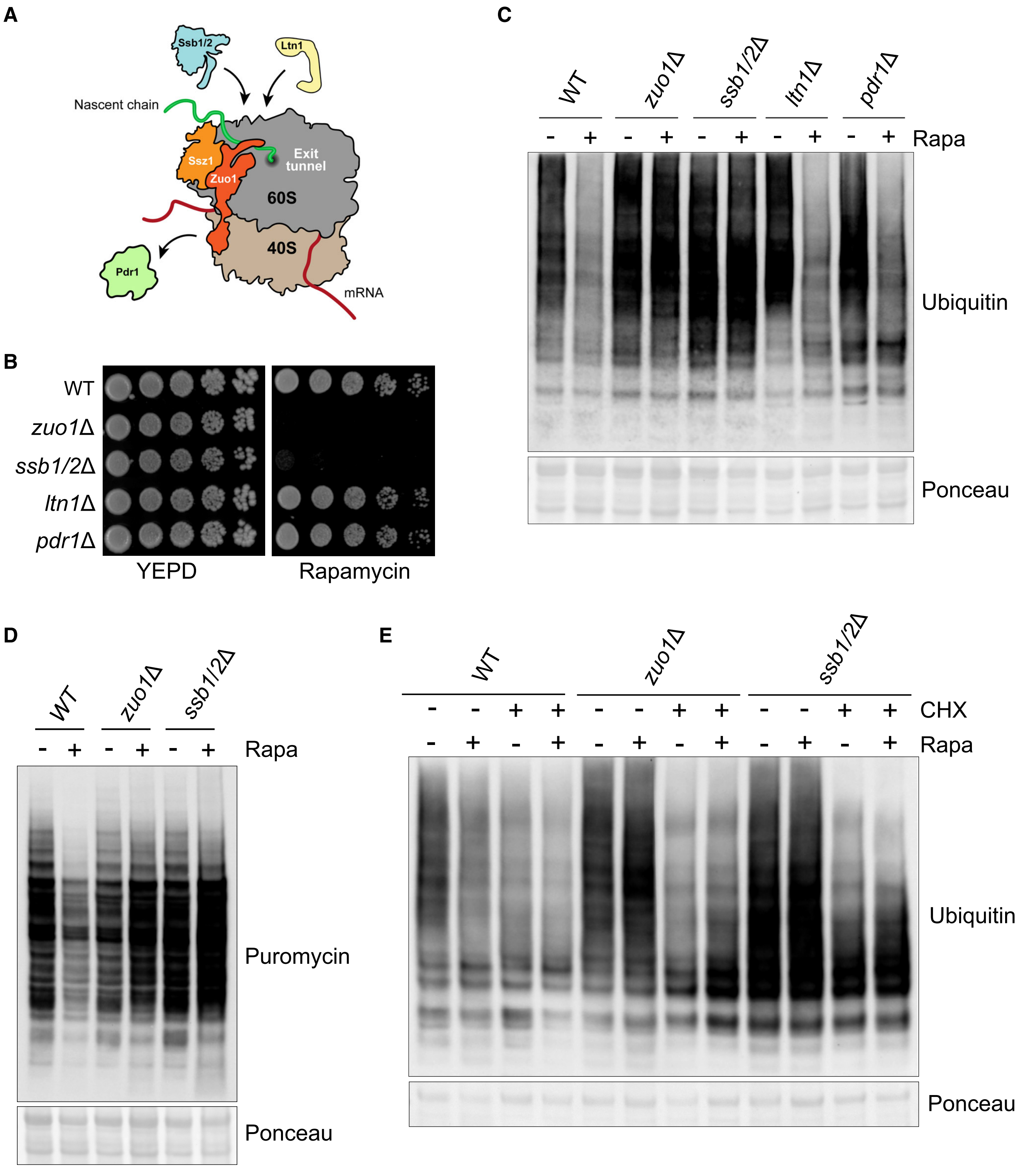

Figure 4.

regulating proteostasis in cells challenged with rapamycin. However, it is unclear if additional Zuo1 partners are important for this regulation. We, therefore, immunoprecipitated endogenously tagged Zuo1 from cells grown in the presence or absence of rapamycin (Fig 6A). We then performed tandem mass tag (TMT)-based quantitative proteomics to identify novel interactors and analyse changes in its interactions (Fig 6B). All the members of the RAC/Ssb chaperone system and many ribosomal proteins were found to be interacting with Zuo1 in our dataset (all proteins with at least 50% peptide coverage; Fig EV3A). A subset of glycolytic enzymes was

© 2023 The Authors

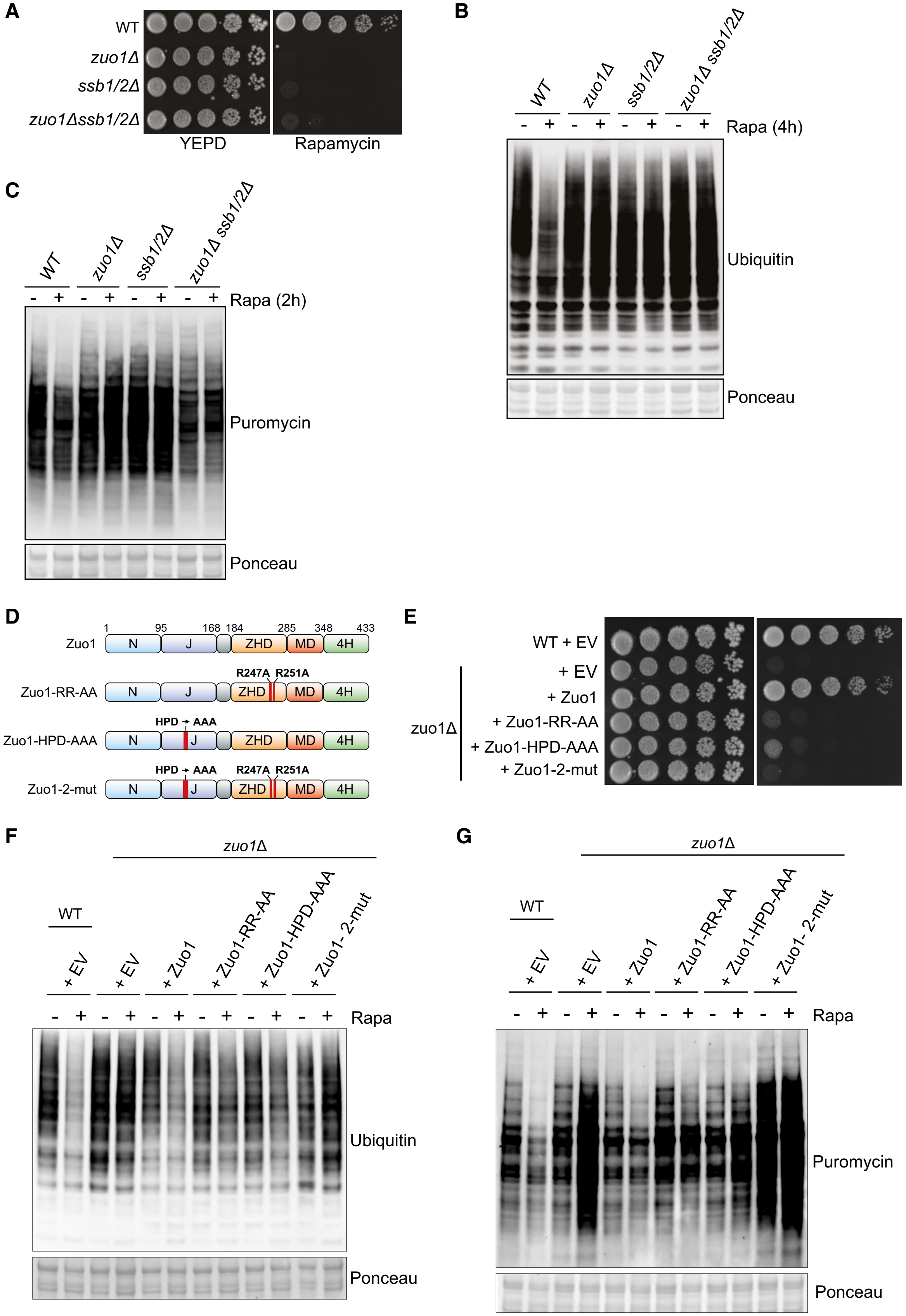

Figure 5.

© 2023 The Authors

◀ **Figure 5. Zuo1-mediated Ssb regulation controls translation upon TORC1 inhibition.**

A Cells of the indicated strains were spotted in five-fold serial dilutions onto YEPD plate ± 20 ng/ml rapamycin and grown for 4 days at 30°C.

B Immunoblot analysis of lysates from cells of the indicated genotype treated with 200 nM rapamycin for 4 h or left untreated. Ponceau staining served as the loading control.

C Immunoblot analysis of lysates from the indicated strains grown in the presence of 0.5 mM puromycin, with or without 200 nM rapamycin for 2 h. Ponceau staining served as the loading control.

D Zuo1 mutants used in this study. The domain architecture of Zuo1 is illustrated with mutated residues highlighted in red. N-terminal domain (N), J-domain (J), Zuotin homology domain (ZHD), middle domain (MD), four-helix bundle (4H).

E WT and *zuo1*Δ cells expressing the indicated plasmids or empty vector (EV) were spotted in five-fold serial dilutions onto YEPD plate ± 20 ng/ml rapamycin and grown for 4 days at 30°C.

F Immunoblot analysis of lysates from WT and *zuo1*Δ cells expressing the indicated plasmids with or without 200 nM rapamycin for 2 h. Ponceau staining served as the loading control.

G Immunoblot analysis of lysates from WT and *zuo1*Δ cells expressing the indicated plasmids or empty vector (EV) were grown in the presence of 0.5 mM puromycin, with or without 200 nM rapamycin for 2 h. Ponceau staining served as the loading control.

Source data are available online for this figure.

also identified to be interacting with Zuo1. These genes are highly expressed and, with one exception, have been shown to be RAC-dependent substrates of Ssb (15), which likely accounts for their identification as Zuo1 interactors. Furthermore, gene ontology analysis revealed a strong enrichment of proteins linked to translation fidelity as well as those involved in ribonucleoprotein biogenesis and localization, processes which Zuo1 is known to be involved in Fig EV3B. This highlights the robustness of this dataset and gives confidence in the novel Zuo1 interactors identified.

We identified 39 and 11 proteins whose interaction with Zuo1 was significantly increased and decreased upon rapamycin treatment respectively. Those which are known to be linked to either translation or chaperone activity have been highlighted in Fig 6B as they are of particular interest for Zuo1's role in proteostasis. An increase in Zuo1 interaction upon rapamycin treatment can also be due to an increase in Zuo1 interactor levels. Therefore, we have listed Zuo1 interactors significantly changing by more than 1.5-fold upon rapamycin treatment using published quantitative proteomic datasets (Fig EV3C) (Dephoure & Gygi, 2012). Hits with level unaffected by rapamycin are more likely to have their interaction with Zuo1 modulated by rapamycin. However, in order to prevent bias, we have included all 43 identified interactors which are not essential. If these interactors are important for mediating the role of Zuo1 in proteostasis, then it would be expected that their loss would confer a similar phenotype to that of *zuo1Δ* cells upon rapamycin treatment. Deletion mutants of the 43 interactors were initially screened for rapamycin sensitivity, with only *trp3*Δ, *vps4*Δ, *pil1*Δ and *tum1*Δ cells displaying increased rapamycin sensitivity relative to WT cells (Fig 6C). Subsequently, a proteostasis defect in response to rapamycin challenge was observed in cells lacking Vps4 but not in *trp3*Δ, *pil1*Δ or *tum1*Δ cells (Fig 6D). However, in *vps4*Δ cells, this defect is not at the level of translation regulation as following TORC1 inhibition translation is still inhibited (Fig 6E). None of the Zuo1 interactors examined seem to be involved in its role in regulating proteostasis following rapamycin treatment. Although the proteostasis defect in *vps4Δ* does not appear to be linked to Zuo1, further study would be valuable to elucidate the defect in the proteostasis network of these cells.

Degradation of eIF4G is impeded in *zuo1Δ* cells

None of the novel interactors identified appear to be involved in mediating Zuo1's role in maintaining proteostasis in response to TORC1 inhibition. However, one interactor stood out as being particularly interesting, eIF4G2 (encoded by TIF4632 in yeast), despite the deletion mutant displaying no sensitivity towards rapamycin (Fig 6C). This could be due to the presence of its paralogue, eIF4G1 (encoded by TIF4631), as they are functionally redundant. eIF4G1/2 are scaffold proteins in the eIF4F complex which is required for recruiting the 40S ribosomal subunit to mRNA during translation initiation (reviewed in Hinnebusch & Lorsch, 2012). Translation initiation is the predominant target of TORC1 translational regulation. TORC1 elicits control over this process by promoting the maintenance of eIF4G levels in nutrient-rich conditions, facilitating

Figure 6. Identification of Zuo1 partners upon TORC1 inhibition. ▶

A Cells expressing endogenously tagged Zuo1-GFP grown in the presence or absence of 200 nM rapamycin for 1.5 h, subjected to immunoprecipitation using an anti-GFP nanobody and analysed by immunoblotting.

B Volcano plot illustrating Zuo1 interactors from Zuo1-GFP immunoprecipitates, differentially enriched in UT and rapamycin-treated conditions. Red, orange and green dots represent proteins with a false discovery rate of < 0.01, < 0.05 and < 0.1 respectively. The dotted lines indicate the threshold for two-fold differential enrichment between UT and rapamycin-treated conditions ($n = 3$ independent biological replicates).

C Growth assays of deletion mutants of non-essential Zuo1 interactors from (B) with greater than two-fold change in enrichment between rapamycin and UT conditions. The indicated strains were grown on YEPD plates with or without 20 ng/ml rapamycin for 4 days. Strains which display increased sensitivity towards rapamycin compared to WT cells are highlighted with an asterisk (*).

D WT cells and rapamycin sensitive deletion mutants from (C) were treated with 200 nM rapamycin for 4 h or left untreated. The resulting lysates were analysed by immunoblotting. Ponceau staining served as the loading control.

E Immunoblots of lysates from WT, *zuo1*Δ and *vps4*Δ cells grown in the presence of 0.5 mM puromycin, with or without 200 nM rapamycin for 2 h. Ponceau staining served as the loading control.

Source data are available online for this figure.

© 2023 The Authors

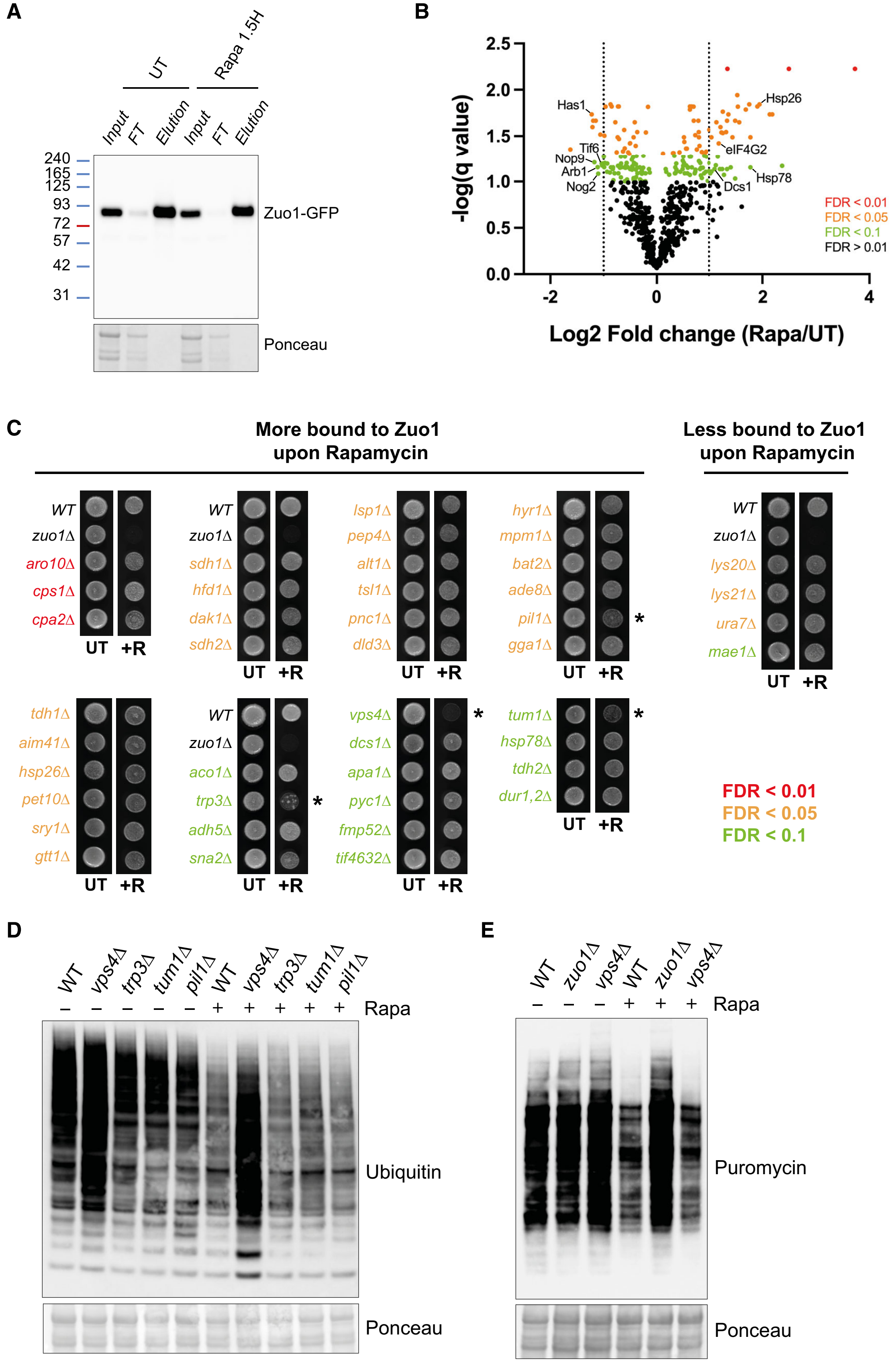

Figure 6.

 © 2023 The Authors

translation initiation. Following TORC1 inhibition, for example, by rapamycin treatment, eIF4G is rapidly degraded to suppress bulk translation (Berset *et al*, 1998). In *zuo1Δ* cells, the dramatic reduction of eIF4G1 and eIF4G2 that is observed in WT cells following rapamycin treatment, is severely impaired (Fig 7A–D). The protein level of other eIF4F complex members, in both basal- and rapamycin-treated conditions, is unaffected by loss of Zuo1, indicating that this misregulation is specific to eIF4G (Fig EV4A–C). We next searched our candidate Zuo1 partners for significant reduction in their total protein levels upon rapamycin treatment (Fig EV3C). We only found one additional protein, Ura7, predicted to have reduced levels upon rapamycin treatment. Tagging endogenous Ura7, we found that its levels was not affected by rapamycin treatment (Fig EV3D). This suggests that the regulation of protein stability by Zuo1 is not a common phenomenon, and it is rather specific.

Interestingly, it has been reported that eIF4G is the least abundant of the eIF4 factors and hence is rate-limiting for eIF4F activity (von der Haar & McCarthy, 2002; Gilbert *et al*, 2007). This suggests that *zuo1Δ* cells may be sustaining translation through the excess pool of eIF4G. To clarify whether the increased pool of eIF4G proteins observed in *zuo1Δ* cells treated with rapamycin is a cause or consequence of the proteostasis defect, the effect of overexpressing eIF4G1 and eIF4G2 in WT cells was examined. Over 24 h, overexpression of either eIF4G paralog did not lead to a notable growth defect in WT cells under basal conditions (Fig 7E). However, overexpression of eIF4G causes a striking reduction of growth in WT cells grown in the presence of rapamycin, indicating that appropriate regulation of eIF4G is required for cells to survive rapamycin challenge. In agreement with that, it has been shown that eIF4G overexpression is preventing the decrease in translation mediated by TORC1 inhibition upon nutrient starvation (Holmes *et al*, 2004). In contrast, the growth of *zuo1Δ* cells challenged with rapamycin is not further impaired by the overexpression of eIF4G (Fig 7F). Altogether, these results suggest that failure to reduce eIF4G protein levels upon rapamycin treatment in *zuo1Δ* cells may contribute to the loss of proteostasis.

An additional TORC1 downstream effector in translational regulation is Sui2 (also known as eIF2α). Following TORC1 inhibition, Sui2 is phosphorylated at S51, which hinders translation initiation (Krishnamoorthy *et al*, 2001; von der Haar & McCarthy, 2002). We next investigated whether Zuo1 translation regulation is mediated by Sui2. We have first introduced SUI2-S51A mutation in WT and *zuo1Δ* cells using CRISPR-Cas9 genome editing to define whether blocking Sui2 phosphorylation is impacting the translation defect of *zuo1Δ* cells. We found that Sui2-S51A mutation was not rescuing translation and growth defects of *zuo1Δ* cells upon rapamycin treatment, while completely abolishing phosphorylation at S51 (Figs 7G and H, and S4D). Moreover, phosphorylation of Sui2 upon rapamycin treatment was unaffected by the absence of Zuo1 (Fig 7H). Together, these results show that, following TORC1 inhibition, *zuo1Δ* cells may be sustaining translation through the remaining pool of eIF4G, independently of Sui2.

In addition to suppressing translation initiation, TORC1 inhibition elicits a large transcriptional response. This is particularly well characterised for the ribosome biogenesis (RiBi) regulon, which consists of genes linked to ribosome biogenesis and translation (Hardwick *et al*, 1999; Powers & Walter, 1999). The mRNA levels of these genes decrease in response to TORC1 inhibition, which, in addition to shut down of bulk translation, contributes to the reduction of their synthesis. eIF4G1 is known to be a constituent of the RiBi regulon, with its gene expression being rapidly downregulated following TORC1 inhibition. Similar regulation of eIF4G2 has not been established. To determine whether the defect in downregulating the protein levels of eIF4G in *zuo1Δ* cells is a result of transcriptional regulation, changes in the mRNA levels following rapamycin treatment were monitored using quantitative real-time PCR. As expected, gene expression of eIF4G1 is substantially downregulated upon rapamycin treatment in WT cells, whereas eIF4G2 mRNA levels appear to be largely unchanged by the treatment (Fig 8A). The transcriptional regulation of eIF4G1 and eIF4G2 does not seem to be impacted by the loss of Zuo1, suggesting that the defect in regulating eIF4G protein levels in *zuo1Δ* cells is occurring post-transcriptionally.

It has been reported that eIF4G proteins are selectively degraded upon TORC1 inhibition in yeast (Kelly & Bedwell, 2015). Proteasomal degradation does not substantially contribute to eIF4G degradation as inhibition of the proteasome using MG-132 does not prevent the degradation of eIF4G upon TORC1 inhibition (Fig 8B). This is consistent with no defect in proteasome activity being identified in *zuo1Δ* cells (Fig 2). In contrast, loss of autophagic degradation by deletion of Atg1, which is essential for autophagy, inhibited the degradation of eIF4G2 upon rapamycin treatment, in

Figure 7. Zuo1 controls eIF4G protein turnover upon TORC1 inhibition.

A Immunoblot analysis of lysates from WT and *zuo1*Δ cells containing eIF4G1-5xFLAG at the endogenous locus treated with 200 nM rapamycin for the indicated time. Ponceau and Pgk1 staining served as the loading control.

B Graph shows densitometry analysis (mean ± s.d.) of the relative abundance of eIF4G1-5xFLAG from (A), relative to the 0 h time point. Statistical significance was assessed using two-way ANOVA *t*-test ($n = 4$ independent biological replicates). n.s. (not significant).

C Immunoblot analysis of lysates from WT and *zuo1*Δ cells containing eIF4G1-5xFLAG (A) or eIF4G2-5xFLAG (C) at the endogenous locus treated with 200 nM rapamycin for the indicated time. Ponceau and Pgk1 staining served as the loading control.

D Graph shows densitometry analysis (mean ± s.d.) of the relative abundance of eIF4G1-5xFLAG and eIF4G2-5xFLAG from (A) and (C), respectively, relative to the 0 h time point. Statistical significance was assessed using two-way ANOVA *t*-test ($n = 4$ independent biological replicates). n.s. (not significant).

E Growth curves of WT cells overexpressing eIF4G1 on a plasmid grown in selective medium in the presence or absence of 200 nM rapamycin. OD_{600} measurements were taken at 30-min intervals for 24 h.

F Growth curves of WT cells overexpressing eIF4G2 on a plasmid grown in selective medium in the presence or absence of 200 nM rapamycin. OD_{600} measurements were taken at 30-min intervals for 24 h.

G Fivefold serial dilutions of the indicated strains grown on YEPD plates with or without 20 ng/ml rapamycin for 4 days at 30°C.

H Immunoblots of lysates from WT and *zuo1*Δ cells ± CRISPR-Cas9-mediated SUI2-S51A mutation grown in the presence of 0.5 mM puromycin, with or without 200 nM rapamycin for 2 h. Ponceau staining served as the loading control.

Source data are available online for this figure.

© 2023 The Authors

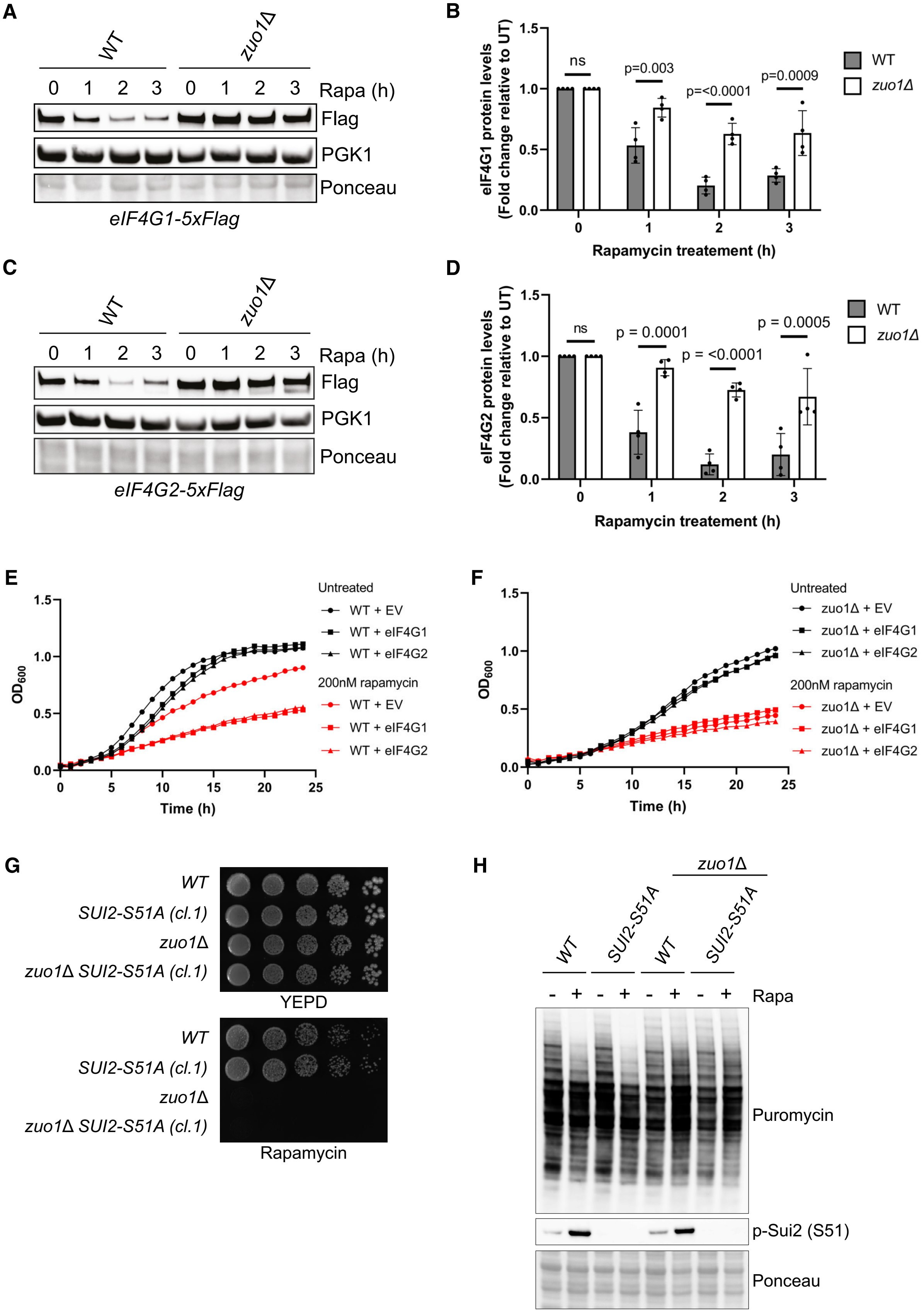

Figure 7.

© 2023 The Authors

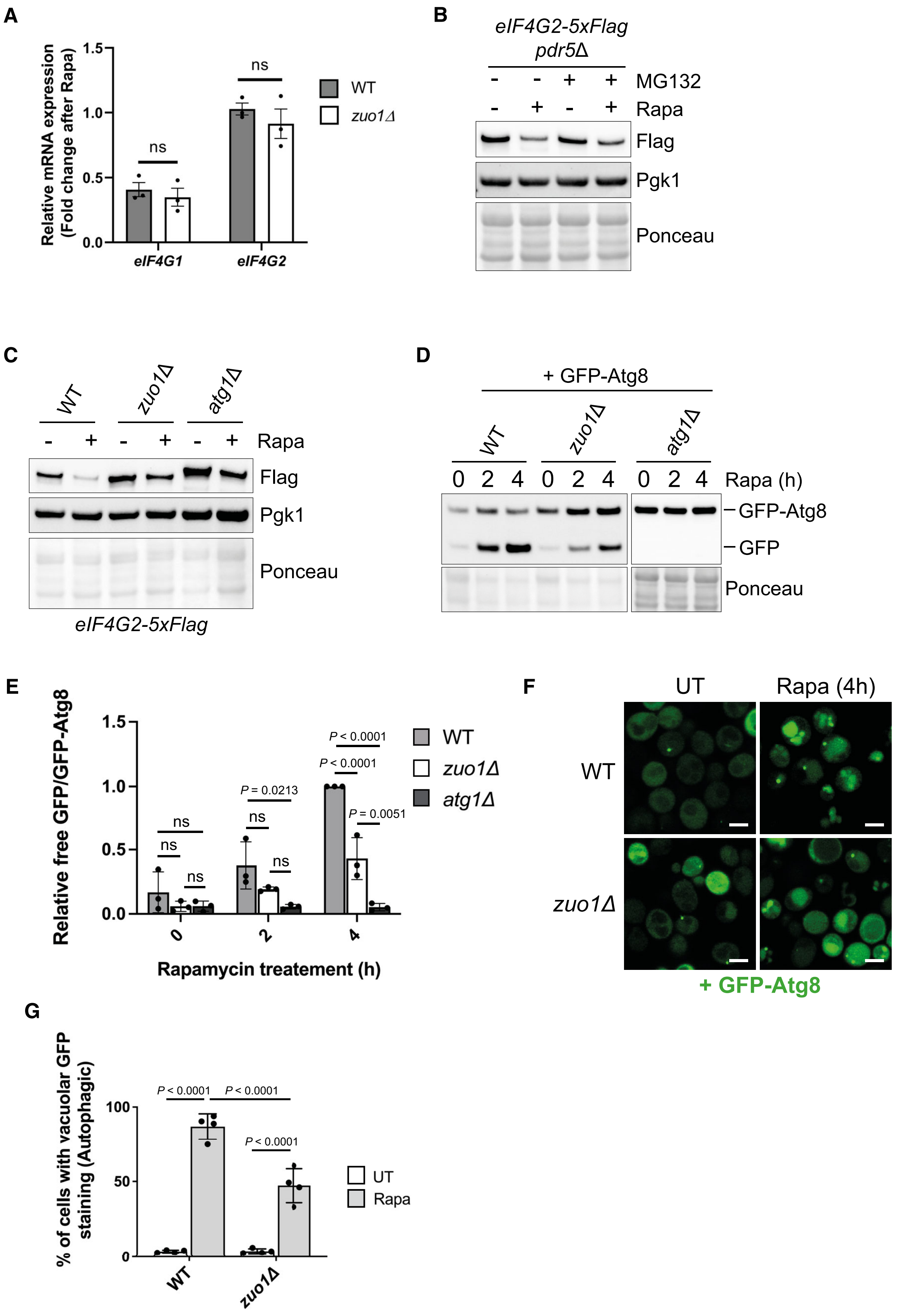
A
Relative mRNA expression (Fold change after Rapa)
ns
ns
WT
zuo1Δ
eIF4G1
eIF4G2
B
eIF4G2-5xFlag pdr5Δ
MG132
Rapa
Flag
Pgk1
Ponceau
C
WT
zuo1Δ
atg1Δ
Rapa
Flag
Pgk1
Ponceau
eIF4G2-5xFlag
D
+ GFP-Atg8
Rapa (h)
GFP-Atg8
GFP
Ponceau
E
Relative free GFP/GFP-Atg8
P < 0.0001
P = 0.0213
P = 0.0051
Rapamycin treatement (h)
F
UT
Rapa (4h)
+ GFP-Atg8
G
% of cells with vacuolar GFP staining (Autophagic)
UT
Rapa

Figure 8.

Figure 8. Zuo1 controls eIF4G degradation through autophagy.

A mRNA levels of eIF4G1 and eIF4G2 from WT and *zuo1Δ* cells treated with 200 nM rapamycin for 2 h or left untreated were analysed by qRT-PCR. The fold change in mRNA levels in rapamycin-treated conditions relative to UT conditions is presented as the mean ± s.e.m. Expression of eIF4G1 and eIF4G2 mRNA was normalised to the housekeeping gene ALG9. Statistical significance was assessed using multiple unpaired *t*-test (n = 3 independent biological replicates). n.s. (not significant).

B Immunoblot analysis of lysates from *pdr5Δ* cells containing eIF4G2-5xFLAG at the endogenous locus treated with 200 nM rapamycin and 50 μM MG-132, where indicated, for 2 h or left untreated. Ponceau and Pgk1 staining served as the loading control.

C Immunoblot analysis of lysates from WT, *zuo1Δ* and *atg1Δ* cells containing eIF4G2-5xFLAG at the endogenous locus treated with 200 nM rapamycin for 2 h or left untreated. Ponceau and Pgk1 staining served as the loading control.

D Immunoblot analysis of lysates from WT, *zuo1Δ* and *atg1Δ* cells expressing GFP-Atg8 on a plasmid treated with 200 nM rapamycin for the indicated time or left untreated. Ponceau staining served as the loading control. Samples were run on the same gels and analysed on the same membrane, but intervening lanes were removed for clarity.

E Graph shows densitometry analysis (mean ± s.d.) of the relative abundance of GFP-Atg8 and free GFP from (D), relative to the 0 h time point. Statistical significance was assessed using two-way ANOVA *t*-test (n = 3 independent biological replicates). n.s. (not significant).

F Representative microscopy images of WT and *zuo1Δ* cells expressing GFP-Atg8 on a plasmid and treated with 200 nM rapamycin for 4 h or left untreated. n = 4 biologically independent experiments. Scale bars = 3 μm.

G Graph displays the proportion of WT and *zuo1Δ* cells with GFP-Atg8 staining in the vacuole in both untreated cells and following 4 h treatment with 200 nM rapamycin. Data is presented as mean + s.d. n = 4 biologically independent experiments. Statistical significance was assessed using two-way ANOVA *t*-test.

Source data are available online for this figure.

agreement with observations made using nitrogen starvation (Fig 8C) (Kelly & Bedwell, 2015). As autophagy is required for eIF4G degradation, we assessed whether the loss of eIF4G degradation is due to a defect in the general autophagy pathway in *zuo1Δ* cells or if it is specific for eIF4G. The GFP-Atg8 reporter was utilised, which is delivered to the vacuole, alongside autophagosome cargo, where Atg8 is degraded. The GFP moiety is resistant to vacuolar hydrolases and so the accumulation of free GFP relative to full length GFP-Atg8 provides a measure of autophagic activity. A strong increase in free GFP was detected in WT cells expressing GFP-Atg8 in response to rapamycin treatment, which was absent in the autophagy-deficient *atg1Δ* cells (Fig 8D and E). In *zuo1Δ* cells, an increase in the free GFP signal was detected in response to rapamycin treatment but this was diminished in comparison to WT cells, indicating that there is a defect in bulk autophagy in *zuo1Δ* cells. Strong autophagy defect was also observed in *ssb1/2Δ* cells (Fig EV5). Additionally, GFP-Atg8 localisation was visualised. In untreated conditions, GFP-Atg8 is diffuse in the cytosol of both WT and *zuo1Δ* cells. Following rapamycin treatment, and autophagy induction, GFP-Atg8 was efficiently translocated to the vacuole in WT cells (Fig 8F and G). In contrast, the localisation of GFP-Atg8 to the vacuole was significantly reduced in *zuo1Δ* cells, indicating a defect in the formation of autophagosomes or their delivery to the vacuole (Fig 8F and G). Thus, the failure to reduce eIF4G levels in response to TORC1 inactivation in *zuo1Δ* cells is likely due to a deficiency in autophagy.

Discussion

We have examined the role of Zuo1 in maintaining proteostasis in response to TORC1 inhibition. Cells lacking Zuo1 have a major defect in translational regulation, leading to a loss of proteostasis and cell death upon TORC1 inhibition. Shutting down bulk translation in response to TORC1 inhibition helps conserve resources when conditions are not optimal for growth, and it is, therefore, likely that loss of this regulation in *zuo1Δ* cells is causing the reduced viability in response to rapamycin treatment. Polysome levels in *zuo1Δ* cells are lower in basal conditions compared to WT cells, but the overall rate of translation was not perturbed, as previously reported (Albanèse *et al*, 2010; Hanebuth *et al*, 2016). Zuo1 has been linked to translation previously, where it is involved in translational repression of polylysine proteins (Chiabudini *et al*, 2012) and, in mammalian cells, ribosome-associated chaperones have been linked to elongation pausing upon stress (Liu *et al*, 2013; Shalgi *et al*, 2013). It has also been proposed that this chaperone could slow down translation elongation to facilitate effective cotranslational protein folding (Zhang *et al*, 2014; Chen *et al*, 2022). It is, therefore, possible that, in the absence of Zuo1, translation elongation is proceeding faster than in WT cells, maintaining translation levels despite lower polysome levels. Failure to further decrease polysome levels upon challenge with rapamycin would therefore allow normal levels of translation to be sustained in *zuo1Δ* cells and disrupt its cellular proteostasis.

It appears that Zuo1 and Ssb cooperate to maintain proteostasis upon TORC1 inhibition. Surprisingly, though, the defect in reducing translation upon TORC1 inhibition is less severe in *zuo1Δssb1/2Δ* cells compared to *zuo1Δ* or *ssb1/2Δ* cells. Zuo1 and Ssb share binding sites on the ribosome with other ribosome-associated chaperones, including NAC (Pech *et al*, 2010; Gumiero *et al*, 2016; Gamerdinger *et al*, 2019). It is possible that in the complete absence of RAC/Ssb, NAC is able to interact more with ribosomes and partially substitute for the loss of this chaperone system. It has been shown that NAC from *C. elegans* competes for ribosome binding with RAC (Gamerdinger *et al*, 2019) and, in yeast, NAC is expressed at approximately equimolar levels to ribosomes but is not bound to all translating ribosomes (Raue *et al*, 2007). In this context, the autonomous ribosomal binding of either Ssb or Zuo1 in *zuo1Δ* and *ssb1/2Δ* cells, respectively, may preclude the interaction of NAC, preventing a functional chaperone system from operating at those ribosomes. However, whether NAC shows increased association with translating ribosomes in *zuo1Δssb1/2Δ* cells remains to be determined. Additionally, deletion of NAC enhances growth defects of *ssb1/2Δ* cells (Koplin *et al*, 2010), suggesting that these chaperones share overlapping functions. Further study is required to elucidate any interplay between these two chaperone systems for maintaining proteostasis upon TORC1 inhibition.

In response to rapamycin treatment or a variety of environmental stresses, the phosphorylation of Sui2 restricts global translation by reducing active ternary complex levels. However, *zuo1Δ* cells are not responsive to translational regulation following rapamycin treatment, despite Sui2 being phosphorylated in these cells. This is not

 © 2023 The Authors

unprecedented, as it has previously been shown that mRNA decay mutants are able to sustain translation in response to a variety of stresses which induce translational shutdown, despite an increase in eIF2α phosphorylation (Holmes *et al*, 2004). Although the mechanism has not been fully elucidated, it is thought that in these mutants, translation is maintained through increased eIF4F and mRNA interactions. In agreement with this, it was found that overexpression of eIF4G2 can prevent a decrease in polysomes, and active translation, in response to stress (Holmes *et al*, 2004). Zuo1 has not been implicated in mRNA decay, but it is possible that loss of Zuo1 may similarly confer resistance to translational inhibition by facilitating ongoing translation through the eIF4F complex.

In the absence of Zuo1, the levels of eIF4G are maintained through reduced autophagic degradation. How Zuo1 promotes the formation of autophagosomes or their delivery to the vacuole upon TORC1 inhibition is currently unclear. One possible scenario is that Zuo1 is required for the upregulation of autophagy-related proteins which occurs upon TORC1 inhibition, or for promoting the folding and stability of the essential autophagy machinery. It remains to be determined whether the formation of autophagosomes or their fusion with the vacuole is affected in *zuo1Δ* cells which will be the focus of future work and will provide clarity on the mechanism by which Zuo1 supports autophagy induction upon TORC1 inhibition. The failure to effectively upregulate autophagy and degrade eIF4G upon TORC1 inhibition likely contributes to loss of translational regulation and reduced cell viability. Inhibiting bulk translation is a key aspect of stress response under a large variety of stresses. This work has focused on TORC1 inhibition by the selective inhibitor rapamycin, but it would be useful to determine if Zuo1 plays a similar role in inhibiting translation following other stresses. This may indeed be the case as the deletion of Ssb prevents translation inhibition in response to glucose deprivation (von Plehwe *et al*, 2009). As we have seen that Zuo1's role relies on its ability to interact with Ssb, this chaperone complex may play a more ubiquitous role in regulating translation in response to stress.

Zuo1 is conserved in higher eukaryotes where it appears to share some functionality, as loss of this protein results in growth defects and hypersensitivity to aminoglycoside antibiotics, analogous to its loss in yeast (Shoji *et al*, 1995; Jaiswal *et al*, 2011; Aloia *et al*, 2014). The mammalian homologue has also been implicated in regulating translation in basal conditions and has been linked to mTORC1 signalling (Barilari *et al*, 2017; Wu *et al*, 2021). It is, therefore, possible that the role of Zuo1 in regulating translation in response to TORC1 inhibition is not confined to yeast. Loss of proteostasis is linked to ageing and disease progression, and so it will be important to understand the impact that the RAC complex plays in maintaining proteostasis under challenging conditions, including in cellular ageing and disease contexts, in higher organisms.

Materials and Methods

Reagents and Tools table

Reagent or Resource	Source	Identifier
Antibodies		
Mouse monoclonal anti-P4D1 (WB: 1:500)	Santa Cruz	Cat# sc-8017
Rabbit polyclonal anti-Phospho-S6 Ribosomal Protein (Ser235/236) (WB: 1:1,000)	Cell Signalling Technology	Cat #2211
Mouse anti-GFP (WB: 1:1,000)	Roche	Cat #11814460001
Mouse monoclonal anti-PGK1 (WB: 1:5,000)	Abcam	Cat #ab113687
Mouse monoclonal anti-Puromycin (WB: 1:500)	DSHB	Cat #PMY-2A4
Mouse monoclonal anti-FLAG M2 (WB: 1:2,000)	Sigma-Aldrich	Cat #F3165
Chemicals, peptides, and recombinant proteins		
Rapamycin	LC laboratories	Cat #R-5000
Cycloheximide	Santa Cruz	Cat #sc-3508A
Puromycin dihydrochloride	Santa Cruz	Cat #sc-108071B
Tunicamycin	Abcam	Cat ~ab120296
cOmplete, Mini, EDTA-free Protease Inhibitor Cocktail	Roche	Cat # 11836170001
SuperScript III Reverse Transcriptase	Thermo Fisher	Cat #18080044
PowerUp SYBR green Master mix	Thermo Fisher	Cat #A25776
GFP binder beads	MRC PPU Reagents and Services	DU54283
Ponceau S	Santa Cruz	Cat #sc-301558
Clarity Western ECL substrate	Bio-Rad	Cat #170-5061
Clarity Max Western ECL substrate	Bio-Rad	Cat #170-5062
TRIzol Reagent	Thermo Fisher	Cat #15596026

© 2023 The Authors

Reagents and Tools table (continued)

Reagent or Resource	Source	Identifier
GlycoBlue Coprecipitant	Thermo Fisher	Cat #AM9515
HiScribe T7 High Yield RNA synthesis Kit	New England Biolabs	Cat#E2040S
Critical commercial assays		
RNeasy Mini Kit	Qiagen	Cat #74104
Deposited data		
The mass spectrometry proteomics data have been deposited to the ProteomeXchange Consortium via the PRIDE partner repository with the dataset identifier PXD039550		
Experimental models: Organisms/strains		
Yeast strains used in this study are detailed in Table EV1		
Oligonucleotides		
Tif4631 F<br>tcagaagggacgctcctcct	Sigma	N/A
Tif4631 R<br>cctttggaggaggagccctt	Sigma	N/A
Tif4632 F<br>ctatgcgcggtatggaccat	Sigma	N/A
Tif4632 R<br>ttcgcgatcttttctggaggta	Sigma	N/A
Alg9 F<br>cacggatagtggctttggtgaacaattac	Sigma	N/A
Alg9 R<br>tatgattatctggcagcaggaaagaacttggg	Sigma	N/A
Recombinant DNA		
Plasmids used in this study are detailed in Table EV2		
Software and algorithms		
Prism 9.1.2	Graphpad	
Image J		https://imagej.nih.gov/ij/
Proteome Discoverer v.2.3	Thermo Fisher	

Methods and Protocols

Yeast strains, plasmids and growth conditions

All *Saccharomyces cerevisiae* strains used are isogenic to BY4741 and are listed in Table EV1. Plasmids used are listed in Table EV2 and were constructed using In-Fusion HD Cloning Plus. Gene deletions and genomic tagged strains were generated by homologous recombination as previously described (Janke *et al*, 2004). Genomic tagging with 5xFLAG was accomplished by PCR amplification of the 5xFLAG-His3MX cassette from pKL259. Integration of PCR cassettes was confirmed by PCR analysis. Yeast transformations were performed using the LiAc/PEG/SS carrier DNA method.

Yeast cells were cultured at 30°C in liquid medium with constant shaking at 200 rpm. Cells were cultured overnight in YEPD (1% yeast extract, 2% peptone, 2% glucose) or synthetic medium (2% glucose) lacking the appropriate amino acids for plasmid selection and then readjusted to OD600 0.2. Cells were grown to mid-log phase and then readjusted to OD 0.2 prior to treatment with 200 nM rapamycin, 37 μg/ml cycloheximide or 50 μM MG-132. In the case of treatment with 0.5 mM puromycin, yeast cells were readjusted to OD 0.3 instead of OD 0.2 on each occasion.

To assess growth, cells were equilibrated to OD_{600} 0.2 and either 5 μl alone or 5-fold serial dilutions were spotted onto YEPD plates or YEPD plates containing 20 ng/ml rapamycin or 0.75 μg/ml tunicamycin and incubated at 30°C for 4 days. The Chemidoc MP imaging system (Bio-Rad) was used to image plates. To assess growth in liquid culture, cells were adjusted to OD 0.2 in YEPD and treated with rapamycin or vehicle control. Cells were cultured in triplicate in 98-well plates at 30°C and OD_{600} measurements were taken every 30 min over the course of 24 h using the Fluostar Omega Microplate reader (BMG Labtech).

Yeast protein extraction and western blot analysis

Cells were harvested (3,900 *g*, 4°C, 4 min), washed in ice-cold water (8,000 rpm, 4°C, 1 min) and flash frozen in dry ice then stored at −20°C before extraction. Yeast pellets were resuspended in 400 μl 2 M LiAC, on ice and centrifuged at 6,000 *g* for 1 min. Pellets were then resuspended in 400 μl 0.4 M NaOH and centrifuged again before being resuspended in lysis buffer (0.1 M NaOH, 0.05 M EDTA, 2% SDS, 2% β-mercaptoethanol and cOmplete protease inhibitor cocktail) and boiled at 90°C for 10 min. PhosStop was included in the lysis buffer for the detection of phosphorylated

 © 2023 The Authors

proteins, and N-ethylmaleimide was included for the detection of polyubiquitinated proteins. Acetic acid (4 M) was then added (1:40), and samples were vortexed and boiled for an additional 10 min before centrifugation at 17,000 *g* for 15 min. The protein concentration of the supernatant was determined using a Nanodrop (A_{280nm}, Thermo). The supernatant was combined with 5× loading buffer (0.25 M Tris–HCl pH 6.8, 50% glycerol, 0.05% bromophenol blue) and all samples were adjusted to the same protein concentration.

Approximately 30 µg of each cell extract were separated on homemade 6–14% Bis-Tris acrylamide gradient gels (0.33 M Bis-Tris pH 6.5) at 120 V for 150 min at 4°C. Subsequently, gels were transferred onto 0.2 µM nitrocellulose membranes (Bio-Rad) at 25 V for 30 min using the Trans-blot Turbo transfer system (Bio-Rad). Ponceau S solution was used to stain membranes, which were imaged using the ChemiDoc MP system. Membranes were blocked in 5% (w/v) milk in TBS at room temperature for 1 h, washed in TBS, and incubated in primary antibodies overnight at 4°C. Then, membranes were washed in TBS-T and incubated with secondary antibodies for 1 h at room temperature prior to detection with Clarity ECL or Clarity Max ECL reagent using the ChemiDoc MP imaging system. Protein bands were quantified by densitometry using Image J, where indicated.

In-gel peptidase assay

Exponential phase yeasts were adjusted to an OD_{600} 0.2 in 30 ml and treated with 200 nM rapamycin for 3 h. Then, cells were harvested (3,900 *g*, 4°C, 4 min) and washed in ice-cold water and centrifuged (8,000 rpm, 4°C, 1 min). The cell pellet was resuspended in native lysis buffer (50 mM Tris pH 8.5, 5 mM $MgCl_2$, 0.5 mM EDTA, 5% glycerol, 1 mM DTT and 5 mM ATP) and lysed with glass beads (FastPrep 24, 3 × 30 s on/5 min off). The supernatant was then cleared by centrifugation at 17,000 *g* for 10 min at 4°C. Protein concentration was measured using a NanoDrop (A_{280nm}, Thermo) and samples were adjusted to an equal concentration in native sample buffer (50 mM Tris–HCl pH 6.8, 10% glycerol, 0.01% bromophenol blue). Samples were separated on 3.8–5% acrylamide native gel for 2 h at 120 V. Next gels were incubated with suc-LLVY-AMC fluorogenic substrate in assay buffer (50 mM Tris–HCl pH 7.5, 150 mM NaCl, 5 mM $MgCl_2$, 10% glycerol) at 30°C for 20 min in the dark and subsequently imaged using the ChemiDoc MP system (Bio-Rad). The gel was then incubated in assay buffer for an additional 10 min with the addition of 0.1% SDS and imaged again.

Polysome profiling

Polysome profile analysis was conducted as in Kasari *et al* (2019) with some minor modifications. Yeast strains were cultured in YEPD medium overnight, diluted to OD_{600} 0.4 and grown until the OD_{600} reached 0.8–0.9. The cultures were then diluted to OD_{600} 0.4 and treated with 200 nM rapamycin for 2 h. 60OD of each culture was then taken and incubated with 100 µg/ml cycloheximide on ice for 10 min. Cells were centrifuged at 4,000 *g* for 5 min at 4°C, washed in ice-cold buffer I (20 mM Tris–HCl, pH 7.4, 15 mM $MgCl_2$, 100 mM KCl, 100 µg/ml CHX) and centrifuged again. Next, the pellet was lysed in 250 µl of buffer I supplemented with 1 mM DTT, and EDTA-free protease inhibitor cocktail using 250 µl of acid washed glass beads and by vortexing (4 × 30 s, 4°C). Lysates were clarified by centrifugation at 17,000 *g* for 15 min at 4°C. Seven A_{260} units were loaded onto 10–50% sucrose gradients, prepared using a Biocomp Gradient master instrument, in Buffer I containing 1 mM DTT. The gradients were centrifuged at 209,790 *g* for 3 h at 4°C (SW41 Ti rotor; Beckman) and subsequently fractionated using a Piston Gradient Fractionator (Biocomp instruments) monitoring absorbance at 260 nm.

RNA isolation from gradient fractions

Isolation of RNA from the fractions obtained from polysome profiling was carried as previously described (Pringle *et al*, 2019). Firefly luciferase (FLuc) RNA was used as a control to normalise for RNA recovery from each fraction. One volume of TRIzol was added to one volume of each fraction and mixed by pipetting. The samples were incubated at room temperature for 5 min and then 200 µl of chloroform per 500 µl of gradient fraction was added. This mixture was centrifuged at 12,000 *g* for 15 min at 4°C. The aqueous phase was transferred to a fresh microcentrifuge tube and an equal volume isopropanol was added along with 60 µg GlycoBlue and 500 pg of FLuc RNA (per 500 µl of gradient fraction). This was vortexed to mix and incubated overnight at −20°C. The next day, the samples were centrifuged at 12,000 *g* for 15 min at 4°C. The supernatant was then discarded and 1 ml of ice-cold 70% ethanol was added to the pellet prior to centrifugation at 12,000 *g* for 5 min. The supernatant was discarded again, and the pellet was dried at 95°C for ~5 min. Finally, the RNA was solubilised in 20 µl of water and then analysed by RT-qPCR or stored at −80°C.

Immunoprecipitation

Exponential phase yeasts were adjusted to an OD_{600} 0.2 in 30 ml and treated with 200 nM rapamycin for 3 h. Then, cells were harvested (400 rpm, 4°C, 4 min) and washed in ice-cold water and centrifuged (8,000 rpm, 4°C, 1 min). The cell pellet was resuspended in IP lysis buffer (50 mM Tris–HCl pH 8.0, 100 mM NaCl, 1 mM EDTA, 5 mM $MgCl_2$, 1 mM DTT, 10% glycerol, 0.5 mM PMSF and cOmplete protease inhibitor cocktail) and lysed with glass beads (FastPrep 24, 3 × 30 s on/5 min off). The supernatant was then cleared by centrifugation at 17,000 *g* for 10 min at 4°C. Protein concentration was measured using a NanoDrop (A_{280nm}, Thermo) and samples were equally adjusted to 1 mg/ml in IP lysis buffer. GFP binder beads (25 µl slurry per sample; MRC PPU reagents and services: DU54283) were equilibrated into 500 µl ice-cold wash buffer (10 mM Tris–HCl pH 7.5, 150 mM NaCl and 0.5 mM EDTA) before being centrifuged at 2,500 *g* for 2 min at 4°C. Supernatant was discarded and equilibration steps repeated twice. Equilibrated beads were incubated with 1 mg of lysates for 1 h at 4°C under rotation. GFP binder beads were then washed once with lysis buffer, twice with wash buffer. Beads were subjected to tryptic digestion and TMT-based quantitative proteomics (see below).

On-bead tryptic digestion

Beads were collected and subsequently resuspended in 50 µl Urea buffer (2 M urea, 50 mM ammonium bicarbonate pH 8.0 and 5 mM DTT) and incubated at 45°C for 30 min with gentle shaking in order to partially denature the proteins. Samples were centrifuged at 2,500 *g* for 1 min and cooled to room temperature. Each sample was then incubated with iodoacetamide (10 mM final concentration) in the dark at room temperature. Unreacted iodoacetamide was then quenched with DTT (5 mM final concentration). Each sample was digested using 0.4 µg trypsin at 37°C for 4 h under

© 2023 The Authors

agitation. The digestion was stopped by adding trifluoroacetic acid (TFA) to the final 0.2% TFA concentration (v/v), centrifuged at 10,000 *g* for 2 min at room temperature. The supernatant was de-salted on ultra-microspin column silica C18 (The Nest Group). De-salted peptides were dried using a SpeedVac vacuum centrifuge concentrator (Thermo Fisher) before TMT labelling.

TMT labelling

Each vacuum-dried sample was resuspended in 50 µl of 100 mM TEAB buffer. The TMT labelling reagents were equilibrated to room temperature, and 41 µl anhydrous acetonitrile was added to each reagent channel and gently vortexed for 10 min. Then, 4 µl of each TMT reagent was added to the corresponding sample and labelling was performed at room temperature for 1 h with shaking before quenching with 1 µl of 5% hydroxylamine, after which 2 µl of labelled sample from each channel was analysed by liquid chromatography with tandem mass spectrometry (LC–MS/MS) to ensure complete labelling before mixing. After evaluation, the complete TMT-labelled samples were combined, acidified and dried. The mixture was then de-salted with ultra-microspin column silica C18, and the eluent from C18 column was dried.

LC–MS/MS analysis

LC separations were performed with a Thermo Dionex Ultimate 3000 RSLC Nano liquid chromatography instrument. The dried peptides were dissolved in 0.1% formic acid and then loaded on C18 trap column with 3% ACN/0.1%TFA at a flow rate of 10 µl/min. Peptide separations were performed using EASY-Spray columns (C18, 2 µm, 75 µm × 50 cm) with an integrated nano electrospray emitter at a flow rate of 300 nl/min. Peptides were separated with a 180-min segmented gradient as follows starting from 8 to 30% buffer B in 135 min, ~30–45% in 20 min and ~45–90% in 5 min. Peptides eluted from the column were analysed on an Orbitrap Fusion Lumos (Thermo Fisher Scientific) mass spectrometer. Spray voltage was set to 2 kV, RF lens level was set at 30%, and ion transfer tube temperature was set to 275°C. The Orbitrap Fusion Lumos was operated in positive-ion data-dependent mode with synchronous precursor selection (SPS)-MS3. The mass spectrometer was operated in data-dependent top speed mode with 3 s per cycle. The full scan was performed in the range of 350–1,500 m/z at nominal resolution of 120,000 at 200 m/z and AGC set to 4 × 105 with maximum injection time 50 ms, followed by selection of the most intense ions above an intensity threshold of 5 × 103 for collision-induced dissociation (CID)-MS2 fragmentation with 35% normalised collision energy. The isolation width was set to 0.7 m/z with no offset. Dynamic exclusion was set to 60 s. Monoisotopic precursor selection was set to peptide. Charge states between 2 and 7 were included for MS2 fragmentation. The MS2 scan was performed in the ion trap with auto normal range scan and AGC target of 1 × 104. The maximum injection time for MS2 scan was set to 50 ms. For the MS3 scan, SPS was enabled. MS3 was performed in the Orbitrap over 5 notches at a resolution of 50,000 at 200 m/z and AGC set to 5 × 104 with maximum injection time 105 ms, over a mass range of 100–500 m/z, with high collision-induced dissociation (HCD) and 65% normalised collision energy.

Proteomic data analysis

All the acquired LC–MS data were analysed using Proteome Discoverer v.2.3 (Thermo Fisher Scientific) with Mascot search engine. Maximum missed cleavage for trypsin digestion was set to 2. Precursor mass tolerance was set to 10 ppm. Fragment ion tolerance was set to 0.2 Da. Carbamidomethylation on cysteine (+57.021 Da) and TMT-10plex tags on N-termini as well as lysine (+229.163 Da) were set as static modifications. Variable modifications were set as oxidation on methionine (+15.995 Da). Data were searched against SwissProt database restricted to Saccharomyces cerevisiae taxonomy (reviewed 7,905 entries downloaded in June 2019). Peptide spectral match error rates with a 1% false discovery rate were determined using the forward decoy strategy modelling true and false matches.

Both unique and razor peptides were used for quantitation. Reporter ion abundances were corrected for isotopic impurities on the basis of the manufacturer's data sheets. Reporter ions were quantified from MS3 scans using an integration tolerance of 20 ppm with the most confident centroid setting. Signal-to-noise (S/N) values were used to represent the reporter ion abundance with a co-isolation threshold of 50% and an average reporter S/N threshold of 10 and above required for quantitation from each MS3 spectra to be used. The summed abundance of quantified peptides was used for protein quantitation. The total peptide amount was used for the normalisation. Protein ratios were calculated from medians of summed sample abundances of replicate groups. Standard deviation was calculated from all biological replicate values. The standard deviation of all biological replicates lower than 25% was used for further analyses. Multiple unpaired *t*-test was used to determine the significant differences between untreated and rapamycin-treated conditions.

qRT-PCR

Total RNA from cells grown in the presence or absence of 200 nM rapamycin for 2 h was extracted using the RNeasy Kit according to the manufacturer's instructions. Synthesis of cDNA from 100 ng of total RNA was carried out using SuperScript III reverse transcriptase and random primers. qRT-PCR analysis was performed on a CFX384 real-time PCR detection system (Bio-Rad) using PowerUp SYBR Green Master Mix. Primer sequences are listed in the key resources table. The *ALG9* mRNA was used to normalise gene expression in each sample and changes in gene expression after rapamycin treatment were calculated using the $\Delta\Delta C_t$ method.

Fluorescence microscopy

Yeast was grown on HIS media overnight at 30°C, resuspended to OD600nm 0.2 in YEPD medium and grown at 30°C to OD600nm 0.5–0.7. Cultures were split into 2-ml samples in glass bottom dishes and treated or not with rapamycin (200 nM final) for 4 h before being imaged on a Zeiss 880 Airyscan microscope (Airyscan mode, Alpha Plan-APO 63×/1.4 oil objective (Zeiss)) at 30°C. ZEN 2.3 SP1 FP3 software was used to acquire images. All microscopy analyses were carried out using FIJI. For quantification of autophagic cells, cells were considered autophagic when the vacuolar GFP signal from GFP-ATG8 was higher than the cytosolic signal. Quantification included at least 400 cells per conditions.

Statistical analysis

Each experiment was repeated independently a minimum of three times, as indicated. The standard deviation (s.d.) of the mean of at least three independent experiments is shown in the graphs, unless otherwise specified. *P* values are as stated, or not significant (NS).

 © 2023 The Authors

P values, numbers of independent biological replicates and choice of statistical tests are described in individual figure legends. All statistics were performed using Graph Pad Prism 9 software (version 9.1.2) (Graph Pad Software Inc.). No statistical method was used to pre-determine sample size. No data were excluded from the analyses, and the experiments were not randomised.

Data availability

All the data generated or analysed during the current study are included in this published article and its supplementary files. The mass spectrometry proteomics data have been deposited to the ProteomeXchange Consortium via the PRIDE partner repository with the dataset identifier PXD039550 (https://proteomecentral.proteomexchange.org/cgi/GetDataset?ID=PXD039550). All other data supporting the findings of this study are available from the corresponding author on reasonable request. This paper does not report original code.

Expanded View for this article is available online.

Acknowledgements
We thank the MRC PPU reagents and services, including Cloning team and DNA sequencing services, MRC-PPU Mass Spectrometry and the Dundee Imaging facility. This research was supported by the Medical Research Council (grant number MC_UU_00018/8 to AR).

Author contributions
Ailsa Black: Conceptualization; data curation; software; formal analysis; investigation; visualization; methodology; writing – original draft; writing – review and editing. **Thomas D Williams:** Data curation; methodology; writing – review and editing. **Flavie Soubigou:** Data curation; methodology; writing – review and editing. **Ifeoluwapo M Joshua:** Data curation; methodology; writing – review and editing. **Houjiang Zhou:** Software; formal analysis; methodology. **Frederic Lamoliatte:** Formal analysis; methodology. **Adrien Rousseau:** Conceptualization; data curation; software; supervision; funding acquisition; investigation; visualization; methodology; project administration; writing – review and editing.

Disclosure and competing interests statement
The authors declare that they have no conflict of interest.

References

Advani VM, Ivanov P (2019) Translational control under stress: reshaping the translatome. *Bioessays* 41: e1900009

Albanèse V, Reissmann S, Frydman J (2010) A ribosome-anchored chaperone network that facilitates eukaryotic ribosome biogenesis. *J Cell Biol* 189: 69–81

Aloia L, Di Stefano B, Sessa A, Morey L, Santanach A, Gutierrez A, Cozzuto L, Benitah SA, Graf T, Broccoli V *et al* (2014) Zrf1 is required to establish and maintain neural progenitor identity. *Genes Dev* 28: 182–197

Ashe MP, De Long SK, Sachs AB (2000) Glucose depletion rapidly inhibits translation initiation in yeast. *Mol Biol Cell* 11: 833–848

Barilari M, Bonfils G, Treins C, Koka V, De Villeneuve D, Fabrega S, Pende M (2017) ZRF1 is a novel S6 kinase substrate that drives the senescence programme. *EMBO J* 36: 736–750

Ben-Sahra I, Manning BD (2017) mTORC1 signaling and the metabolic control of cell growth. *Curr Opin Cell Biol* 45: 72–82

Berset C, Trachsel H, Altmann M (1998) The TOR (target of rapamycin) signal transduction pathway regulates the stability of translation initiation factor eIF4G in the yeast *Saccharomyces cerevisiae*. *Proc Natl Acad Sci USA* 95: 4264–4269

Cardenas ME, Cutler NS, Lorenz MC, Di Como CJ, Heitman J (1999) The TOR signaling cascade regulates gene expression in response to nutrients. *Genes Dev* 13: 3271–3279

Causton HC, Ren B, Koh SS, Harbison CT, Kanin E, Jennings EG, Lee TI, True HL, Lander ES, Young RA (2001) Remodeling of yeast genome expression in response to environmental changes. *Mol Biol Cell* 12: 323–337

Chen Y, Tsai B, Li N, Gao N (2022) Structural remodeling of ribosome associated Hsp40-Hsp70 chaperones during co-translational folding. *Nat Commun* 13: 3410

Chiabudini M, Conz C, Reckmann F, Rospert S (2012) Ribosome-associated complex and Ssb are required for translational repression induced by polylysine segments within nascent chains. *Mol Cell Biol* 32: 4769–4779

Dephoure N, Gygi SP (2012) Hyperplexing: a method for higher-order multiplexed quantitative proteomics provides a map of the dynamic response to rapamycin in yeast. *Sci Signal* 5: rs2

Döring K, Ahmed N, Riemer T, Suresh HG, Vainshtein Y, Habich M, Riemer J, Mayer MP, O'Brien EP, Kramer G *et al* (2017) Profiling Ssb-nascent chain interactions reveals principles of Hsp70-assisted folding. *Cell* 170: 298–311

Eisenman HC, Craig EA (2004) Activation of pleiotropic drug resistance by the J-protein and Hsp70-related proteins, Zuo1 and Ssz1. *Mol Microbiol* 53: 335–344

Gamerdinger M, Kobayashi K, Wallisch A, Kreft SG, Sailer C, Schlömer R, Sachs N, Jomaa A, Stengel F, Ban N *et al* (2019) Early scanning of nascent polypeptides inside the ribosomal tunnel by NAC. *Mol Cell* 75: 996–1006

Gautschi M, Mun A, Ross S, Rospert S (2002) A functional chaperone triad on the yeast ribosome. *Proc Natl Acad Sci USA* 99: 4209–4214

Ghosh A, Shcherbik N (2020) Cooperativity between the ribosome-associated chaperone Ssb/RAC and the ubiquitin ligase Ltn1 in ubiquitination of nascent polypeptides. *Int J Mol Sci* 21: 6815

Gilbert WV, Zhou K, Butler TK, Doudna JA (2007) Cap-independent translation is required for starvation-induced differentiation in yeast. *Science* 317: 1224–1227

Gillies AT, Taylor R, Gestwicki JE (2012) Synthetic lethal interactions in yeast reveal functional roles of J protein co-chaperones. *Mol Biosyst* 8: 2901–2908

Gumiero A, Conz C, Gesé GV, Zhang Y, Weyer FA, Lapouge K, Kappes J, von Plehwe U, Schermann G, Fitzke E *et al* (2016) Interaction of the cotranslational Hsp70 Ssb with ribosomal proteins and rRNA depends on its lid domain. *Nat Commun* 7: 13563

Hanebuth MA, Kityk R, Fries SJ, Jain A, Kriel A, Albanese V, Frickey T, Peter C, Mayer MP, Frydman J *et al* (2016) Multivalent contacts of the Hsp70 Ssb contribute to its architecture on ribosomes and nascent chain interaction. *Nat Commun* 7: 13695

Hardwick JS, Kuruvilla FG, Tong JK, Shamji AF, Schreiber SL (1999) Rapamycin-modulated transcription defines the subset of nutrient-sensitive signaling pathways directly controlled by the Tor proteins. *Proc Natl Acad Sci USA* 96: 14866–14870

Hartl FU, Bracher A, Hayer-Hartl M (2011) Molecular chaperones in protein folding and proteostasis. *Nature* 475: 324–332

Hinnebusch AG, Lorsch JR (2012) The mechanism of eukaryotic translation initiation: new insights and challenges. *Cold Spring Harb Perspect Biol* 4: a011544

© 2023 The Authors

Holmes LEA, Campbell SG, De Long SK, Sachs AB, Ashe MP (2004) Loss of translational control in yeast compromised for the major mRNA decay pathway. *Mol Cell Biol* 24: 2998 – 3010

Huang P, Gautschi M, Walter W, Rospert S, Craig EA (2005) The Hsp70 Ssz1 modulates the function of the ribosome-associated J-protein Zuo1. *Nat Struct Mol Biol* 12: 497 – 504

Hundley H, Eisenman H, Walter W, Evans T, Hotokezaka Y, Wiedmann M, Craig E (2002) The in vivo function of the ribosome-associated Hsp70, Ssz1, does not require its putative peptide-binding domain. *Proc Natl Acad Sci USA* 99: 4203 – 4208

Jaiswal H, Conz C, Otto H, Wölfle T, Fitzke E, Mayer MP, Rospert S (2011) The chaperone network connected to human ribosome-associated complex. *Mol Cell Biol* 31: 1160 – 1173

Janke C, Magiera MM, Rathfelder N, Taxis C, Reber S, Maekawa H, Moreno-Borchart A, Doenges G, Schwob E, Schiebel E *et al* (2004) A versatile toolbox for PCR-based tagging of yeast genes: new fluorescent proteins, more markers and promoter substitution cassettes. *Yeast* 21: 947 – 962

Kamada Y, Yoshino K, Kondo C, Kawamata T, Oshiro N, Yonezawa K, Ohsumi Y (2010) Tor directly controls the Atg1 kinase complex to regulate autophagy. *Mol Cell Biol* 30: 1049 – 1058

Kapp LD, Lorsch JR (2004) GTP-dependent recognition of the methionine moiety on initiator tRNA by translation factor eIF2. *J Mol Biol* 335: 923 – 936

Kasari V, Margus T, Atkinson GC, Johansson MJO, Hauryliuk V (2019) Ribosome profiling analysis of eEF3-depleted *Saccharomyces cerevisiae*. *Sci Rep* 9: 3037

Kaschner LA, Sharma R, Shrestha OK, Meyer AE, Craig EA (2015) A conserved domain important for association of eukaryotic J-protein co-chaperones Jjj1 and Zuo1 with the ribosome. *Biochim Biophys Acta* 1853: 1035 – 1045

Kelly SP, Bedwell DM (2015) Both the autophagy and proteasomal pathways facilitate the Ubp3p-dependent depletion of a subset of translation and RNA turnover factors during nitrogen starvation in *Saccharomyces cerevisiae*. *RNA* 21: 898 – 910

Klaips CL, Jayaraj GG, Hartl FU (2017) Pathways of cellular proteostasis in aging and disease. *J Cell Biol* 217: 51 – 63

Kolkman A, Daran-Lapujade P, Fullaondo A, Olsthoorn MMA, Pronk JT, Slijper M, Heck AJR (2006) Proteome analysis of yeast response to various nutrient limitations. *Mol Syst Biol* 2: 2006.0026

Koplin A, Preissler S, Ilina Y, Koch M, Scior A, Erhardt M, Deuerling E (2010) A dual function for chaperones SSB-RAC and the NAC nascent polypeptide-associated complex on ribosomes. *J Cell Biol* 189: 57 – 68

Krishnamoorthy T, Pavitt GD, Zhang F, Dever TE, Hinnebusch AG (2001) Tight binding of the phosphorylated alpha subunit of initiation factor 2 (eIF2alpha) to the regulatory subunits of guanine nucleotide exchange factor eIF2B is required for inhibition of translation initiation. *Mol Cell Biol* 21: 5018 – 5030

Labbadia J, Morimoto RI (2015) The biology of proteostasis in aging and disease. *Annu Rev Biochem* 84: 435 – 464

Lippman SI, Broach JR (2009) Protein kinase A and TORC1 activate genes for ribosomal biogenesis by inactivating repressors encoded by Dot6 and its homolog Tod6. *Proc Natl Acad Sci USA* 106: 19928 – 19933

Liu B, Han Y, Qian S-B (2013) Cotranslational response to proteotoxic stress by elongation pausing of ribosomes. *Mol Cell* 49: 453 – 463

Medicherla B, Kostova Z, Schaefer A, Wolf DH (2004) A genomic screen identifies Dsk2p and Rad23p as essential components of ER-associated degradation. *EMBO Rep* 5: 692 – 697

Melamed D, Pnueli L, Arava Y (2008) Yeast translational response to high salinity: global analysis reveals regulation at multiple levels. *RNA* 14: 1337 – 1351

Pech M, Spreter T, Beckmann R, Beatrix B (2010) Dual binding mode of the nascent polypeptide-associated complex reveals a novel universal adapter site on the ribosome. *J Biol Chem* 285: 19679 – 19687

Powers T, Walter P (1999) Regulation of ribosome biogenesis by the rapamycin-sensitive TOR-signaling pathway in *Saccharomyces cerevisiae*. *Mol Biol Cell* 10: 987 – 1000

Preiss T, Baron-Benhamou J, Ansorge W, Hentze MW (2003) Homodirectional changes in transcriptome composition and mRNA translation induced by rapamycin and heat shock. *Nat Struct Mol Biol* 10: 1039 – 1047

Pringle ES, McCormick C, Cheng Z (2019) Polysome profiling analysis of mRNA and associated proteins engaged in translation. *Curr Protoc Mol Biol* 125: e79

Raue U, Oellerer S, Rospert S (2007) Association of protein biogenesis factors at the yeast ribosomal tunnel exit is affected by the translational status and nascent polypeptide sequence. *J Biol Chem* 282: 7809 – 7816

Rousseau A, Bertolotti A (2016) An evolutionarily conserved pathway controls proteasome homeostasis. *Nature* 536: 184 – 189

Rousseau A, Bertolotti A (2018) Regulation of proteasome assembly and activity in health and disease. *Nat Rev Mol Cell Biol* 19: 697 – 712

Schmidt EK, Clavarino G, Ceppi M, Pierre P (2009) SUnSET, a nonradioactive method to monitor protein synthesis. *Nat Methods* 6: 275 – 277

Shalgi R, Hurt JA, Krykbaeva I, Taipale M, Lindquist S, Burge CB (2013) Widespread regulation of translation by elongation pausing in heat shock. *Mol Cell* 49: 439 – 452

Shenton D, Smirnova JB, Selley JN, Carroll K, Hubbard SJ, Pavitt GD, Ashe MP, Grant CM (2006) Global translational responses to oxidative stress impact upon multiple levels of protein synthesis. *J Biol Chem* 281: 29011 – 29021

Shoji W, Inoue T, Yamamoto T, Obinata M (1995) MIDA1, a protein associated with Id, regulates cell growth. *J Biol Chem* 270: 24818 – 24825

Simpson CE, Ashe MP (2012) Adaptation to stress in yeast: to translate or not? *Biochem Soc Trans* 40: 794 – 799

von der Haar T, McCarthy JEG (2002) Intracellular translation initiation factor levels in Saccharomyces cerevisiae and their role in cap-complex function. *Mol Microbiol* 46: 531 – 544

von Plehwe U, Berndt U, Conz C, Chiabudini M, Fitzke E, Sickmann A, Petersen A, Pfeifer D, Rospert S (2009) The Hsp70 homolog Ssb is essential for glucose sensing via the SNF1 kinase network. *Genes Dev* 23: 2102 – 2115

Waite KA, Burris A, Vontz G, Lang A, Roelofs J (2022) Proteaphagy is specifically regulated and requires factors dispensable for general autophagy. *J Biol Chem* 298: 101494

Williams TD, Rousseau A (2022) Actin dynamics in protein homeostasis. *Biosci Rep* 42: BSR20210848

Williams TD, Cacioppo R, Agrotis A, Black A, Zhou H, Rousseau A (2022) Actin remodelling controls proteasome homeostasis upon stress. *Nat Cell Biol* 24: 1 – 11

Williams TD, Winaya AR, Rousseau A (2023) Proteasome assembly chaperone translation upon stress requires Ede1 phase separation at the plasma membrane. doi: 10.2139/ssrn.4499056 [PREPRINT]

Willmund F, del Alamo M, Pechmann S, Chen T, Albanèse V, Dammer EB, Peng J, Frydman J (2013) The cotranslational function of ribosome-associated Hsp70 in eukaryotic protein homeostasis. *Cell* 152: 196 – 209

Wu I-H, Yoon JS, Yang Q, Liu Y, Skach W, Thomas P (2021) A role for the ribosome-associated complex in activation of the IRE1 branch of UPR. *Cell Rep* 36: 109366

 © 2023 The Authors

Yerlikaya S, Meusburger M, Kumari R, Huber A, Anrather D, Costanzo M, Boone C, Ammerer G, Baranov PV, Loewith R (2016) TORC1 and TORC2 work together to regulate ribosomal protein S6 phosphorylation in *Saccharomyces cerevisiae*. *Mol Biol Cell* 27: 397 – 409

Zhang Y, Ma C, Yuan Y, Zhu J, Li N, Chen C, Wu S, Yu L, Lei J, Gao N (2014) Structural basis for interaction of a cotranslational chaperone with the eukaryotic ribosome. *Nat Struct Mol Biol* 21: 1042 – 1046

Zhang Y, Sinning I, Rospert S (2017) Two chaperones locked in an embrace: structure and function of the ribosome-associated complex RAC. *Nat Struct Mol Biol* 24: 611 – 619

License: This is an open access article under the terms of the Creative Commons Attribution License, which permits use, distribution and reproduction in any medium, provided the original work is properly cited.

Expanded View Figures

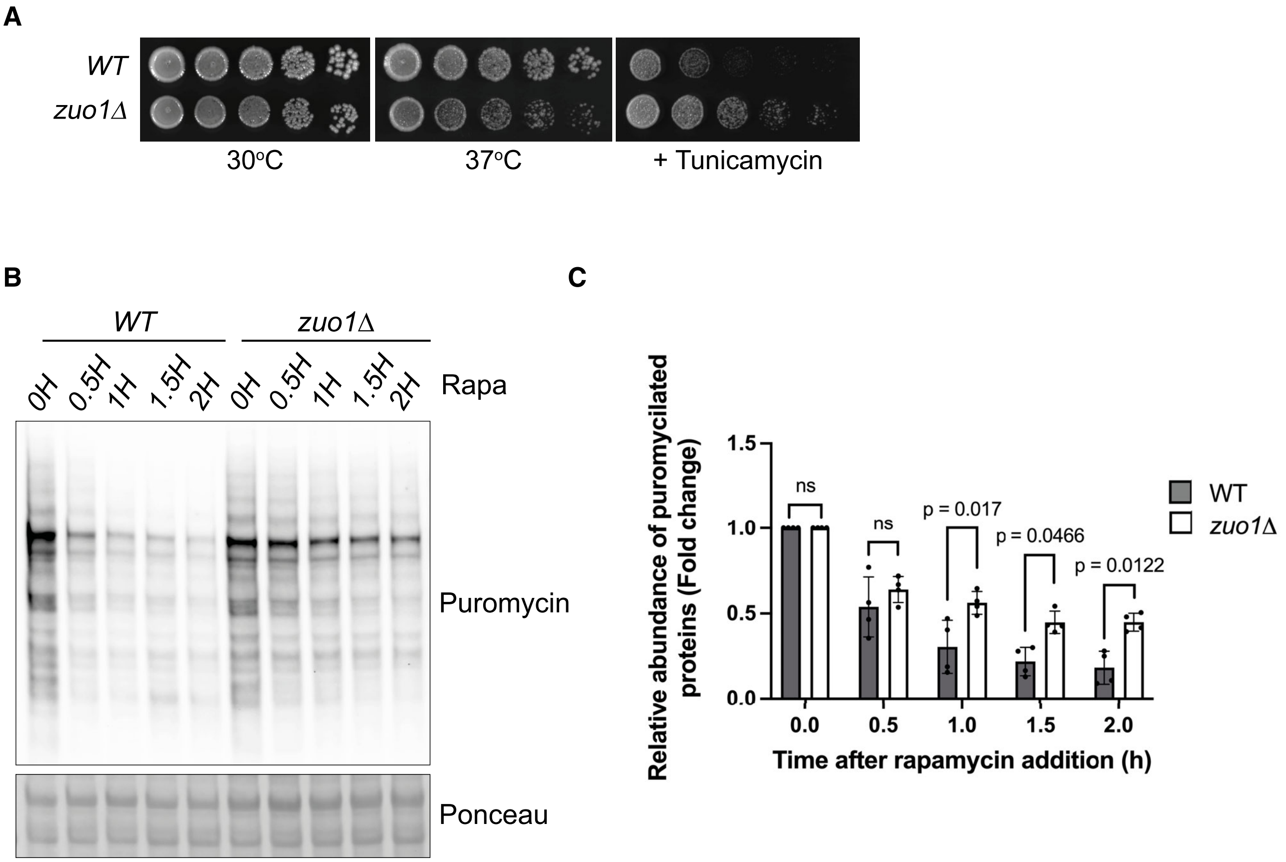

Figure EV1. Sensitivity of Zuo1 to other stresses.

A Fivefold serial dilutions of the indicated strains grown on YEPD plates with or without 0.75 μg/ml tunicamycin for 4 days at 30 or 37°C, where indicated.

B Immunoblot analysis of lysates from WT and *zuo1Δ* cells pulse-chased with 0.5 mM puromycin for 15 min before being treated with 35 μg/ml cycloheximide for the indicated time. Ponceau staining served as the loading control.

C Graph shows densitometry analysis (mean ± s.d.) of the relative abundance of puromycylated proteins (normalised to Pgk1 levels) from (B) relative to the 0H time point. Statistical significance was assessed using two-way ANOVA *t*-test ($n = 4$ independent biological replicates). n.s. (not significant).

Source data are available online for this figure.

 © 2023 The Authors

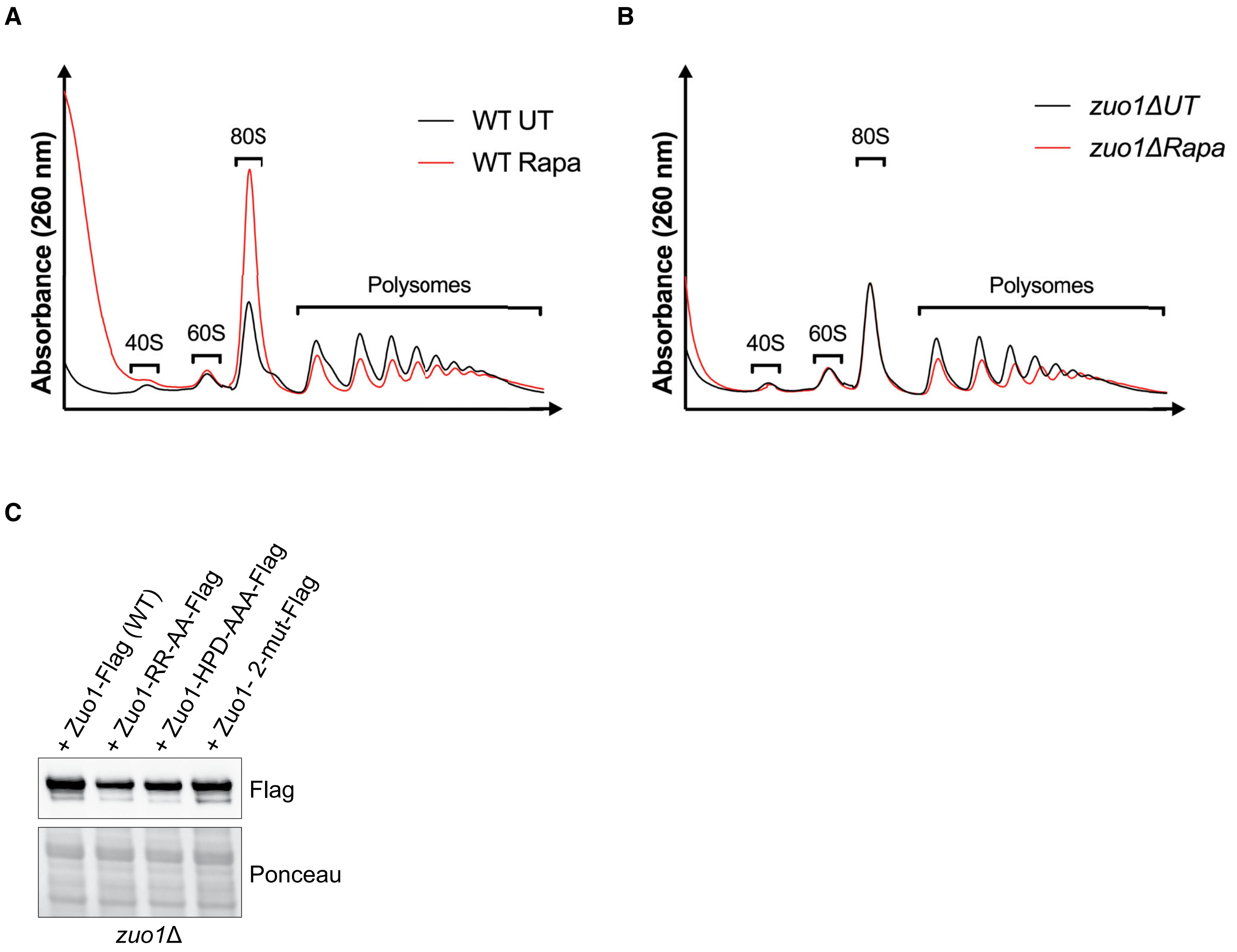

Figure EV2. Polysome profiling analysis of WT and *zuo1*Δ cells.

A, B Polysome profiles of WT (A) and *zuo1*Δ (B) strains either treated with 200 nM rapamycin for 2 h or left untreated. Total extracts were separated on 10–50% sucrose gradients and subsequently the A_{260} was monitored during fractionation.

C Immunoblot analysis of lysates from *zuo1Δ* cells expressing Flag-tagged WT and mutants Zuo1 on a plasmid. Ponceau staining served as the loading control.

Source data are available online for this figure.

Figure EV3. Gene ontology analysis of Zuo1 interactors.

A Protein–protein interaction network of Zuo1 interactors having at least 50% peptide coverage.

B Gene ontology analysis of Zuo1 interactors having at least 50% peptide coverage.

C Table showing Zuo1 interactors having their total protein level significantly increased or decreased by 1.5-fold based on multiplexed quantitative proteomics (https://www.science.org/doi/10.1126/scisignal.2002548). Zuo1 interactors more and less bound to Zuo1-GFP upon rapamycin treatment are shown in red and blue respectively.

D Immunoblot analysis of lysates from WT cells containing Ura7-5xFLAG at the endogenous locus treated with 200 nM rapamycin for 2 h or left untreated. Ponceau staining served as the loading control.

Source data are available online for this figure.

▶

© 2023 The Authors

A

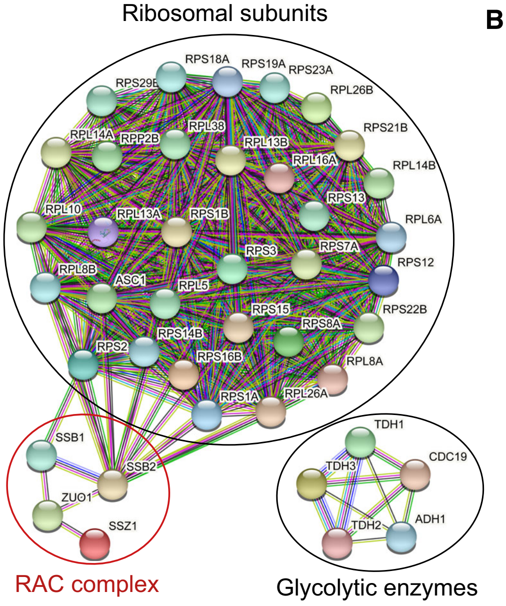

B

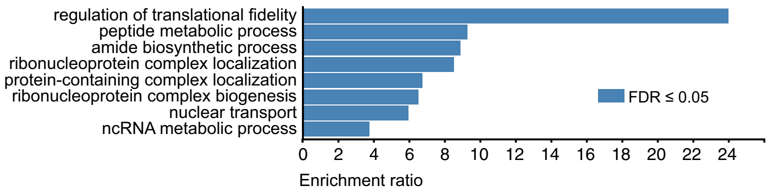

C

Hits induced by rapamycin	Hits unafected by rapamycin	Hits reduced by rapamycin	Hits not detected
CPS1	LSP1	TIF4631	ARO10
CPA2	HYR1	URA7	BAT2
TDH1	TRP3		ADH5
AIM41	VPS4		SNA2
HSP26	FMP52		
PET10	GTT1		
SDH1	DAK1		
SRY1	ADE8		
HFD1	PIL1		
SDH2	TUM1		
PEP4	TDH2		
ALT1	ACO1		
TSL1	APA1		
GGA1	LYS20		
PNC1	MAE1		
DLD3			
HSP78			
DUR1,2			
DCS1			
PYC1			
MPM1			
LYS21			

D

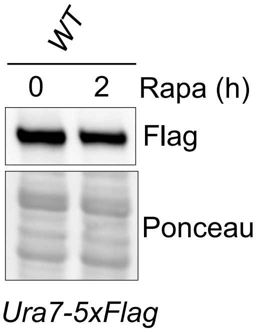

Figure EV3.

© 2023 The Authors

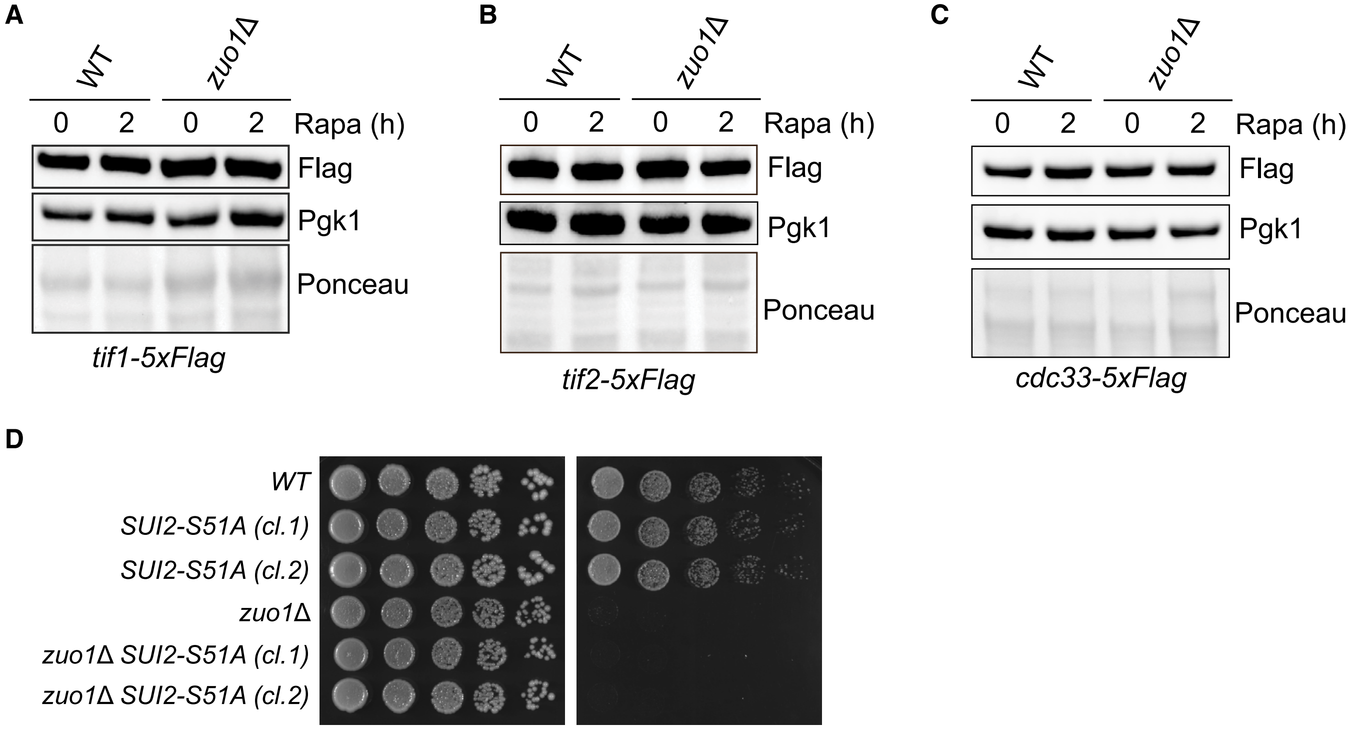

Figure EV4. Abundance of eIF4F subunits upon TORC1 inhibition.

A–C Immunoblot analysis of lysates from WT and *zuo1Δ* cells containing TIF1-5xFLAG (A), TIF2-5xFLAG (B), or CDC33-5xFLAG (C) at the endogenous locus treated with 200 nM rapamycin for 2 h or left untreated. Ponceau and Pgk1 staining served as the loading control.

D Fivefold serial dilutions of the indicated strains grown on YEPD plates with or without 20 ng/ml rapamycin for 4 days at 30°C.

Source data are available online for this figure.

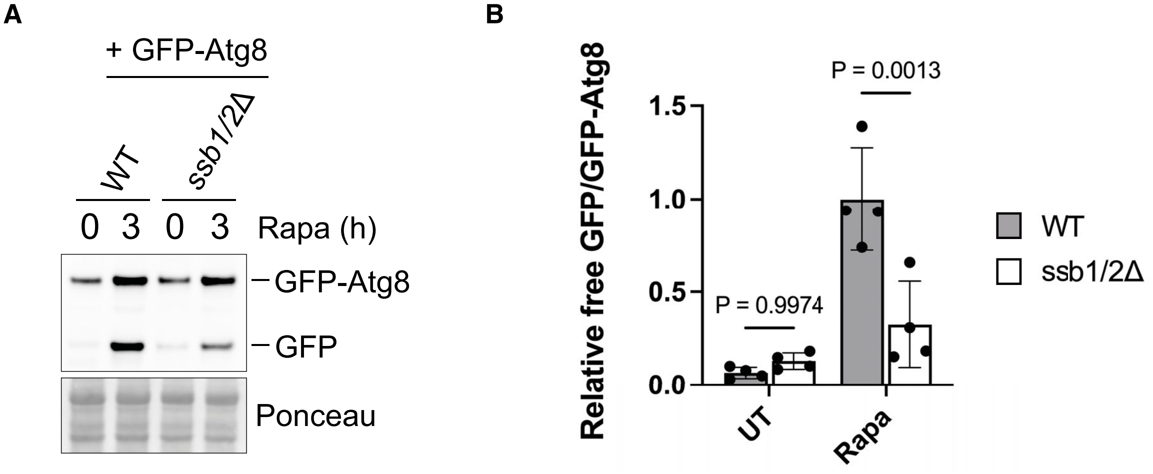

Figure EV5. Autophagy analysis in *ssb1/2Δ* cells.

A Immunoblot analysis of lysates from WT and *ssb1/2Δ* cells expressing GFP-Atg8 on a plasmid treated with 200 nM rapamycin for 3 h or left untreated. Ponceau staining served as the loading control.

B Graph shows densitometry analysis (mean ± s.d.) of the relative abundance of GFP-Atg8 and free GFP from (A), relative to the 0 h time point. Statistical significance was assessed using two-way ANOVA *t*-test (n = 4 independent biological replicates). n.s. (not significant).

Source data are available online for this figure.

 © 2023 The Authors
