# Supplementary figures and images for: The ribosome‐associated chaperone Zuo1 controls translation upon TORC1 inhibition
Source: EMBO J. 2023 Nov 20;42(24):e113240. doi: 10.15252/embj.2022113240 (PMC10711665; doi:10.15252/embj.2022113240)

Figure 1C

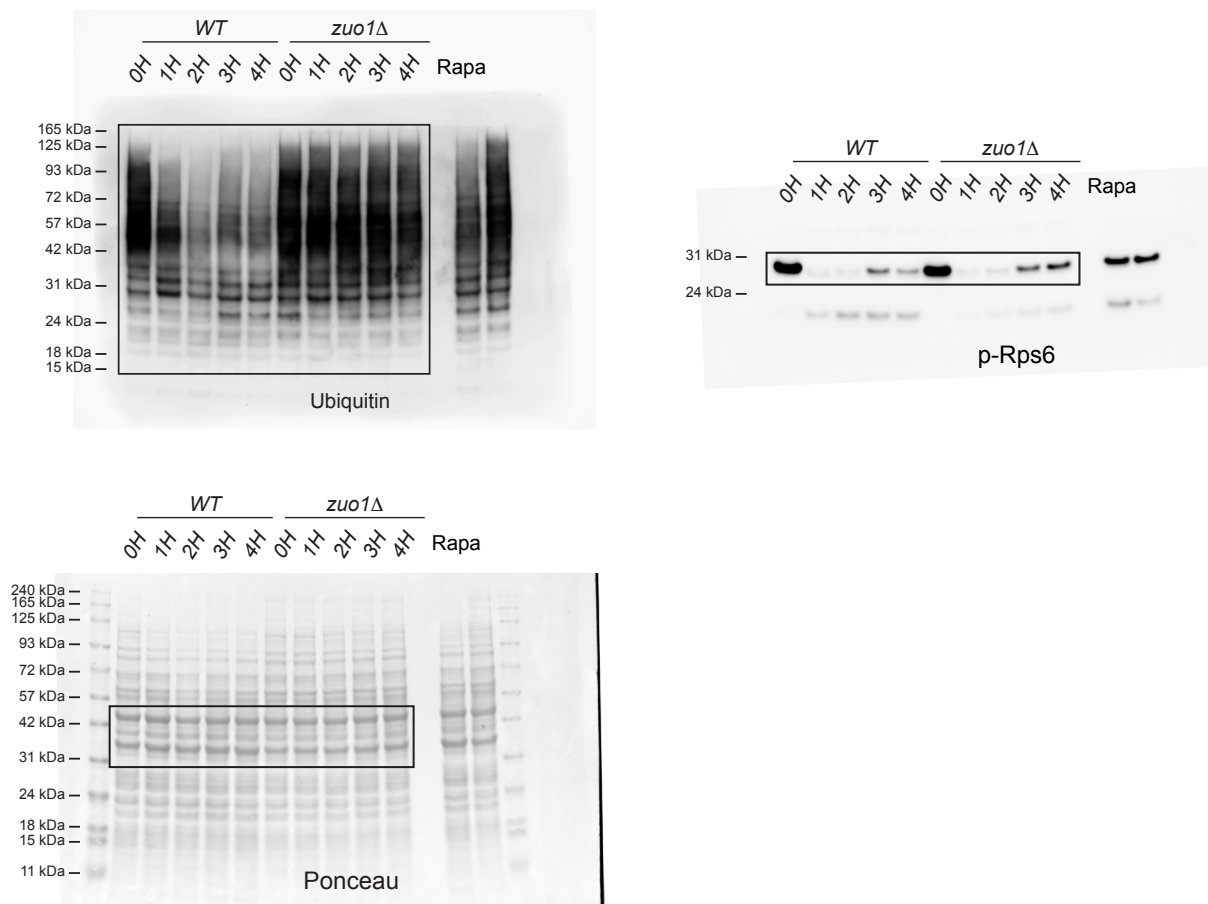

Figure 1D

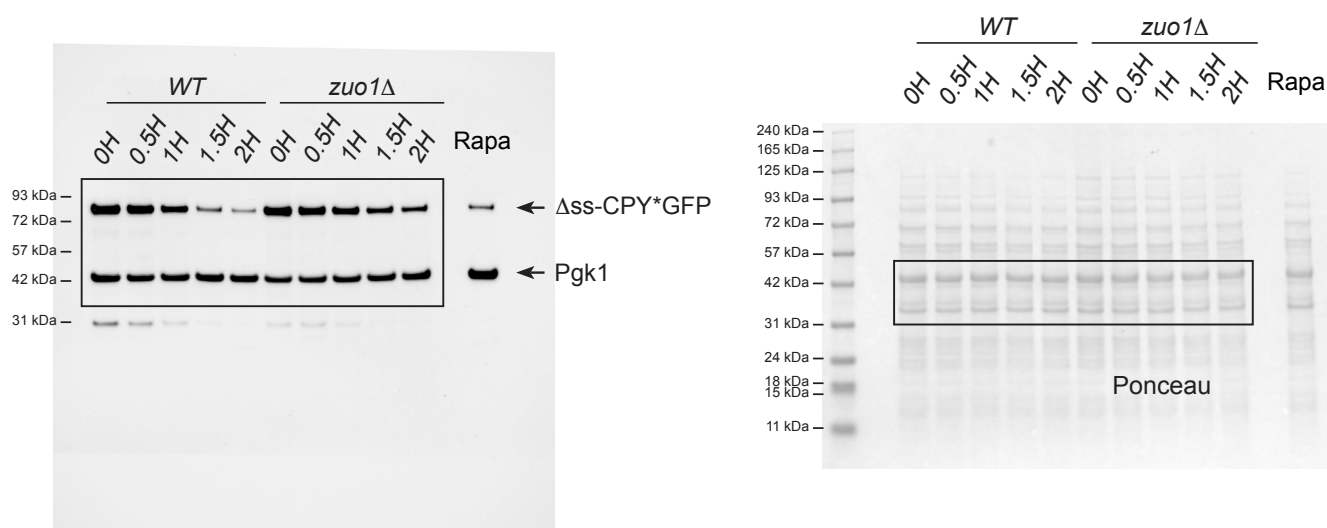

Supplement: Supplementary file 6 — Source Data for Figure 1 [file EMBJ-42-e113240-s004.zip › Figure 1/Raw - Figure 1.pdf]

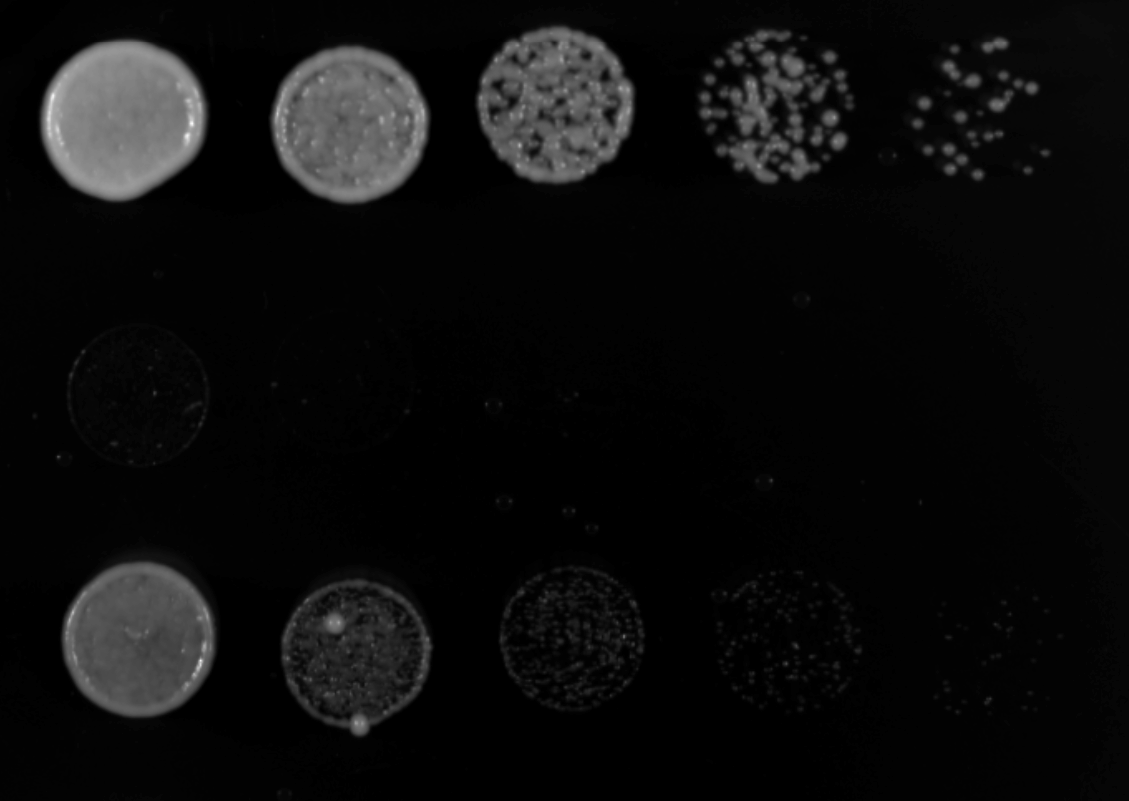

Supplement: Supplementary file 6 — Source Data for Figure 1 [file EMBJ-42-e113240-s004.zip › Figure 1/Figure 1B-Rapamycin.tif]

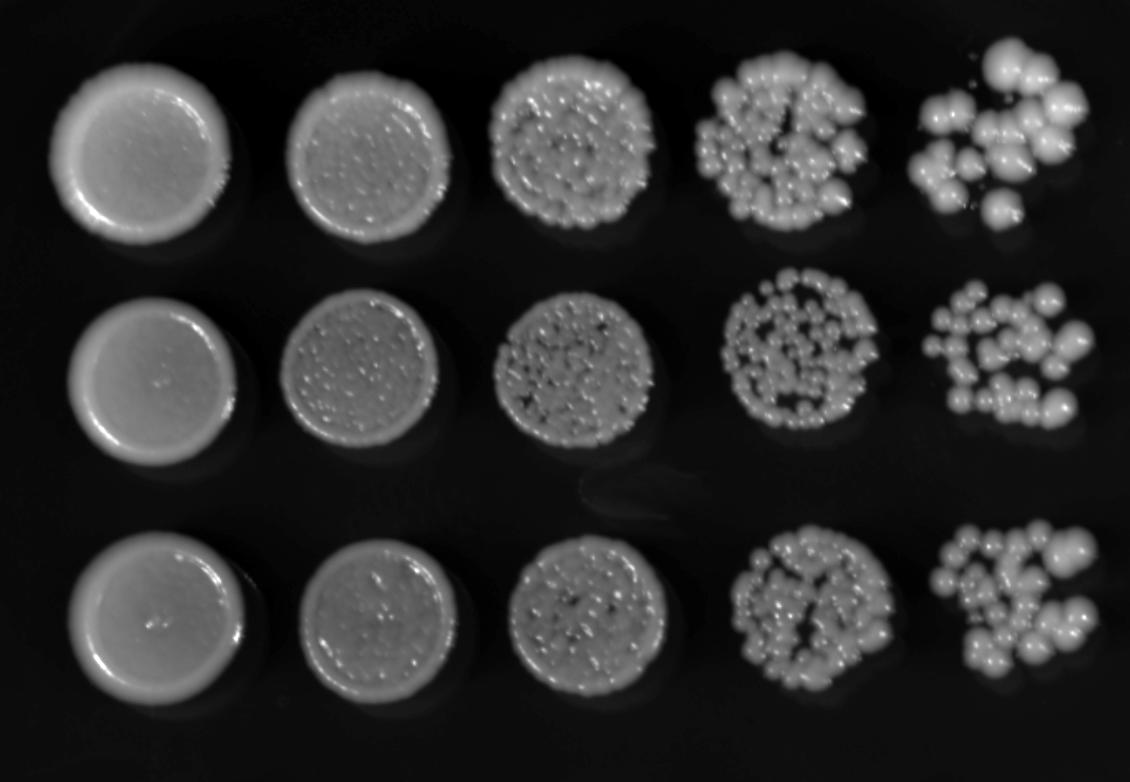

Supplement: Supplementary file 6 — Source Data for Figure 1 [file EMBJ-42-e113240-s004.zip › Figure 1/Figure 1B-YEPD.tif]

Figure 2A

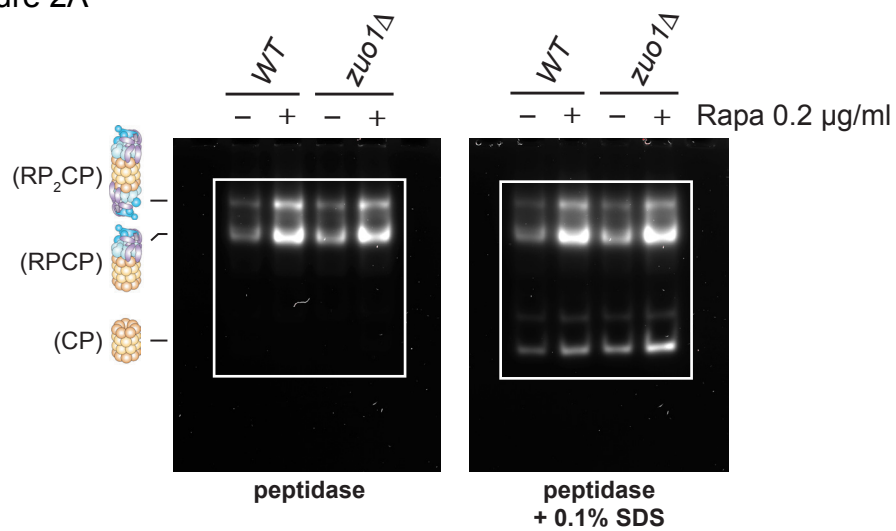

Figure 2B

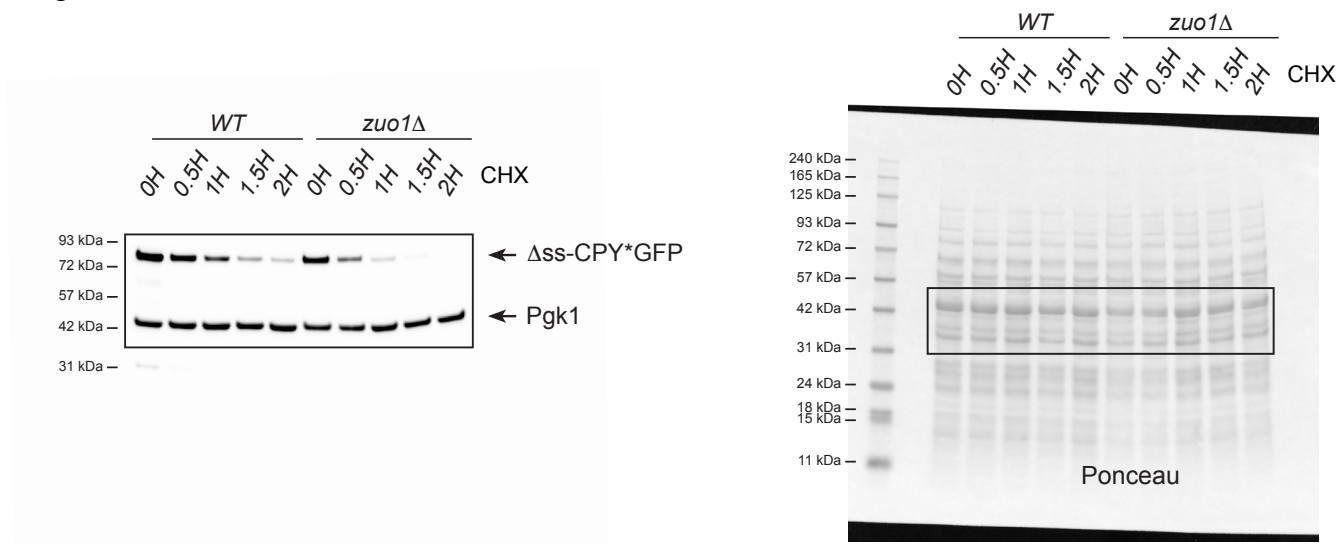

Supplement: Supplementary file 7 — Source Data for Figure 2 [file EMBJ-42-e113240-s008.zip › Figure 2/Raw - Figure 2.pdf]

Figure 3A

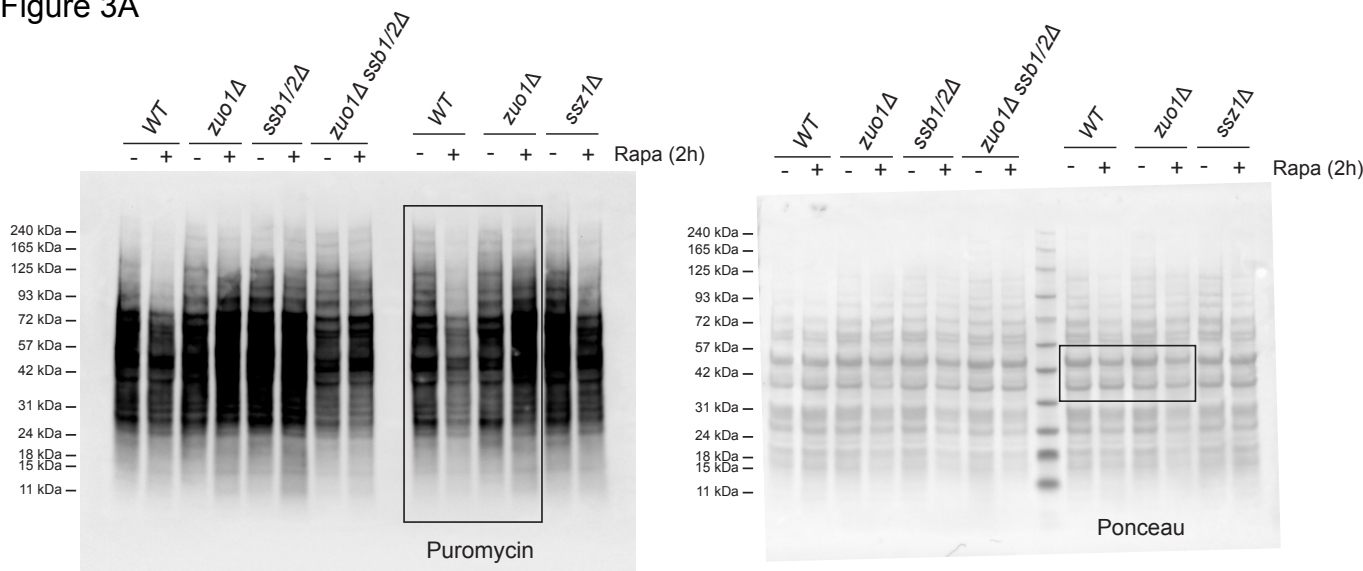

Figure 3B

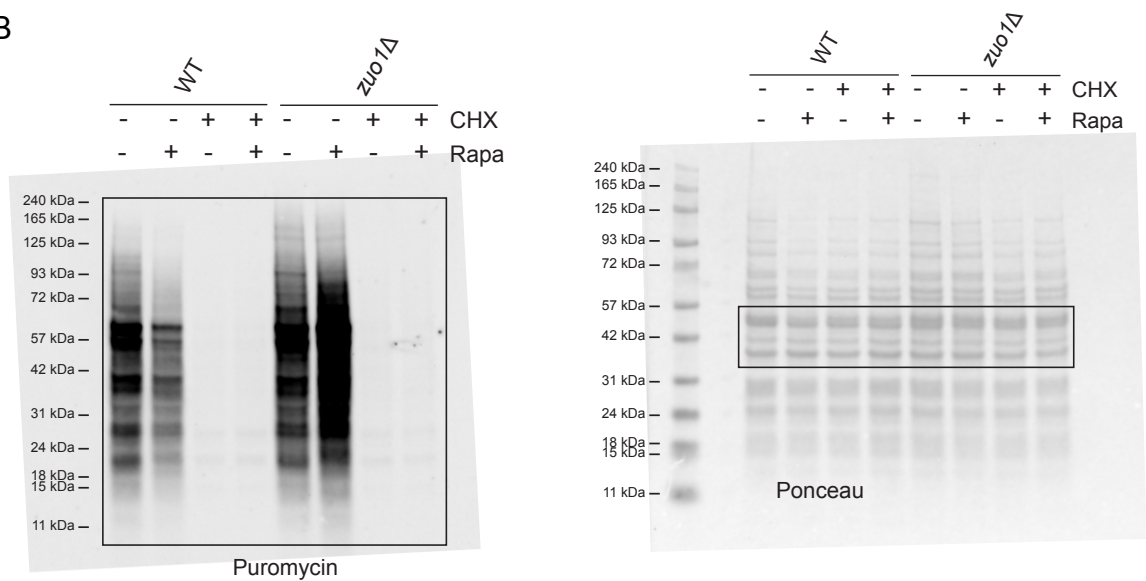

Figure 3C

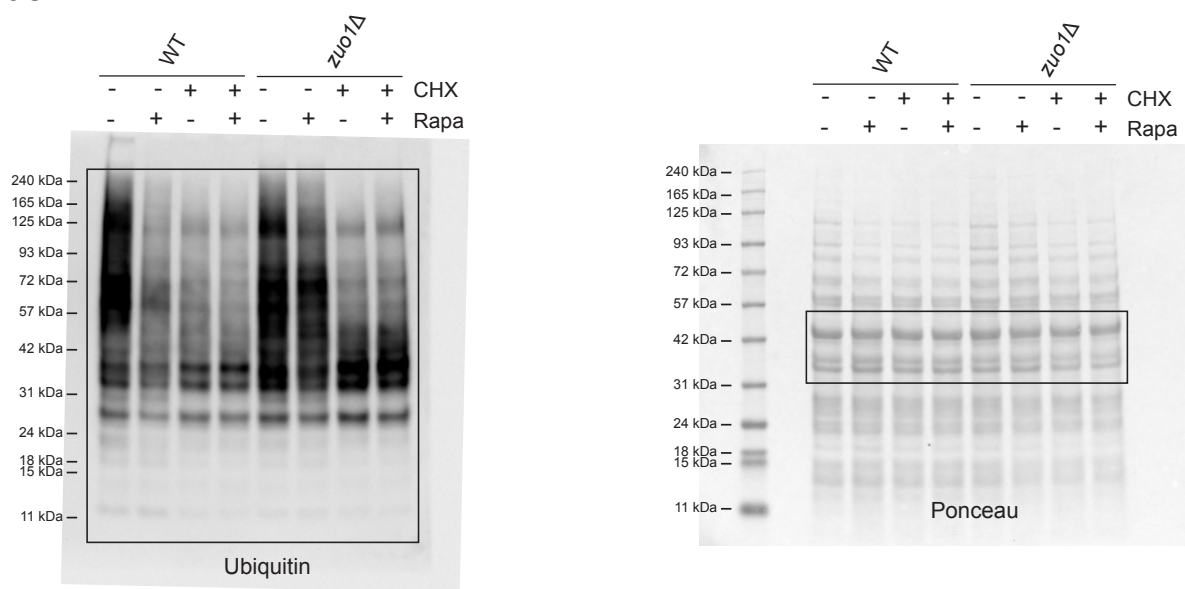

Figure 3D

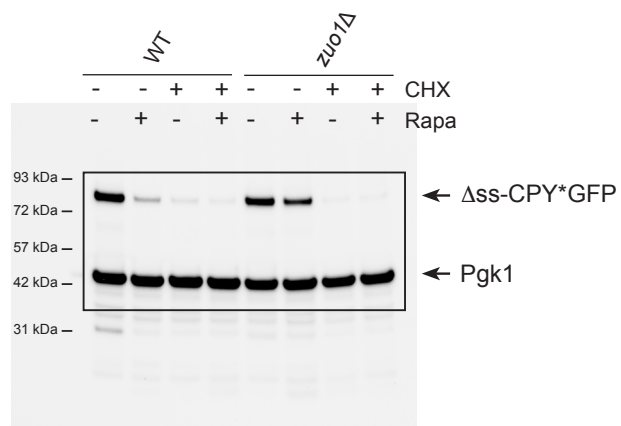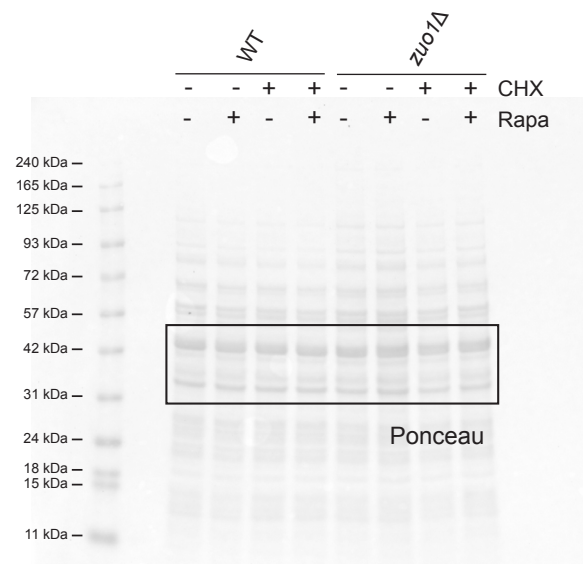

Supplement: Supplementary file 8 — Source Data for Figure 3 [file EMBJ-42-e113240-s012.zip › Figure 3/Raw - Figure 3.pdf]

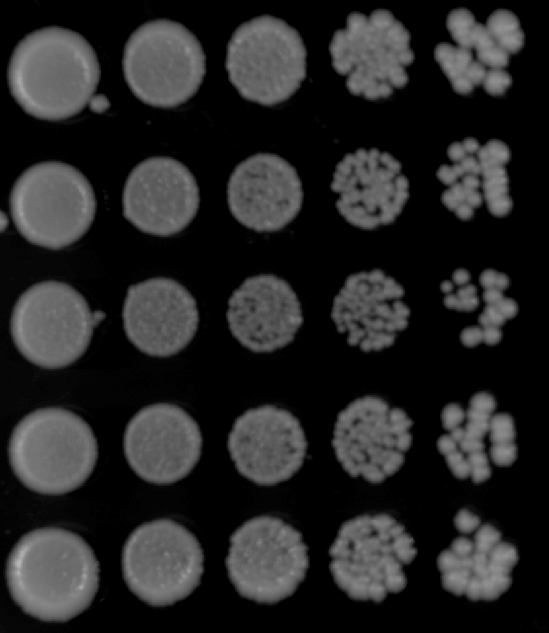

Supplement: Supplementary file 9 — Source Data for Figure 4 [file EMBJ-42-e113240-s002.zip › Figure 4/Figure 4B-YEPD.tif]

Figure 4C

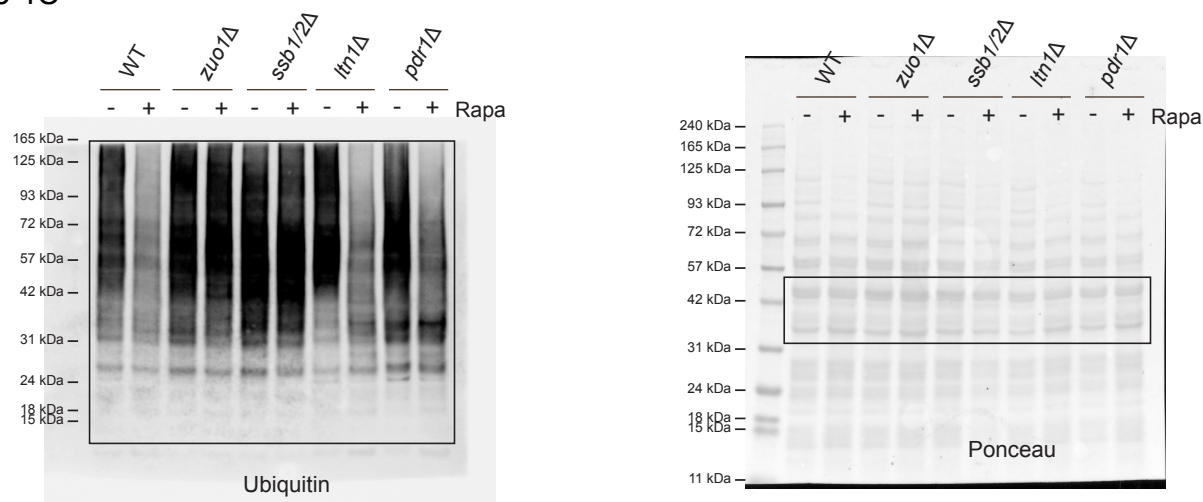

Figure 4D

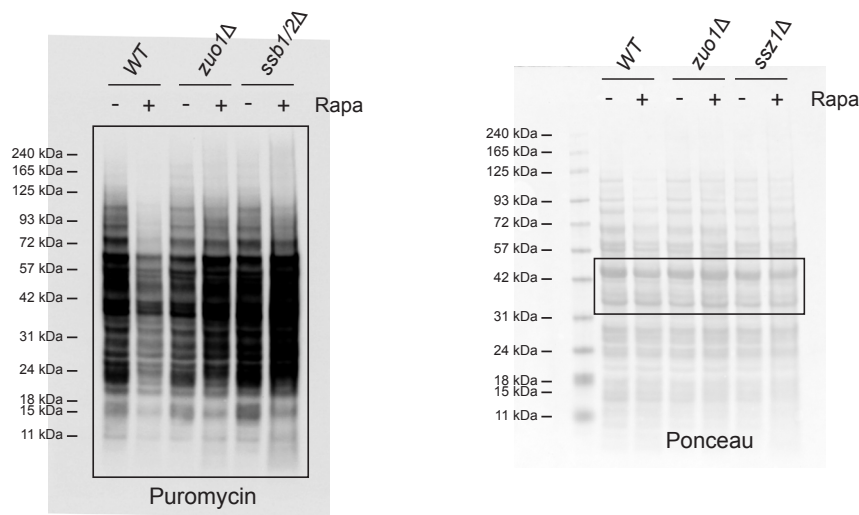

Figure 4E

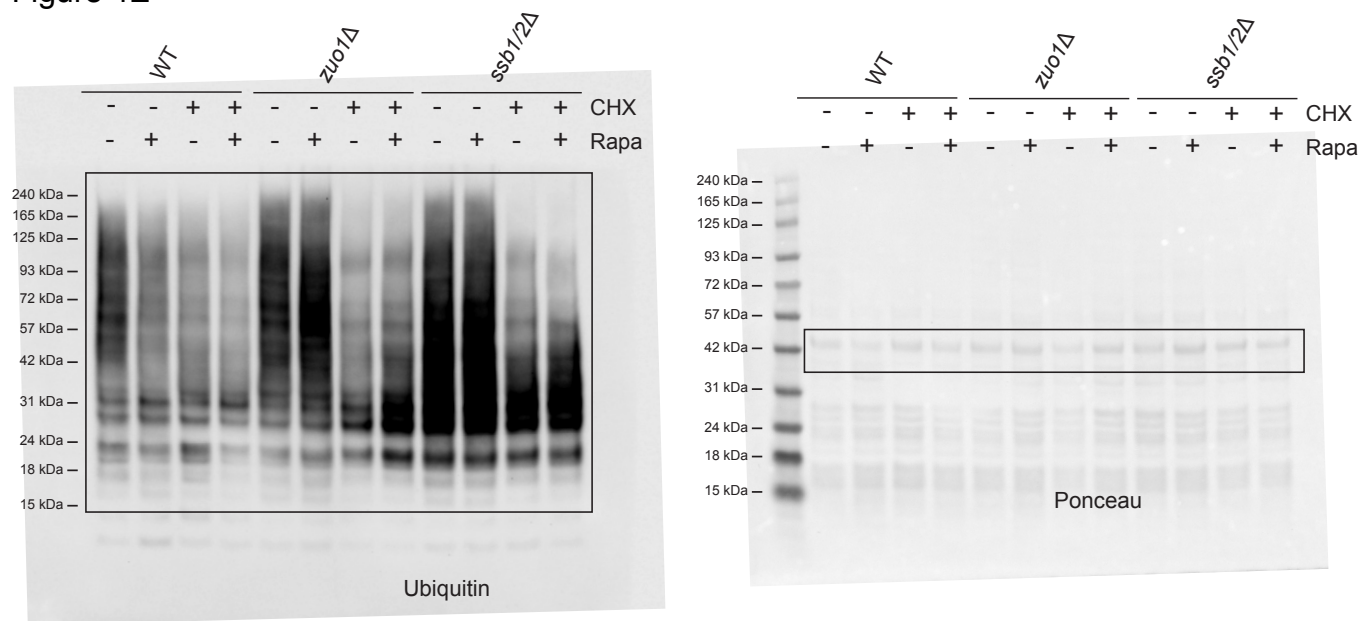

Supplement: Supplementary file 9 — Source Data for Figure 4 [file EMBJ-42-e113240-s002.zip › Figure 4/Raw - Figure 4.pdf]

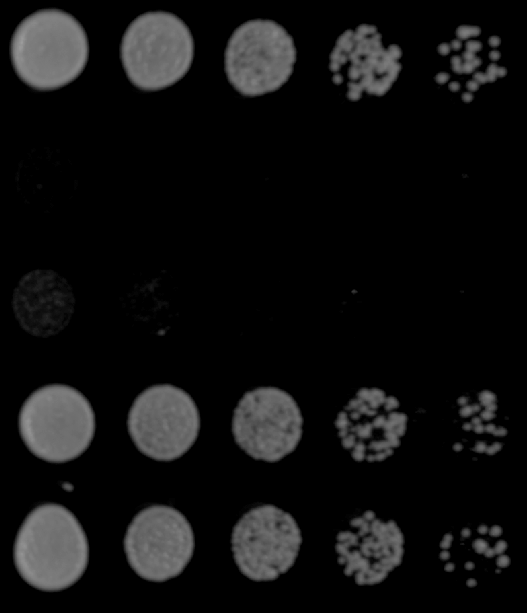

Supplement: Supplementary file 9 — Source Data for Figure 4 [file EMBJ-42-e113240-s002.zip › Figure 4/Figure 4B-Rapamycin.tif]

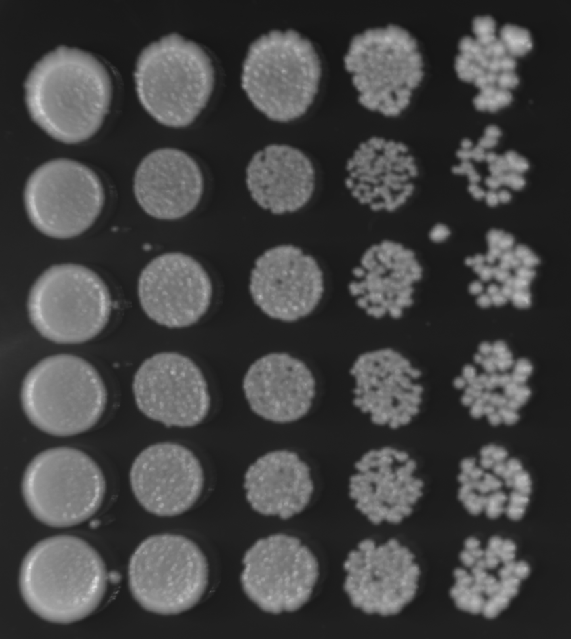

Supplement: Supplementary file 10 — Source Data for Figure 5 [file EMBJ-42-e113240-s010.zip › Figure 5/Figure 5E-YEPD.tif]

Figure 5B

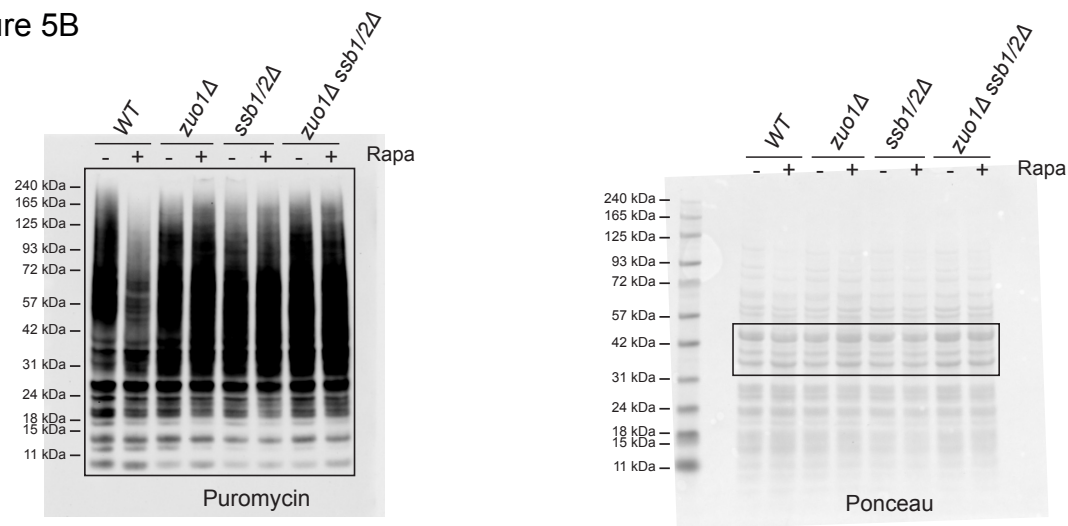

Figure 5C

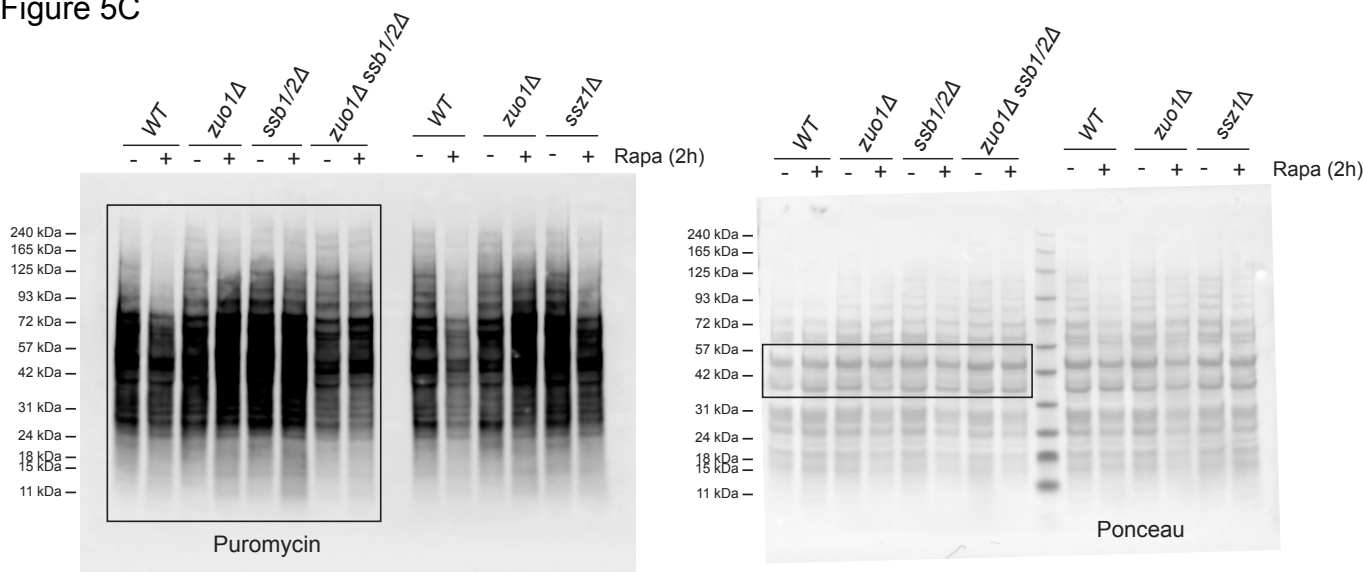

Figure 5F

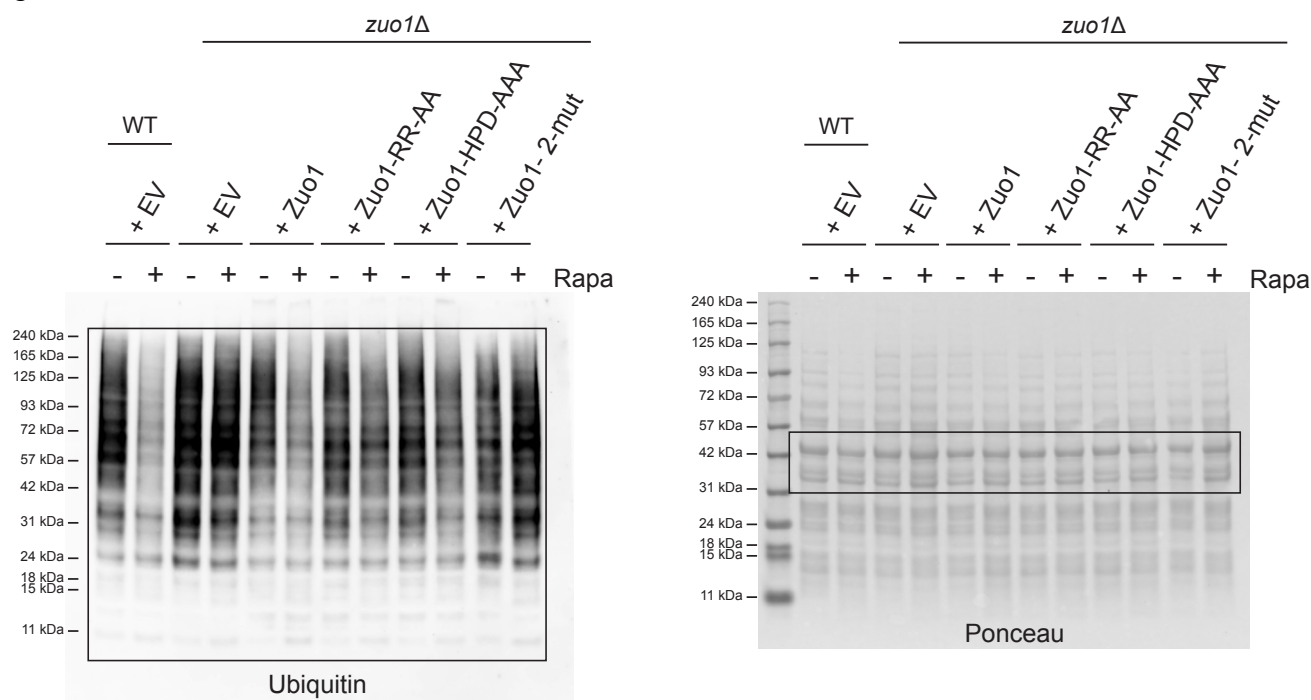

Figure 5G

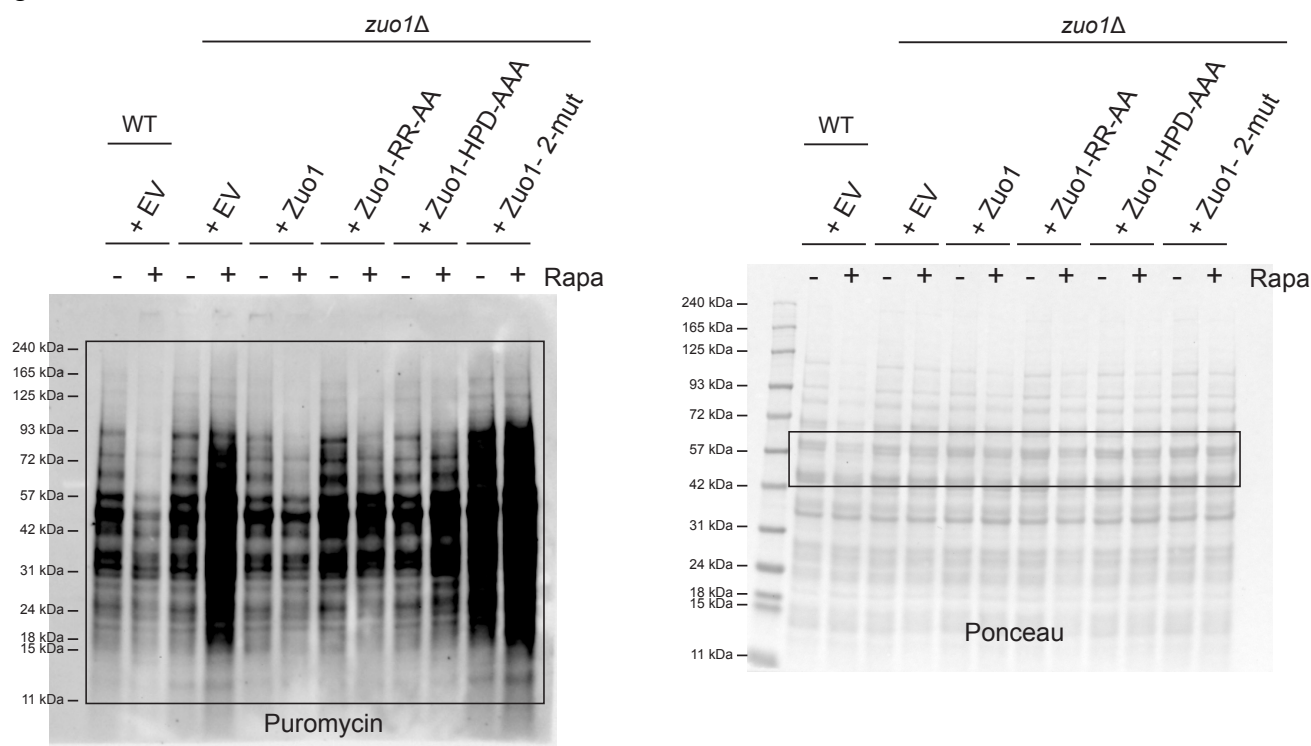

Supplement: Supplementary file 10 — Source Data for Figure 5 [file EMBJ-42-e113240-s010.zip › Figure 5/Raw - Figure 5.pdf]

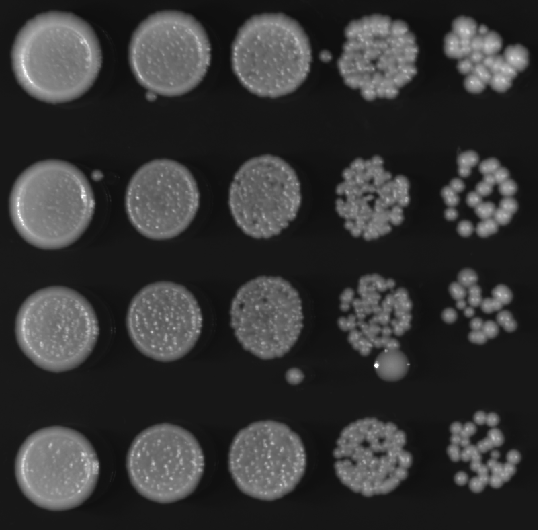

Supplement: Supplementary file 10 — Source Data for Figure 5 [file EMBJ-42-e113240-s010.zip › Figure 5/Figure 5A-YEPD.tif]

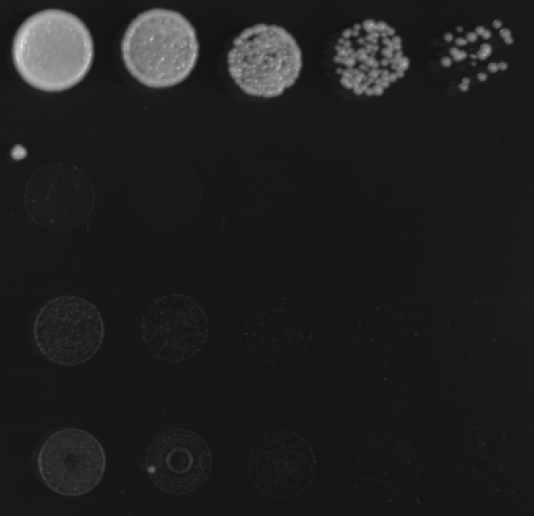

Supplement: Supplementary file 10 — Source Data for Figure 5 [file EMBJ-42-e113240-s010.zip › Figure 5/Figure 5A-Rapamycin.tif]

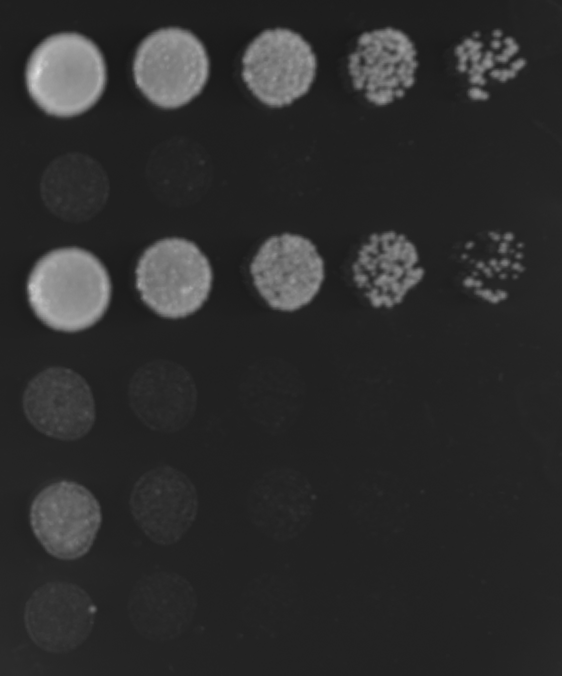

Supplement: Supplementary file 10 — Source Data for Figure 5 [file EMBJ-42-e113240-s010.zip › Figure 5/Figure 5E-Rapamycin.tif]

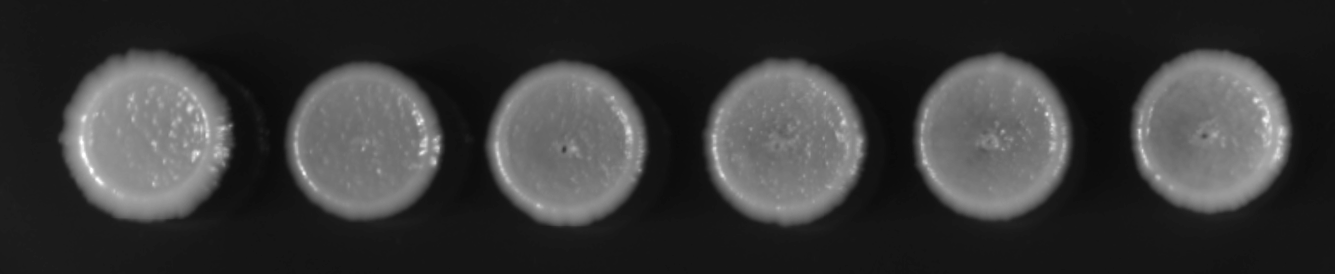

Supplement: Supplementary file 11 — Source Data for Figure 6 [file EMBJ-42-e113240-s014.zip › Figure 6/Figure 6C-More bound - Set4-YEPD.tif]

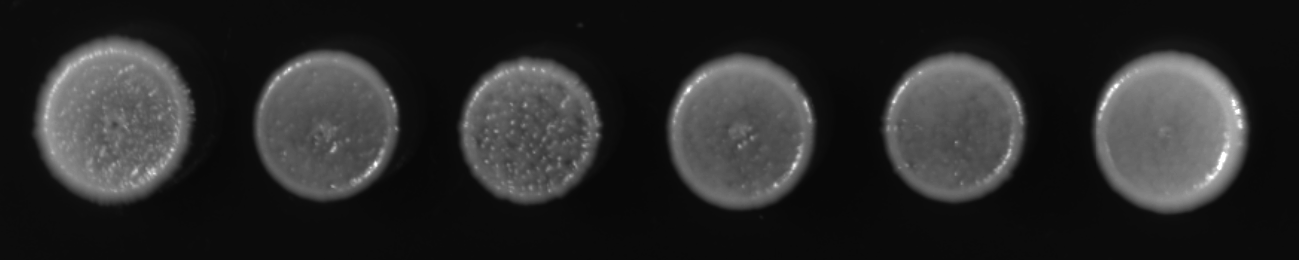

Supplement: Supplementary file 11 — Source Data for Figure 6 [file EMBJ-42-e113240-s014.zip › Figure 6/Figure 6C-More bound - Set5-YEPD.tif]

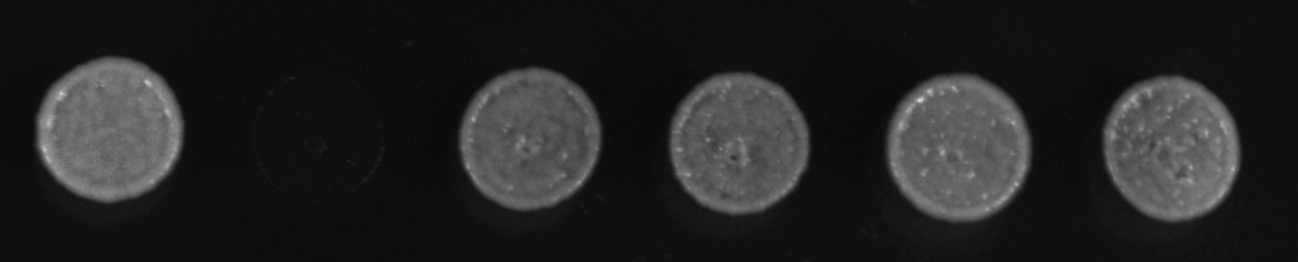

Supplement: Supplementary file 11 — Source Data for Figure 6 [file EMBJ-42-e113240-s014.zip › Figure 6/Figure 6C-Less bound-Rapamycin.tif]

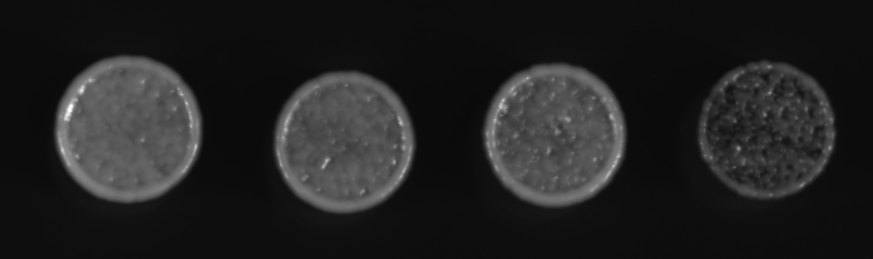

Supplement: Supplementary file 11 — Source Data for Figure 6 [file EMBJ-42-e113240-s014.zip › Figure 6/Figure 6C-More bound - Set8-Rapamycin.tif]

Figure 6A

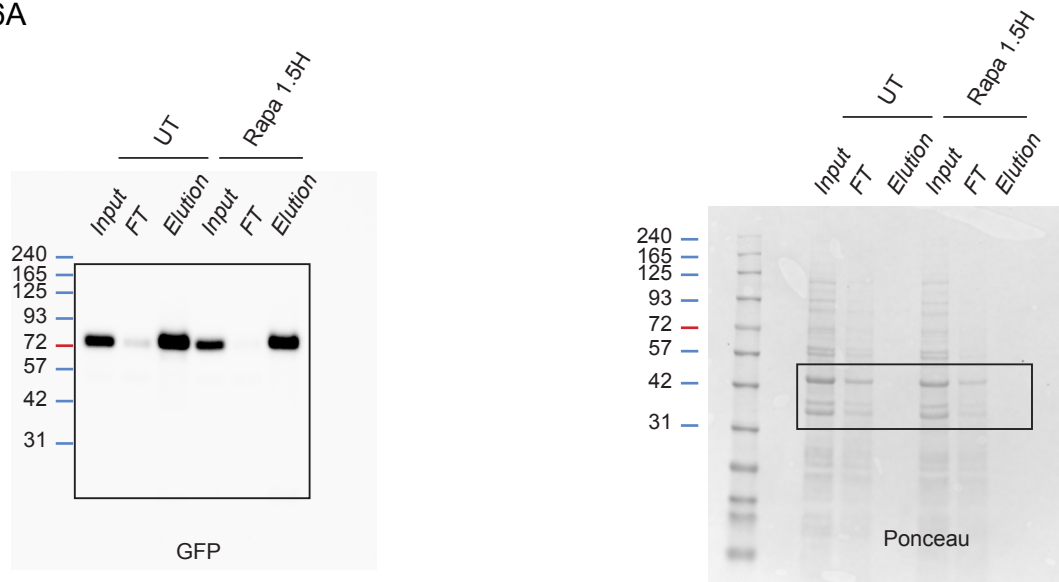

Figure 6D

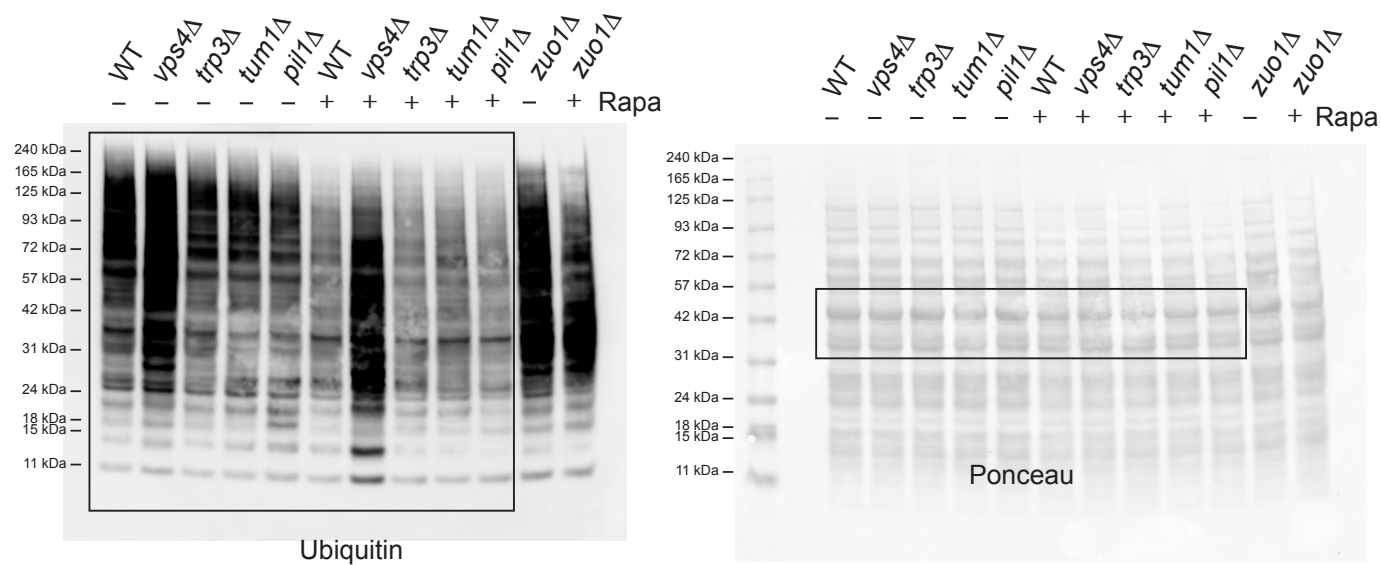

Figure 6E

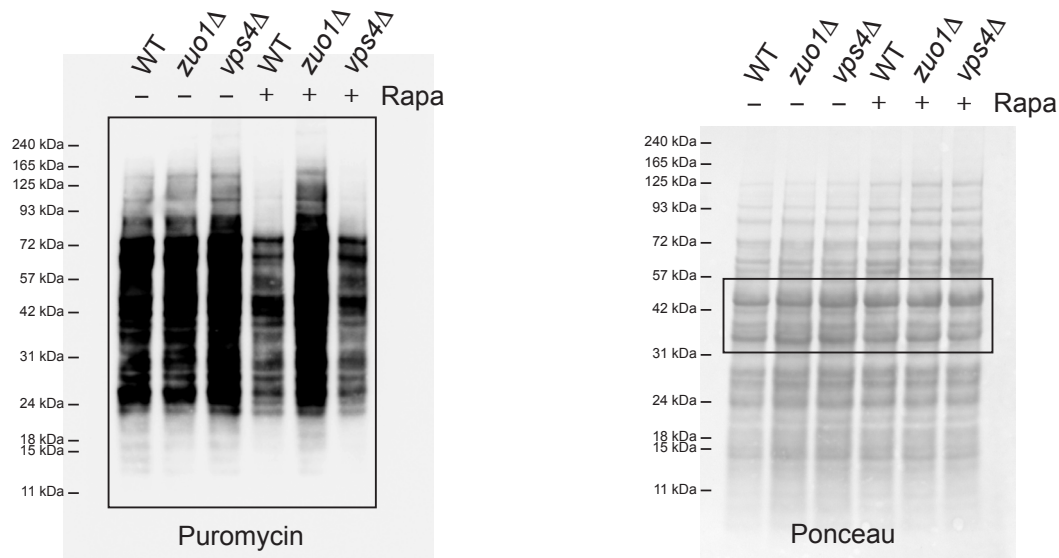

Supplement: Supplementary file 11 — Source Data for Figure 6 [file EMBJ-42-e113240-s014.zip › Figure 6/Raw - Figure 6.pdf]

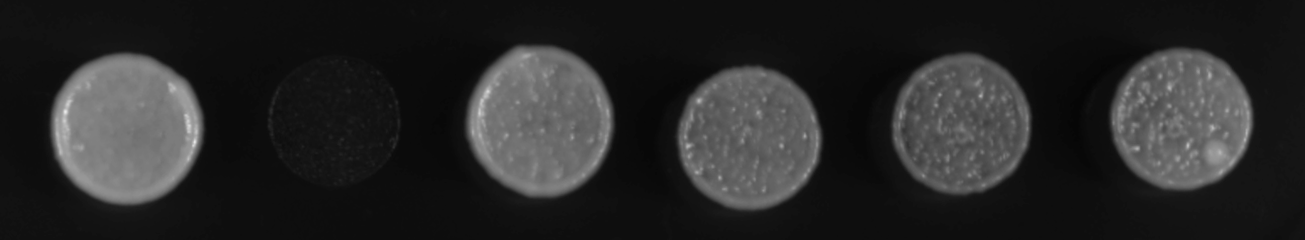

Supplement: Supplementary file 11 — Source Data for Figure 6 [file EMBJ-42-e113240-s014.zip › Figure 6/Figure 6C-More bound - Set2-Rapamycin.tif]

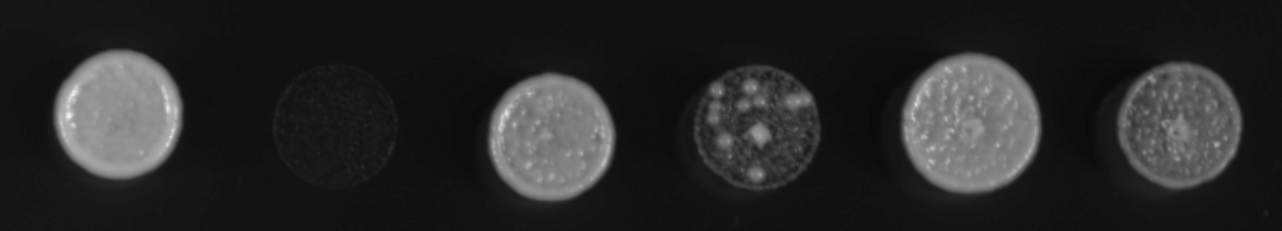

Supplement: Supplementary file 11 — Source Data for Figure 6 [file EMBJ-42-e113240-s014.zip › Figure 6/Figure 6C-More bound - Set6-Rapamycin.tif]

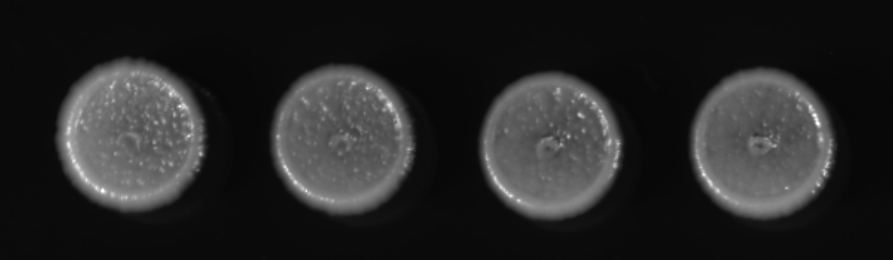

Supplement: Supplementary file 11 — Source Data for Figure 6 [file EMBJ-42-e113240-s014.zip › Figure 6/Figure 6C-More bound - Set8-YEPD.tif]

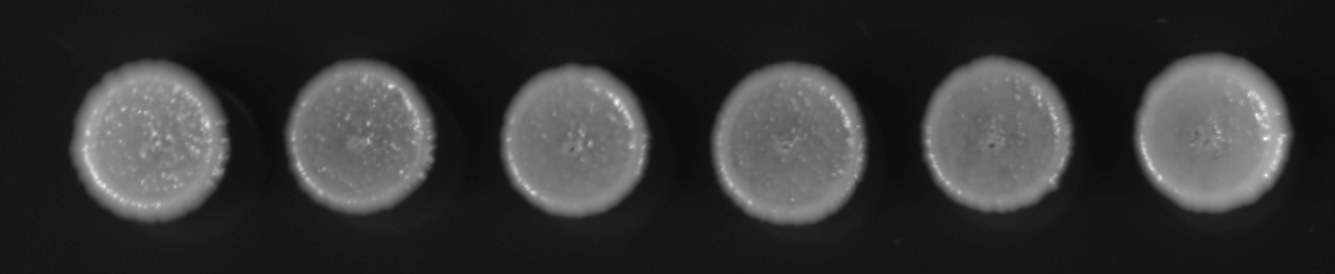

Supplement: Supplementary file 11 — Source Data for Figure 6 [file EMBJ-42-e113240-s014.zip › Figure 6/Figure 6C-More bound - Set3-YEPD.tif]

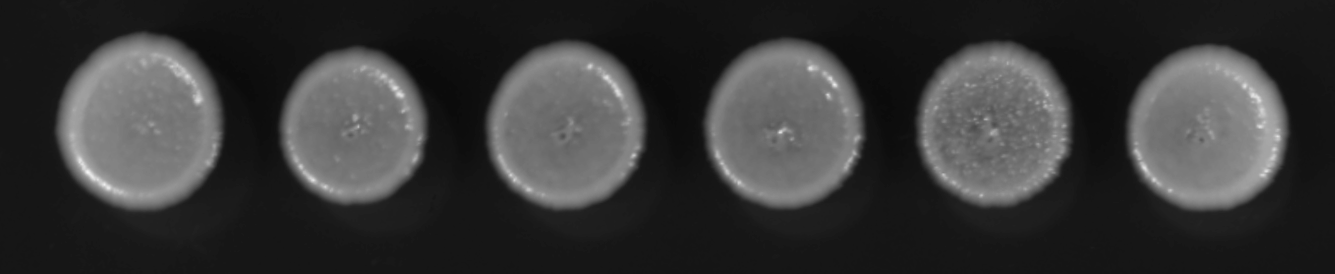

Supplement: Supplementary file 11 — Source Data for Figure 6 [file EMBJ-42-e113240-s014.zip › Figure 6/Figure 6C-More bound - Set2-YEPD.tif]

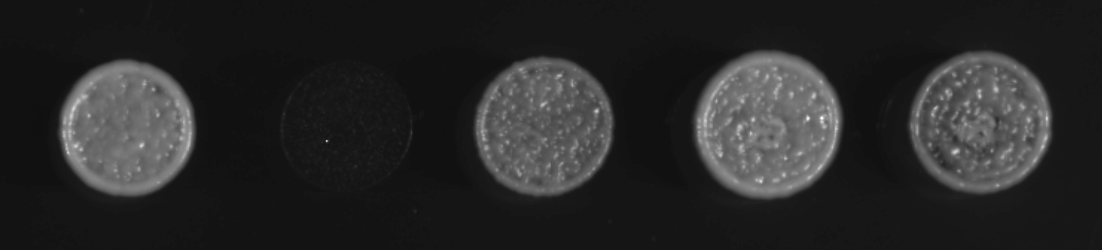

Supplement: Supplementary file 11 — Source Data for Figure 6 [file EMBJ-42-e113240-s014.zip › Figure 6/Figure 6C-More bound - Set1-Rapamycin.tif]

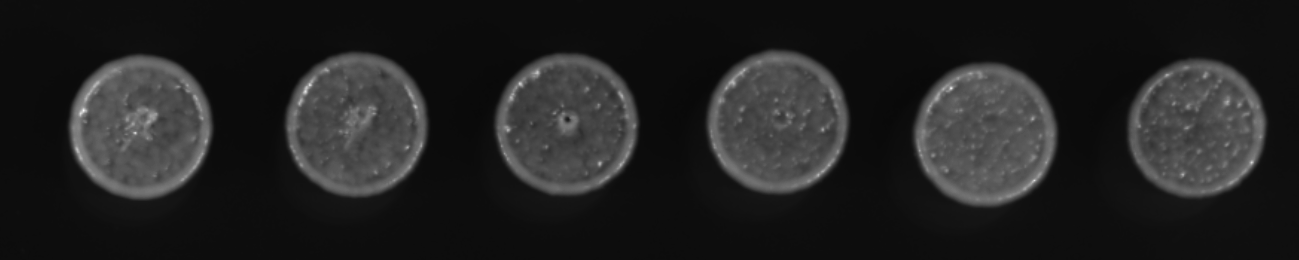

Supplement: Supplementary file 11 — Source Data for Figure 6 [file EMBJ-42-e113240-s014.zip › Figure 6/Figure 6C-More bound - Set5-Rapamycin.tif]

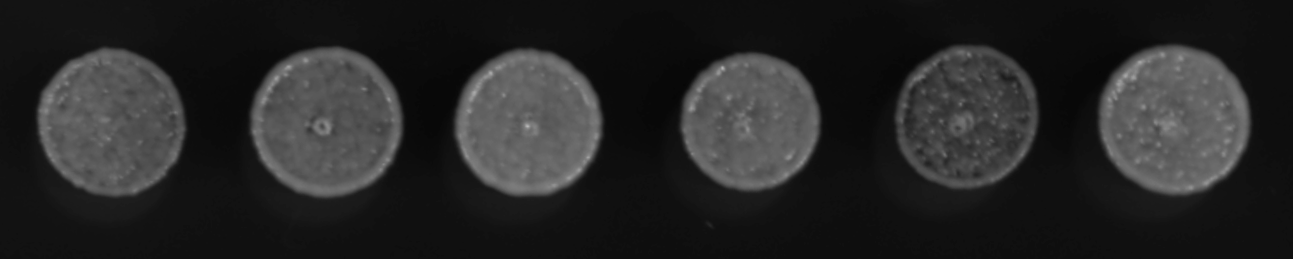

Supplement: Supplementary file 11 — Source Data for Figure 6 [file EMBJ-42-e113240-s014.zip › Figure 6/Figure 6C-More bound - Set4-Rapamycin.tif]

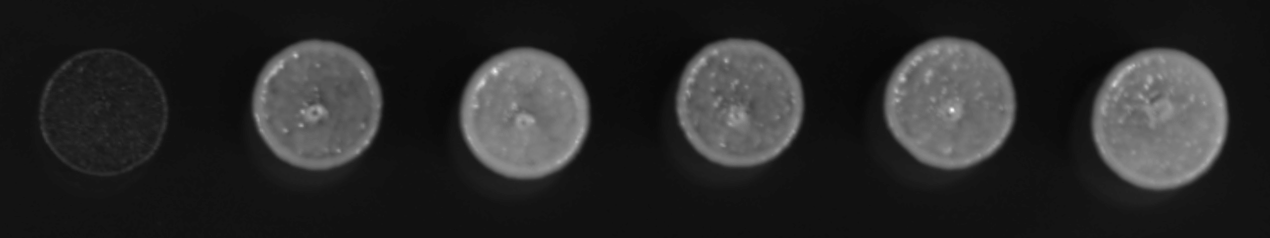

Supplement: Supplementary file 11 — Source Data for Figure 6 [file EMBJ-42-e113240-s014.zip › Figure 6/Figure 6C-More bound - Set7-Rapamycin.tif]

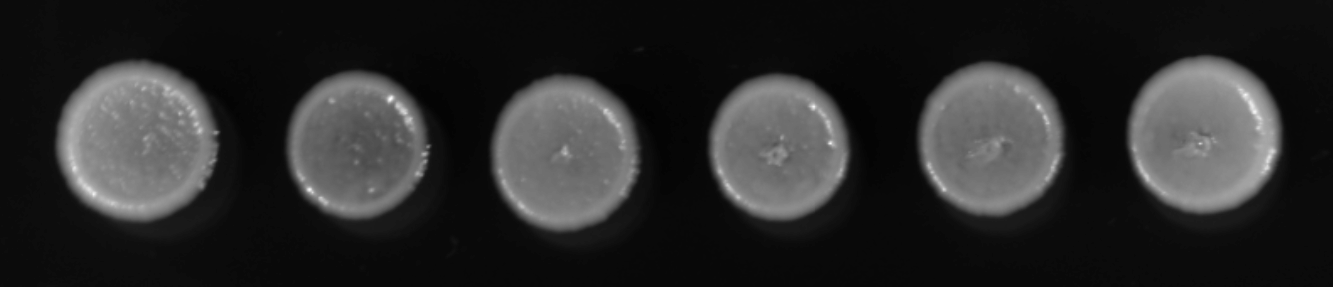

Supplement: Supplementary file 11 — Source Data for Figure 6 [file EMBJ-42-e113240-s014.zip › Figure 6/Figure 6C-More bound - Set7-YEPD.tif]

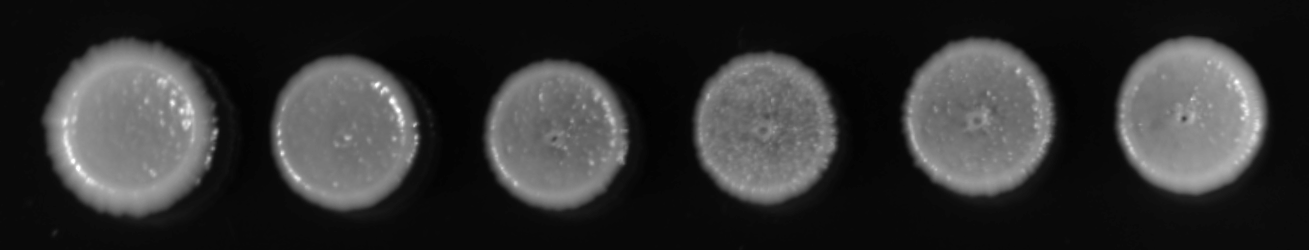

Supplement: Supplementary file 11 — Source Data for Figure 6 [file EMBJ-42-e113240-s014.zip › Figure 6/Figure 6C-More bound - Set6-YEPD.tif]

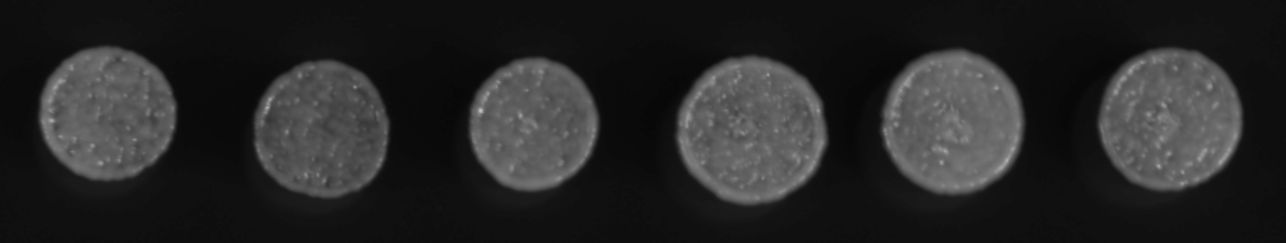

Supplement: Supplementary file 11 — Source Data for Figure 6 [file EMBJ-42-e113240-s014.zip › Figure 6/Figure 6C-More bound - Set3-Rapamycin.tif]

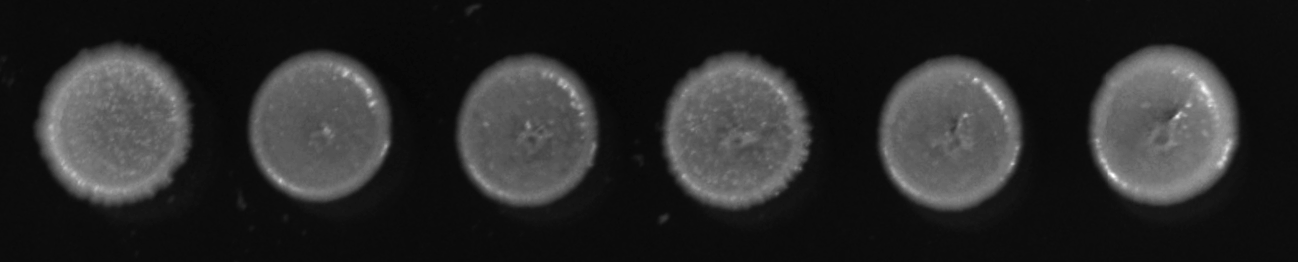

Supplement: Supplementary file 11 — Source Data for Figure 6 [file EMBJ-42-e113240-s014.zip › Figure 6/Figure 6C-Less bound-YEPD.tif]

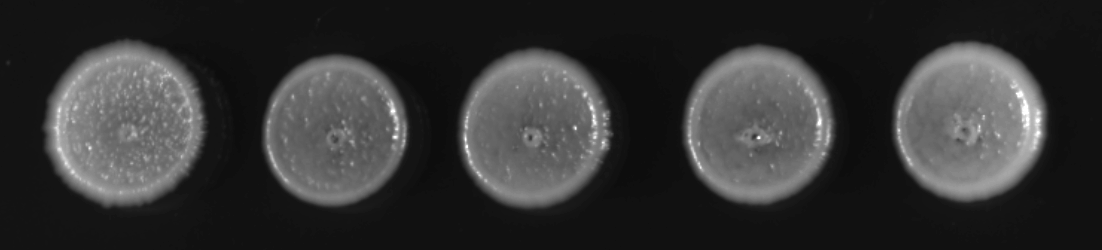

Supplement: Supplementary file 11 — Source Data for Figure 6 [file EMBJ-42-e113240-s014.zip › Figure 6/Figure 6C-More bound - Set1-YEPD.tif]

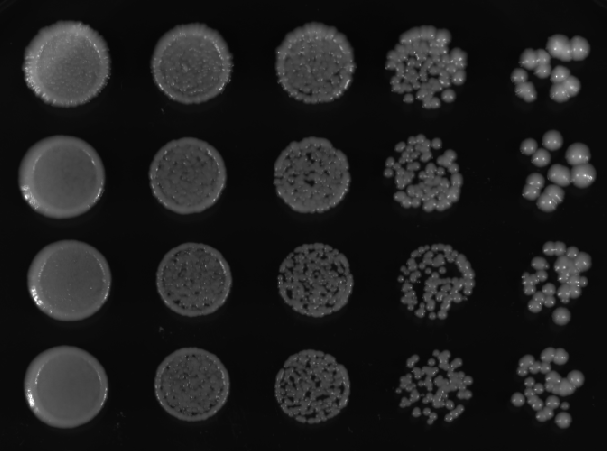

Supplement: Supplementary file 12 — Source Data for Figure 7 [file EMBJ-42-e113240-s009.zip › Figure 7/Figure 7G-YEPD.tif]

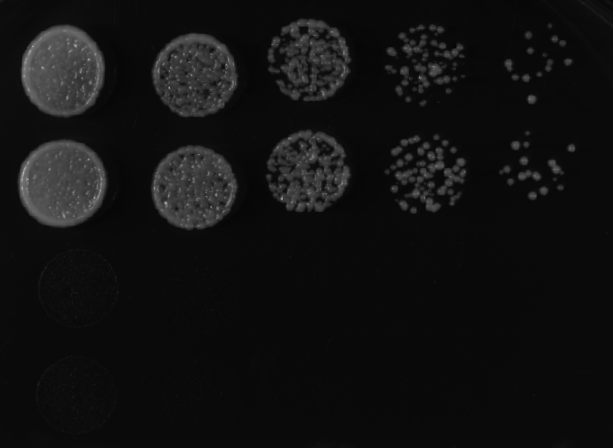

Supplement: Supplementary file 12 — Source Data for Figure 7 [file EMBJ-42-e113240-s009.zip › Figure 7/Figure 7G-Rapamycin.tif]

Figure 7A

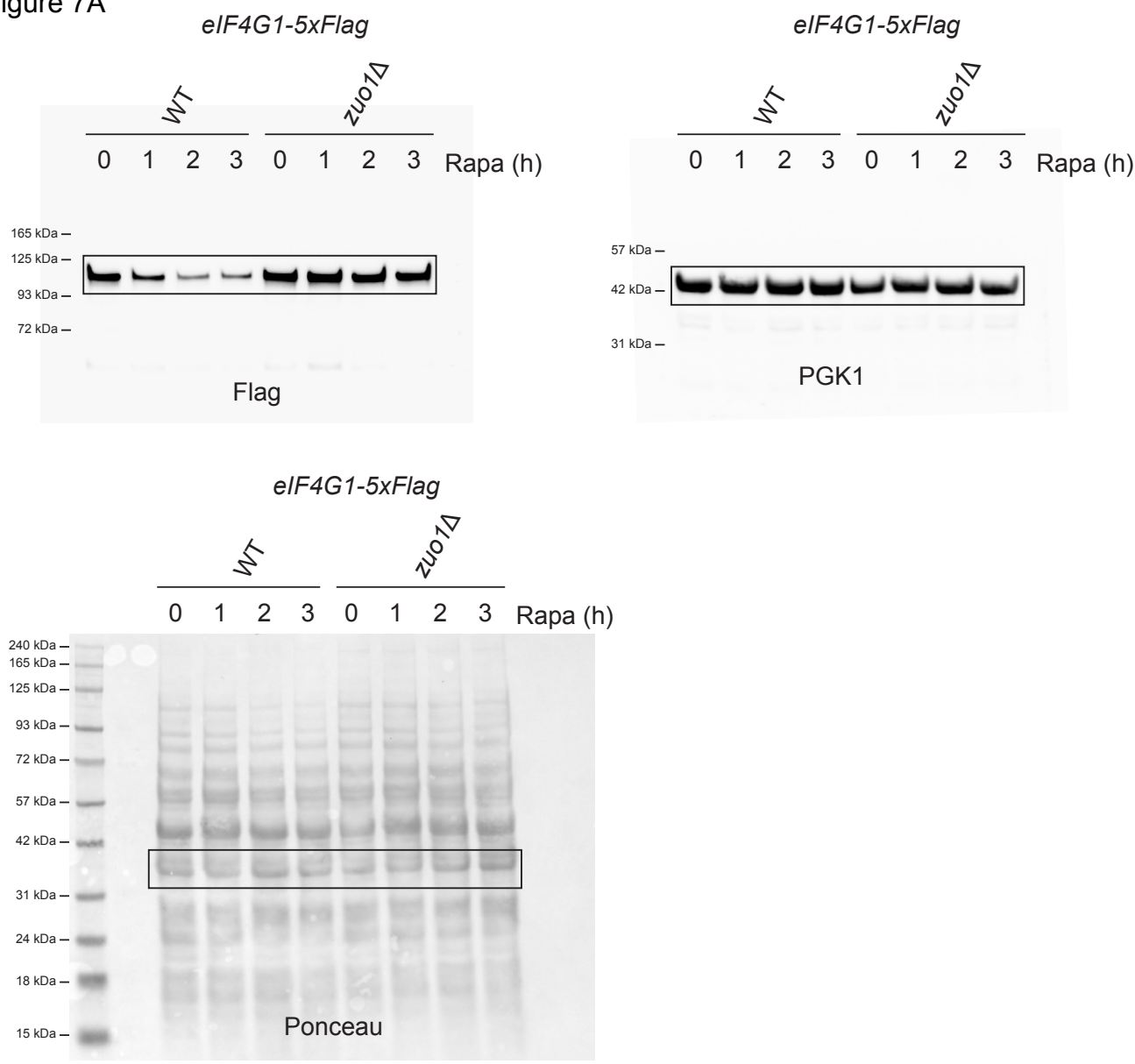

Figure 7C

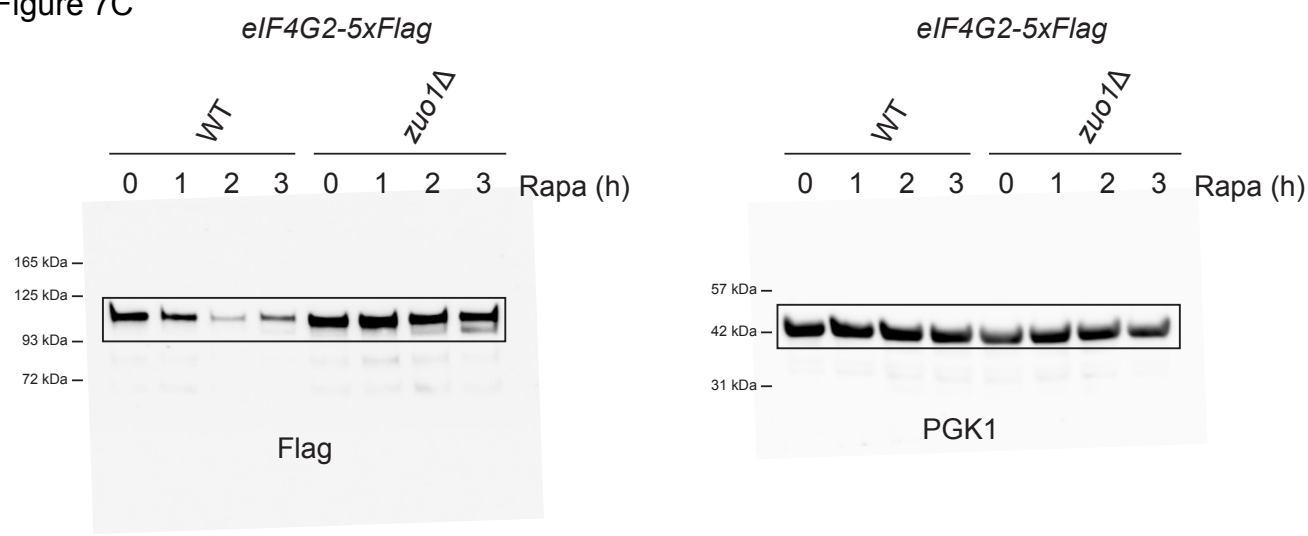

Figure 7C

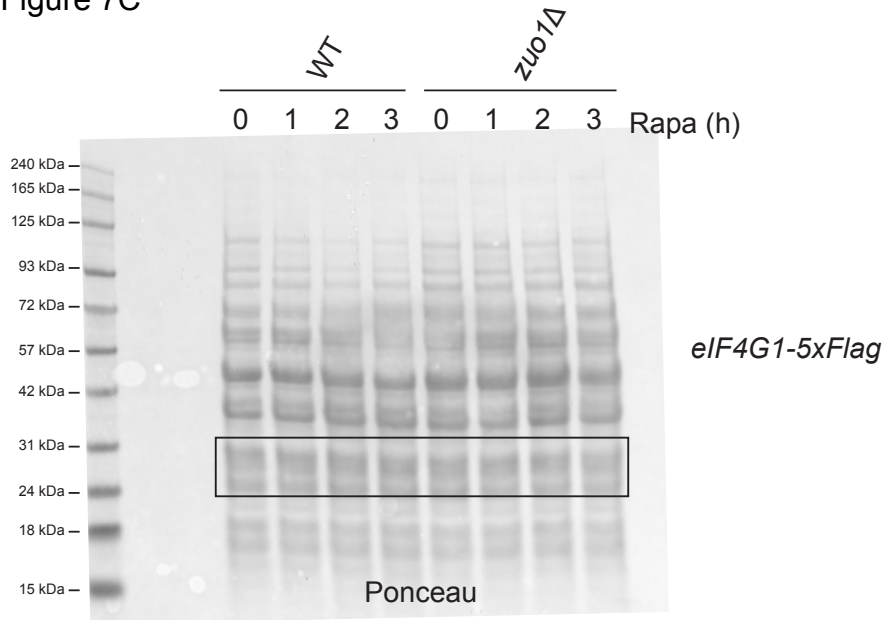

Figure 7H

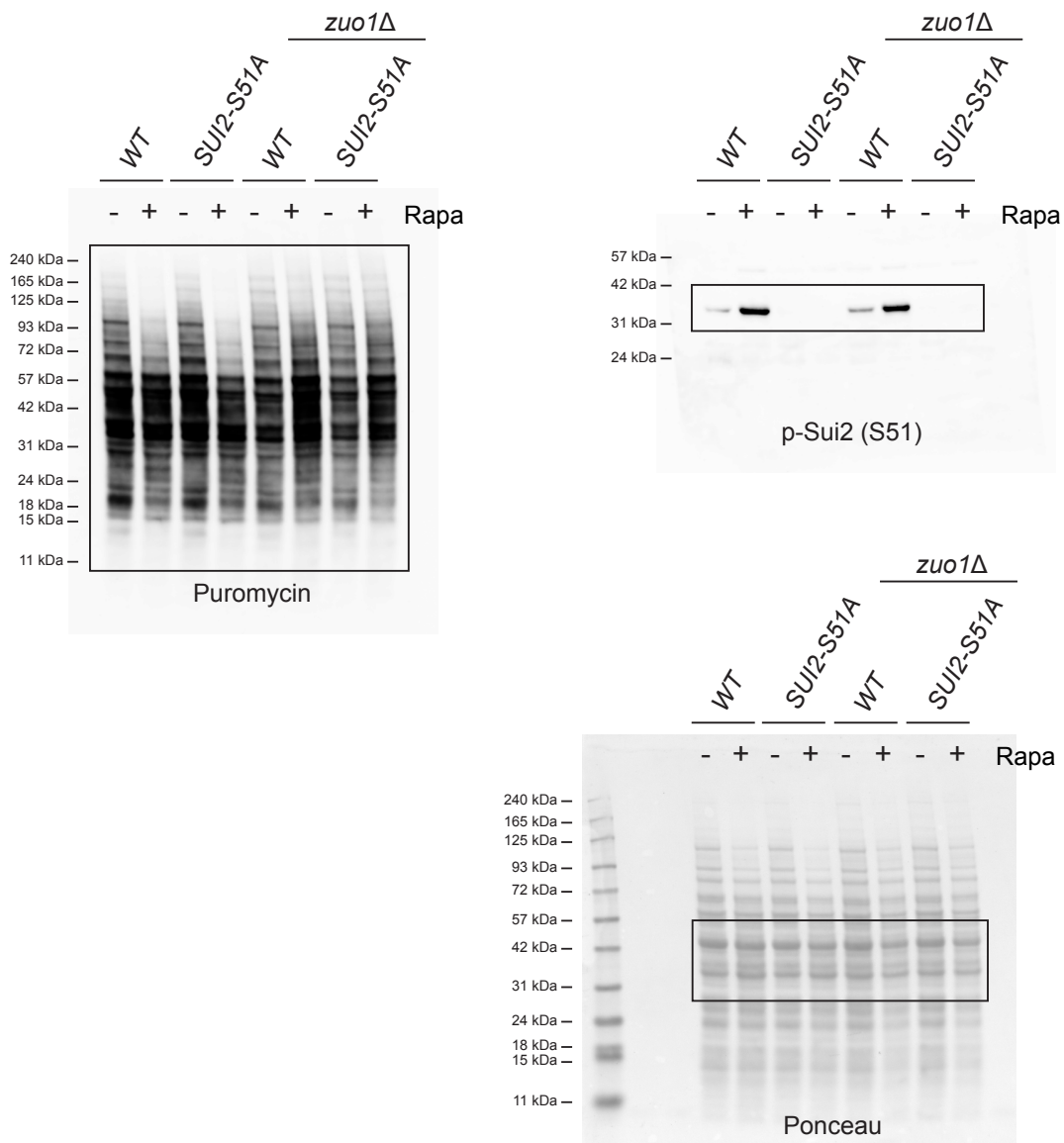

Supplement: Supplementary file 12 — Source Data for Figure 7 [file EMBJ-42-e113240-s009.zip › Figure 7/Raw - Figure 7.pdf]

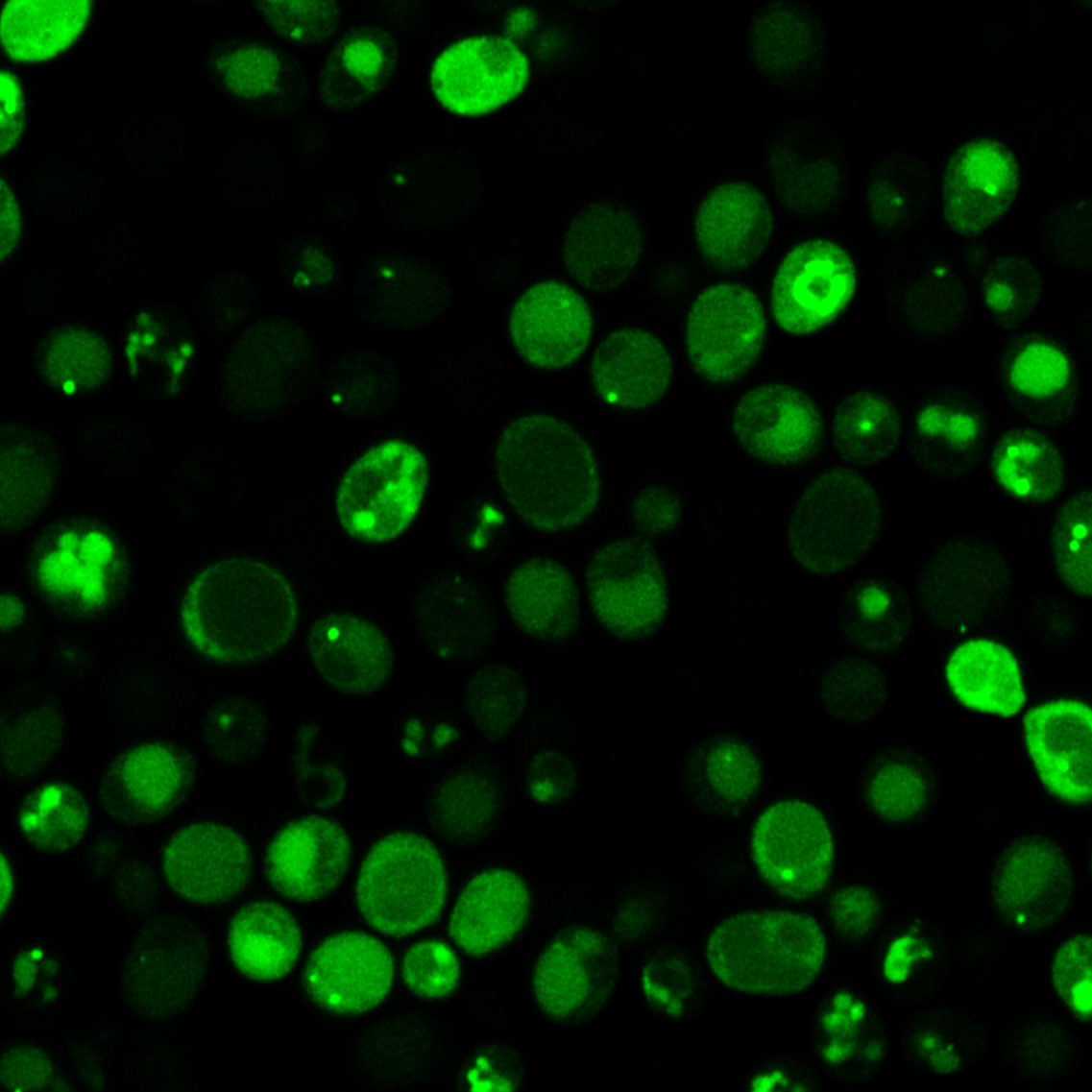

Supplement: Supplementary file 13 — Source Data for Figure 8 [file EMBJ-42-e113240-s003.zip › Figure 8/Figure 8F-Z1D-Rapa.tif]

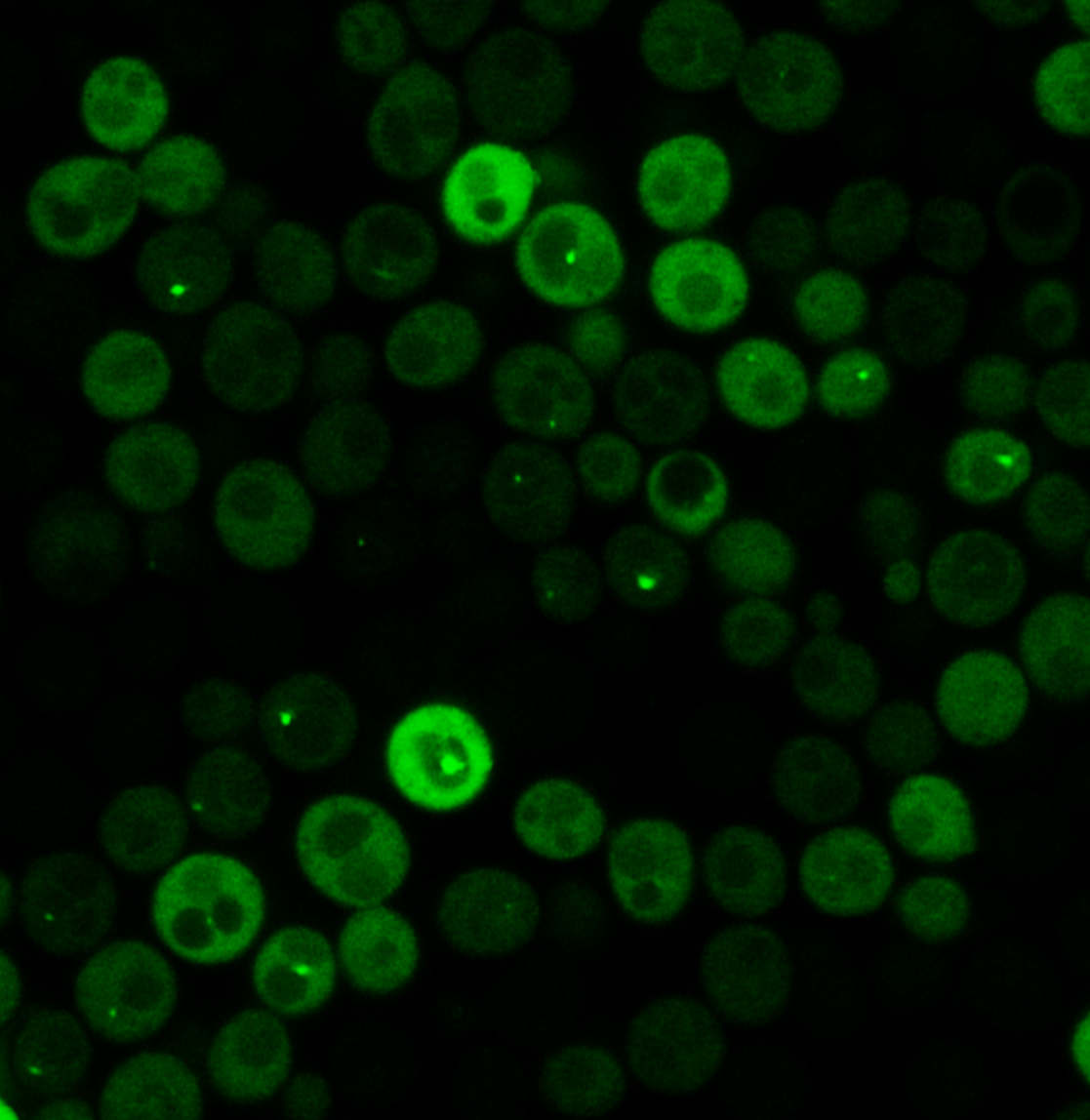

Supplement: Supplementary file 13 — Source Data for Figure 8 [file EMBJ-42-e113240-s003.zip › Figure 8/Figure 8F-WT-UT.tif]

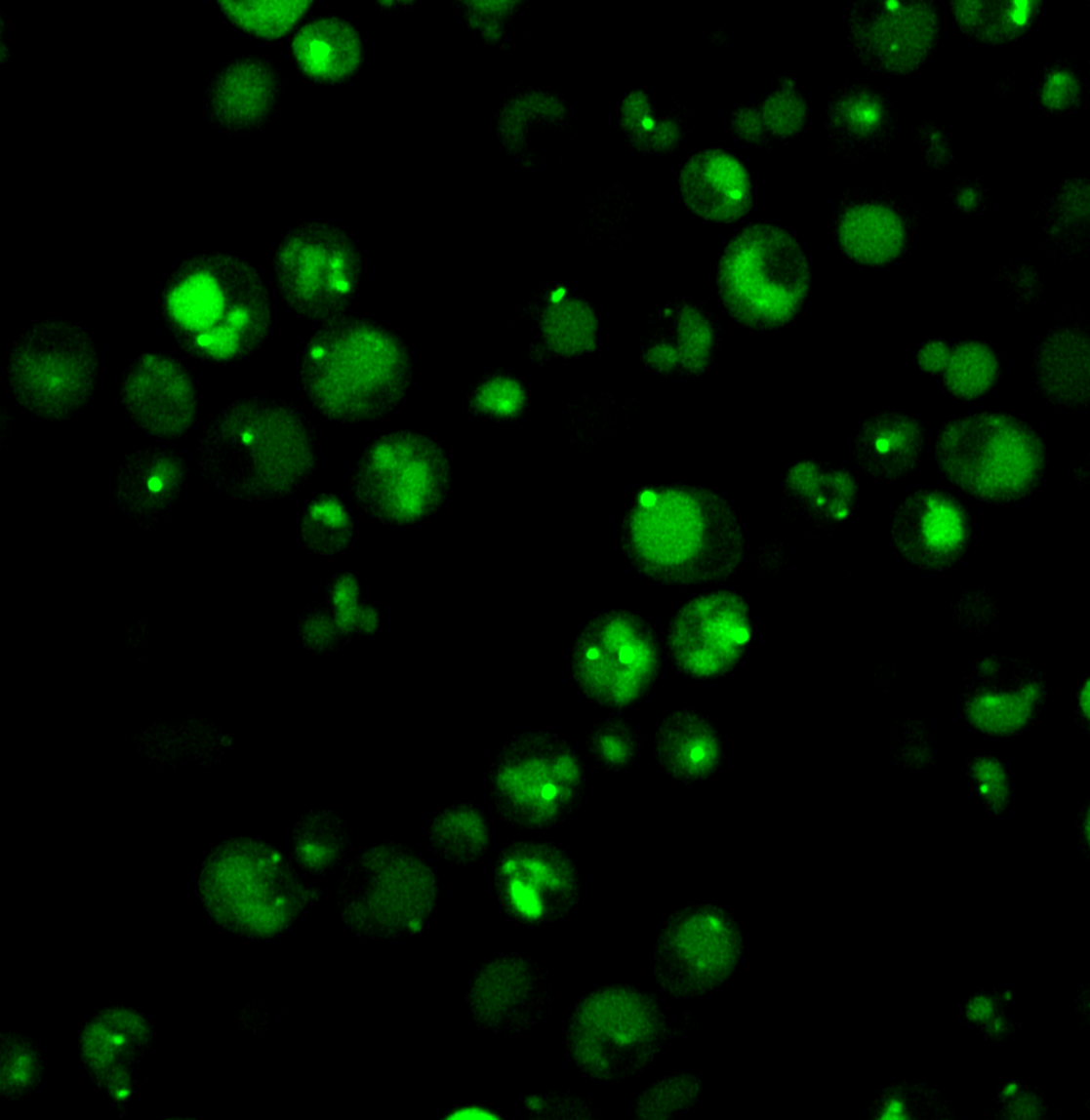

Supplement: Supplementary file 13 — Source Data for Figure 8 [file EMBJ-42-e113240-s003.zip › Figure 8/Figure 8F-WT-Rapa.tif]

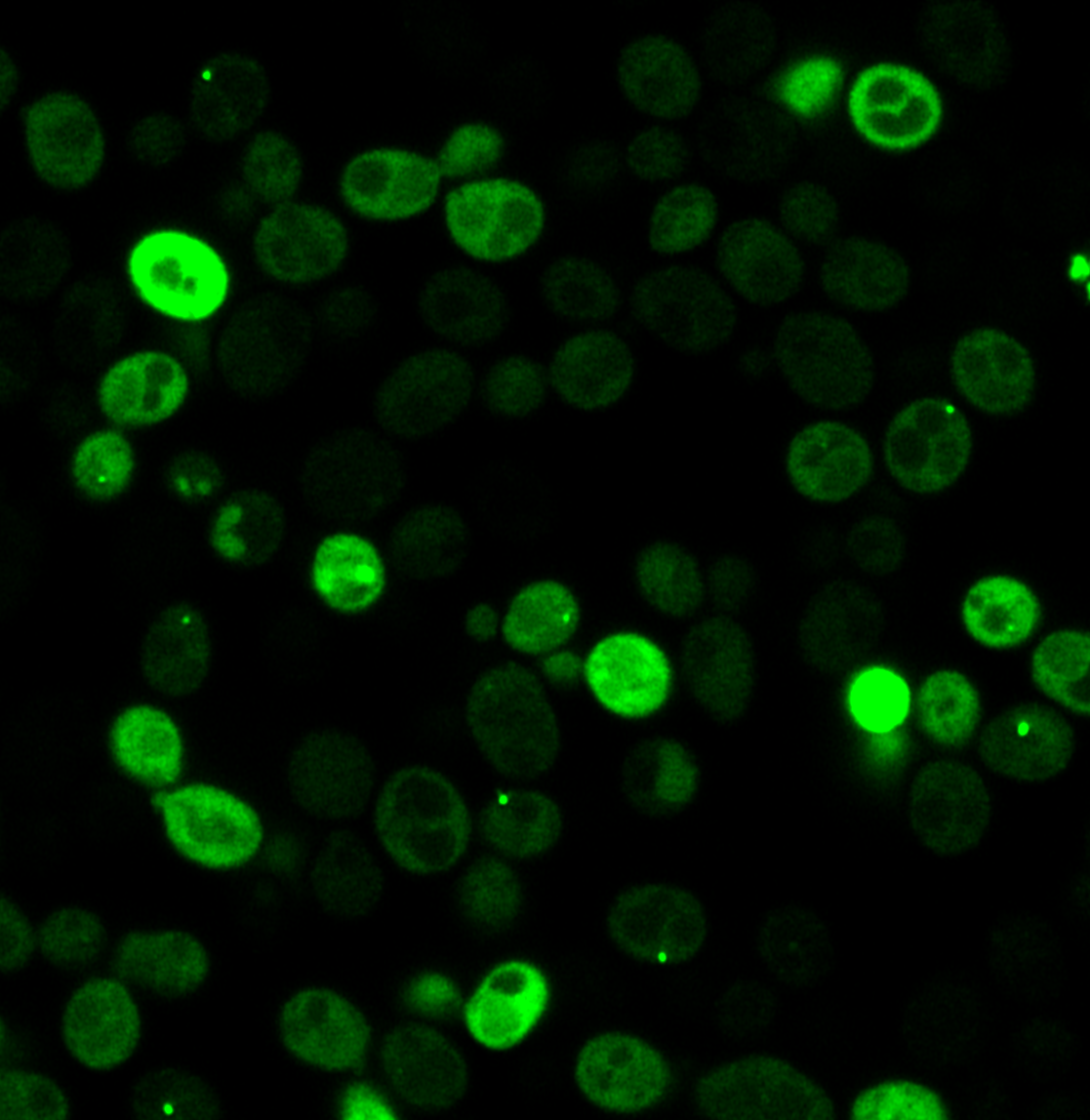

Supplement: Supplementary file 13 — Source Data for Figure 8 [file EMBJ-42-e113240-s003.zip › Figure 8/Figure 8F-Z1D-UT.tif]

Figure 8B

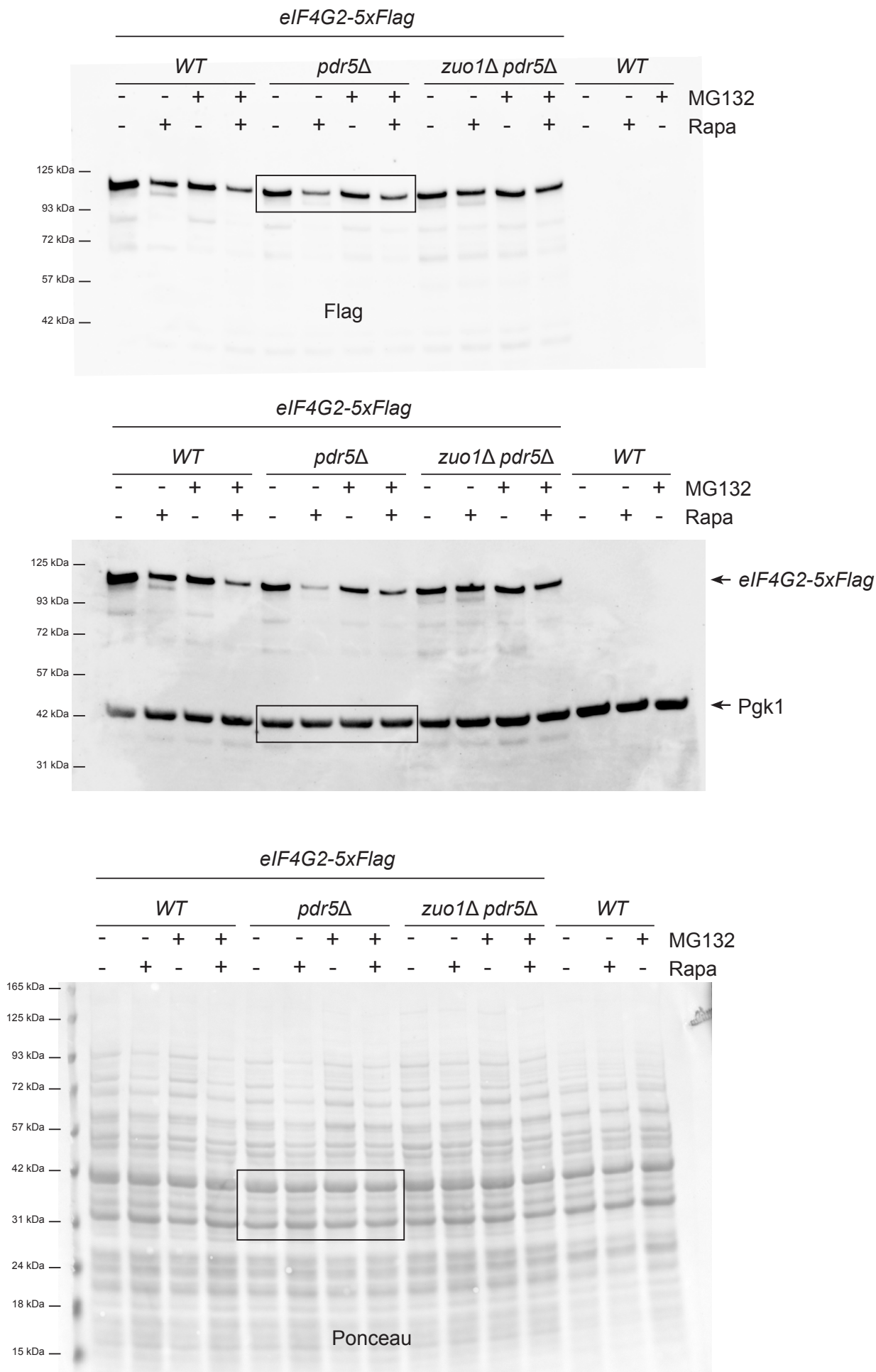

Figure 8C

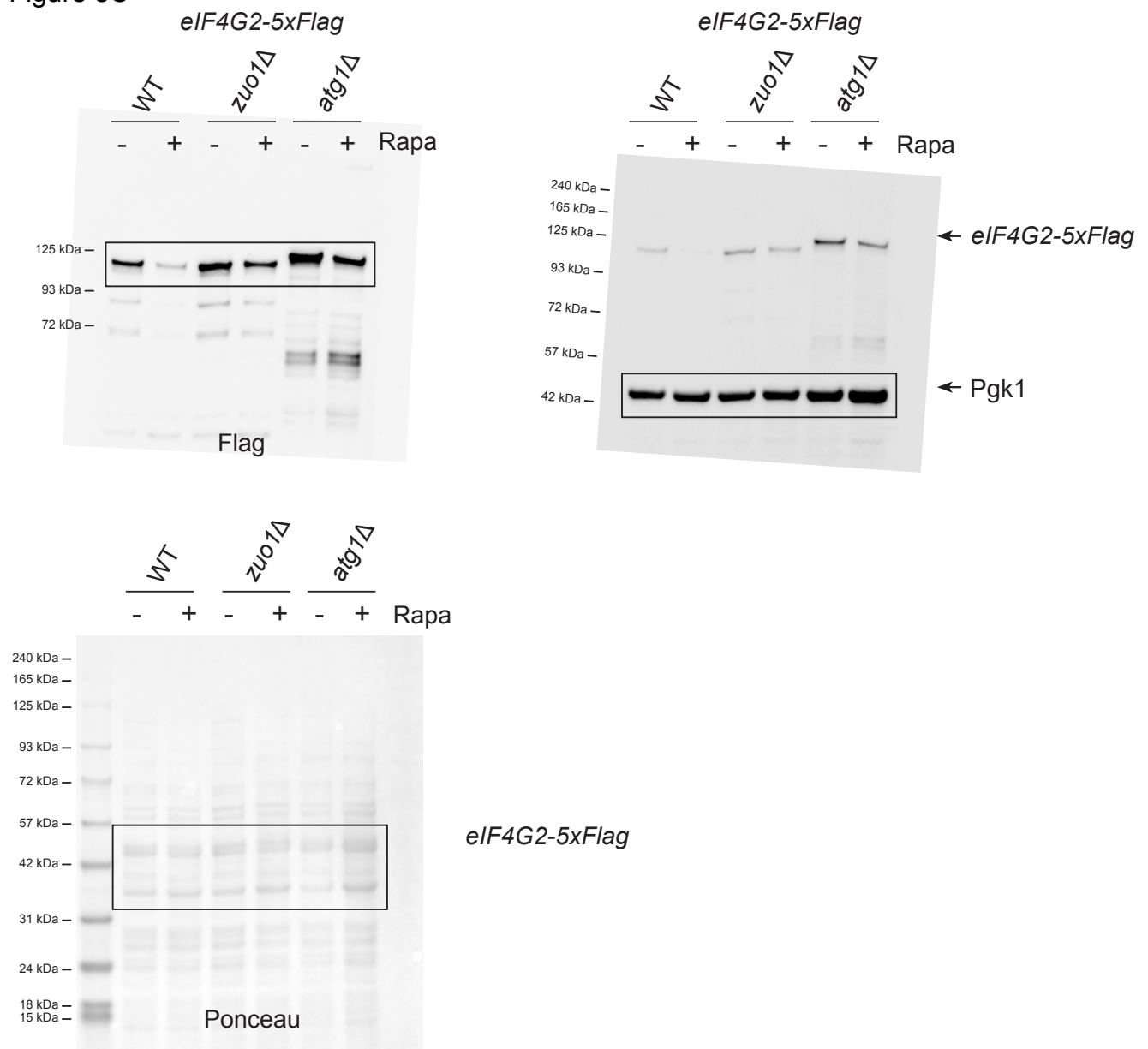

Figure 8D

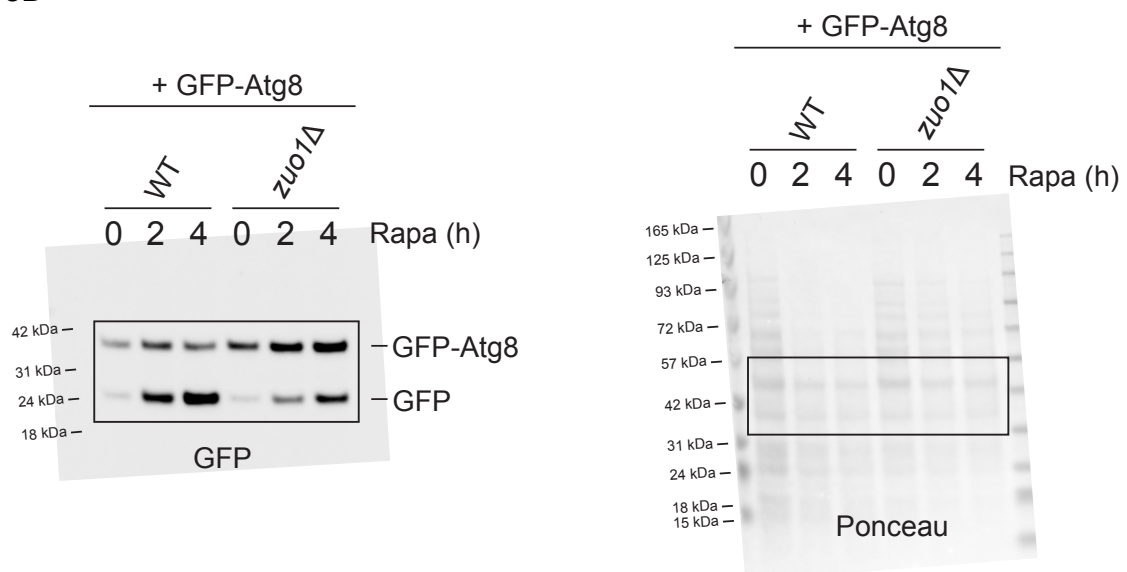

Figure 8D

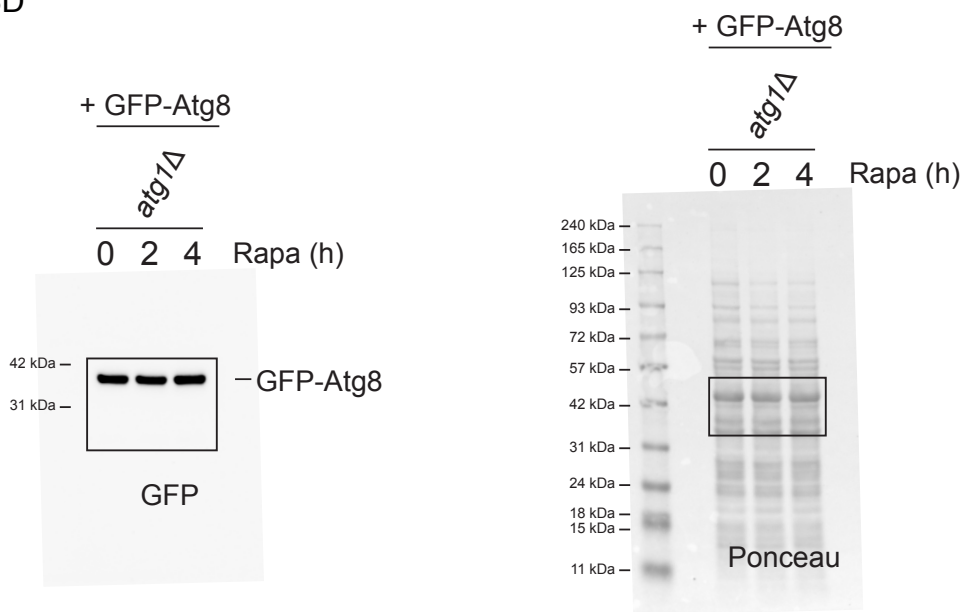

Figure 8F

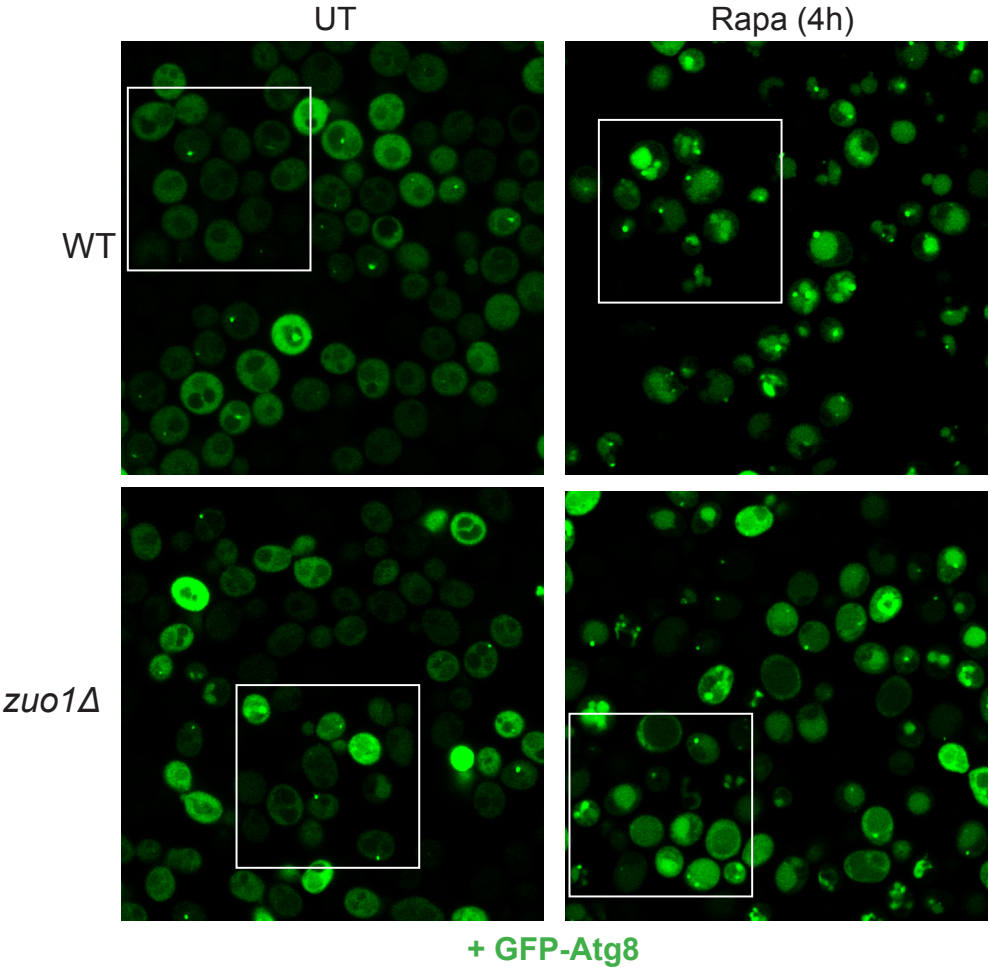

Supplement: Supplementary file 13 — Source Data for Figure 8 [file EMBJ-42-e113240-s003.zip › Figure 8/Raw - Figure 8.pdf]
