# Supplementary material for: The ribosome‐associated chaperone Zuo1 controls translation upon TORC1 inhibition
Source: EMBO J. 2023 Nov 20;42(24):e113240. doi: 10.15252/embj.2022113240 (PMC10711665; doi:10.15252/embj.2022113240)
Supplement: Supplementary file 14 [file EMBJ-42-e113240-s011.pdf]

THE EMBO JOURNAL

The ribosome-associated chaperone Zuo1 controls translation upon TORC1 inhibition

Ailsa Black, Thomas Williams, Flavie Soubigou, Ifeoluwapo Joshua, Houjiang Zhou, Frederic Lamoliatte, and Adrien Rousseau
DOI: 10.15252/embj.2022113240

Corresponding author: Adrien Rousseau (a.rousseau@dundee.ac.uk)

Review Timeline:

Submission Date:	8th Dec 22
Editorial Decision:	12th Dec 22
Appeal:	10th Feb 23
Editorial Decision:	21st Apr 23
Appeal:	11th May 23
Editorial Decision:	22nd May 23
Revision Received:	11th Sep 23
Editorial Decision:	15th Oct 23
Revision Received:	23rd Oct 23
Accepted:	26th Oct 23

Editor: Stefanie Boehm / Kelly Anderson

Transaction Report:

(Note: With the exception of the correction of typographical or spelling errors that could be a source of ambiguity, letters and reports are not edited. Depending on transfer agreements, referee reports obtained elsewhere may or may not be included in this compilation. Referee reports are anonymous unless the Referee chooses to sign their reports.)

1st Editorial Decision 12th Dec 2022

Dr. Adrien Rousseau
University of Dundee
Protein Phosphorylation and Ubiquitylation Unit
Dundee
United Kingdom

12th Dec 2022

Re: EMBOJ-2022-113240
The ribosome-associated chaperone Zuo1 controls translation upon TORC1 inhibition

Dear Dr. Rousseau,

Thank you for submitting your manuscript (EMBOJ-2022-113240) to The EMBO Journal. I have now read your study carefully and discussed the work with other members of the editorial team. However, I regret to inform you that we have decided not to pursue publication of this manuscript in The EMBO Journal.

We appreciate that you further characterize the ribosome-associated chaperone (RAC) Zuo1 and report that it is required for a reduction of translation upon rapamycin treatment and maintenance of proteostasis. Moreover, the decrease in eIF4G levels observed upon rapamycin treatment in wildtype is lost upon ZUO1 deletion and you propose that zuo1 null cells hereby sustain translation. We recognize that this study adds further insight into Zuo1 function upon TORC inhibition and indicates a role for RACs in mediating changes in translation upon stress. However, at the same time, we find that the molecular details of how Zuo1/RACs regulate this response remain to be defined, for example how Zuo1 affects eIF4G levels and Ssb1/2, which other factors are involved, or if this mode of regulation is specific for rapamycin/TORC inhibition or a general stress response. Thus, taking everything into consideration, we have concluded that the mechanistic insight at present is not sufficient to provide the degree of broader conceptual advance that would be required to warrant further consideration for publication in The EMBO Journal.

That being said, we appreciate the value of the findings to the scientific community and believe that your study is an excellent candidate for our partner journal Life Science Alliance (http://www.life-science-alliance.org/; our broad scope Open Access journal published in partnership between the EMBO-, Rockefeller University-, and Cold Spring Harbor Laboratory Presses). The editors of Life Science Alliance would be pleased to send your manuscript for in-depth peer review; no reformatting is required. We very much hope you will be interested in this option: please follow the link below for transfer.

Thank you for giving us the opportunity to consider your manuscript. I am sorry that we cannot be more positive on this occasion and I hope you will be interested in the transfer option.

Kind regards,

Stefanie Boehm

Stefanie Boehm
Editor
The EMBO Journal

*** As a service to authors, The EMBO Journal offers the possibility to directly transfer declined manuscripts to another EMBO Press title (EMBO Reports, EMBO Molecular Medicine, Molecular Systems Biology) or to the open access journal Life Science Alliance launched in partnership between EMBO Press, Rockefeller University Press and Cold Spring Harbor Laboratory Press. The full manuscript (including reviewer comments, where applicable and if chosen) will be automatically forwarded to the receiving journal, to allow for fast handling and a prompt decision on your manuscript. For more details of this service, and to transfer your manuscript to another EMBO title please follow this link:
Link Not Available

Dear Dr. Boehm,

We would like to resubmit the enclosed manuscript entitled "**The ribosome-associated chaperone Zuo1 controls translation upon TORC1 inhibition**" as an appeal of the initial decision (manuscript EMBOJ-2022-113240), as previously discussed with you. We understand that mechanistic insights about how Zuo1/RAC regulates eIF4G levels and whether this is a response specific to TORC1 inhibition or a more general stress response were missing in the previous version of our manuscript. We have now addressed this issue in the current version of the manuscript.

Additional results show that a comparable proportion of eIF4G2 mRNA was found to be associated with polysomes in *zuo1Δ* and WT cells, indicating that eIF4G is not translated to a greater extent in the absence of Zuo1 (Figure 8B). Thus, increased synthesis is not responsible for sustaining the pool of eIF4G upon rapamycin treatment in *zuo1Δ* cells, suggesting that eIF4G degradation may instead be impaired. We further show that proteasomal degradation does not substantially contribute to eIF4G degradation upon TORC1 inhibition (Figure 8C). In contrast, loss of autophagic degradation by deletion of Atg1, which is essential for autophagy, inhibited the degradation of eIF4G2 upon rapamycin treatment (Figure 8D). Together, these results show that autophagy-mediated eIF4G degradation is defective in *zuo1Δ* cells upon TORC1 inhibition.

As autophagy is required for eIF4G degradation, we then assessed whether the loss of eIF4G degradation is due to a defect in the general autophagy pathway in *zuo1Δ* cells. Using GFP-Atg8 autophagy reporter, we observed that autophagy induction upon rapamycin treatment was compromised in *zuo1Δ* cells compared to their WT counterpart (Figure 8E-H). Thus, the failure to reduce eIF4G levels in response to TORC1 inactivation in *zuo1Δ* cells is likely due to a deficiency in autophagy. To our knowledge, Zuo1 has not yet been linked to autophagy. This, in addition to the discovery that the RAC regulates translation shut-down upon TORC1 inhibition, brings significant new aspects into the regulation of proteostasis upon stress.

Regarding the role of Zuo1/RACs in other stresses, we now show that *zuo1Δ* cells displayed only very mild sensitivity to heat shock and were more resistant to tunicamycin-mediated endoplasmic reticulum (ER)-stress than WT cells (Figure S1A). This indicates that this is a specific response to TORC1 inhibition rather that a general stress response.

In summary, we feel that our additional data addresses all your previous concerns about how Zuo1 is controlling eIF4G proteins and whether this is general or specific stress response.

Dr. Adrien Rousseau
MRC Protein Phosphorylation & Ubiquitylation, University of Dundee
Sir James Black Centre, College of Life Sciences, Dow Street, Dundee, DD1 5EH, Scotland, UK
Email: arousseau@dundee.ac.uk Tel: +44 (0)1382 384109

Please let me know if you require any further information. I look forward to hearing from you in due course.

Your sincerely,

Adrien Rousseau

Dr. Adrien Rousseau
MRC Protein Phosphorylation & Ubiquitylation, University of Dundee
Sir James Black Centre, College of Life Sciences, Dow Street, Dundee, DD1 5EH, Scotland, UK
Email: arousseau@dundee.ac.uk Tel: +44 (0)1382 384109

1st Revision - Editorial Decision 21st Apr 2023

Dear Dr. Rousseau,

Thank you for submitting your manuscript for consideration by the EMBO Journal. It has now been seen by three referees whose comments are shown below.

As you will see the referees are concerned whether the conclusions are fully supported by the data and whether alternative explanations of the observations have been adequately explored. Given these opinions and the fact that the EMBO Journal can only afford to accept papers which receive enthusiastic support from a majority of referees, I am afraid we cannot offer to publish it here.

That said, given the general interest in this topic, we still found this work potentially suitable for our sister journal EMBO reports, in light of their focus on interesting key observations that do not necessarily need to be fully mechanistically followed up. I therefore briefly discussed the work with my EMBO reports colleague, Dr. Esther Schnapp, who would be happy to discuss a revision plan that does not necessarily address all points raised with new experimental data. Should you be interested in this option, please simply follow the transfer link; no reformatting is required but a preliminary point by point response could be uploaded for her review.

Thank you in any case for the opportunity to consider this manuscript. I am sorry we cannot be more positive on this occasion, but we hope nevertheless that you will find our referees' comments helpful.

Yours sincerely,

Kelly M Anderson, PhD
Editor
The EMBO Journal
k.anderson@embojournal.org

****

Referee #3:

In this paper the authors explore the underlying rationale behind the sensitivity of a yeast zuo1 deletion mutant to the widely-used inhibitor of TORC1 - rapamycin. In the zuo1 mutant they show that translation is not inhibited and eIF4G is not degraded. They show that autophagy is defective in the mutant and this appears to prevent eIF4G degradation. On the whole, the paper is well written and put together, although in my view there are some glaring omissions as detailed below.

1. A number of studies have shown that rapamycin causes the phosphorylation of eIF2alpha and that this leads to translation inhibition and activation of GCN4 translation (e.g. Cherkasova and Hinnebusch 2003 G&D; Staschke et al., 2010 JBC- amongst others). Therefore, in mutants that target this regulatory pathway to prevent eIF2alpha phosphorylation (SUI2 S51A or gcn2 deletion mutants) translation persists unabated after rapamycin treatment and under this scenario the mutants are more resistant to rapamycin than the parent strains. Judging from the kinetics and extent of these effects, the increase in eIF2alpha phosphorylation probably accounts for most, if not all, of the regulation of translation caused by rapamycin treatment.

The authors don't even mention the impact of rapamycin on eIF2/ eIF2B regulation, which strikes me as a serious omission given they are working on the impact of rapamycin on translation. As a result, they don't experimentally explore this. So they don't address whether a zuo1 mutant still exhibits increased eIF2alpha phosphorylation after rapamycin treatment. If not, does this mutant fail to increase eIF2 alpha phosphorylation in response to other stresses such as amino acid starvation (this could be tested using the drug sulfometuron methyl). Most importantly though, they don't address the discrepancy between the effects of zuo1 deletion, which prevents the translational inhibition and is sensitive to rapamycin, and the gcn2 delete or SUI2-S51A mutant strains which also prevent the translational inhibition but are resistant to rapamycin.
So I would envisage another figure of experimental work addressing these issues

2. The polysomes presented in the supplementary figures are technically very poor. There is substantial polysome run off evident in the untreated samples which would likely mask the run-off caused by rapamycin treatment (e.g. Barbet et al 1996 MBC or Di Como et al 1996 G&D - and many others). The most likely explanation for this is that the samples have warmed up slightly during extract preparation - I would guess during the bead beating lysis is the most likely stage where this would have occurred. Rather than lysing in a fastprep machine - maybe they should revert to a simple vortex where they can rapidly put into an ice/ water bath between each bead beating.

3. Could the authors use their proteomic data to assess which proteins are reduced like eIF4G after rapamycin treatment- is this common? Are there other proteins which have a similar profile to eIF4G?

4. I found the Introduction especially and some of the rest of the paper to be quite overhyped, it makes it sound as if TORC1 is the only player in yeast cells that co-ordinates growth, transcription and translation. Clearly this is not true and so I feel the authors should provide a more 'objective' appraisal of TORC1s role in the context of other components.

Referee #4:

In budding yeast, the ribosome-associated chaperone Zuo1 is required for survival in the presence of the mTOR inhibitor rapamycin. Budding yeast displays a conserved starvation-like response to mTOR inhibition, characterized by reduced protein synthesis and elevated autophagy. This manuscript argues that yeast lacking Zuo1 die in the presence of rapamycin because they fail to reduce translation. Analysis of a specific, misfolded substrate and bulk protein ubiquitylation both indicate that zuo1Δ cells maintain high levels of misfolded proteins during rapamycin treatment, while wildtype cells clear these proteins. It is argued that the persistence of misfolded proteins arises because zuo1Δ cells continue to translate new protein under conditions where wildtype cells reduce translation. Deletion of the co-translational Ssb chaperones, which depend on Zuo1, likewise allows high translation in the presence of rapamycin. It is argued that ribosome association of Zuo1 and interactions with Ssb proteins are required for this effect based on the phenotypes of known, structure-guided separation-of-function mutations in Zuo1. After surveying many potential client proteins, it is argued that persistent translation in zuo1Δ cells reflects a failure to degrade the essential translation initiation factor eIF4G. This eIF4G degradation appears to depend on autophagy more than the proteasome, and indeed, zuo1Δ impairs rapamycin-induced autophagy.

The implication of ribosome-associated folding chaperones with translational control in proteostasis is an interesting and novel contribution. Data presented in the manuscript robustly support this basic conclusion, although the links from Zuo1 (and Ssb?), to autophagy, to eIF4G depletion are not yet clear. Greater clarity on this connection would be valuable, but may lie beyond the scope of this work. I would support publication of this manuscript providing the more specific concerns listed below are addressed.

1. In discussing the response of yeast to ER stress, it is written that "[cells treated with] tunicamycin [are] mainly relying on eIF2 phosphorylation for stress survival."

However, budding yeast does not encode a PERK orthologue and does not phosphorylate eIF2α in response to ER stress-it relies solely on the Ire1 branch of the UPR. Translational effects of ER stress may depend on ribosome ubiquitylation (DOI: 10.1038/s41598-020-76239-3), but in any case yeast do not rely on eIF2 phosphorylation for survival in tunicamycin.

2. In many blots (e.g., Figure 3A, Figure 3B, and Figure 4D) it appears that zuo1Δ cells treated with rapamycin actually show elevated translation. Is this accurate? What would be the explanation?

3. As a related point, ssb1/2Δ in Figure 4D, appears to show consistently higher puromycylation, relative to wildtype, and similar to zuo1Δ in rapamycin. Does this reflect an even more direct or constitutive role for Ssb in promoting autophagy?

4. Cycloheximide treatment clearly reduces levels of ubiquitylated proteins in zuo1Δ but levels remain higher than those in wildtype cells treated with cycloheximide. This effect is even stronger in ssb1/2Δ cells.

5. The data from ZUO1 separation-of-function mutations that prevent ribosome binding (RR-AA) or Ssb interaction (HPD-AAA) seem over-interpreted. Notably, both individual mutations are expected to abolish the ribosome-associated function of Zuo1 and it is unclear why loss of ribosome binding and loss of Ssb interaction would synergize.

The manuscript argues that, "It has been reported that even very low levels of Zuo1 are enough to preserve its function suggesting that single mutations may not be enough to fully abrogate Zuo1 activity". However, normal levels of non-functional protein are not the same as low levels of fully functional protein.

6. As a related point, the levels of Zuo1 mutant proteins should be assessed to ensure that their abundance is roughly equivalent to wildtype.

7. Changes in the Zuo1 interactome after rapamycin treatment may be driven most strongly by changes in the overall translatome: Aro10 and Cps1 are certainly induced under starvation conditions and it seems likely that Cpa2 and many of the other metabolic enzymes identified in the screen are likewise changing in expression. This limitation needs to be discussed when interpreting results from Figure 6.

8. In arguing against translational effects on eIF4G2 synthesis, it is argued that:
"In both untreated and rapamycin-treated conditions, a comparable proportion of eIF4G2 mRNA was found to be associated with polysomes in zuo1Δ and WT cells, indicating that eIF4G is not translated to a greater extent in the absence of Zuo1 (Figure 8B). Thus, increased synthesis is not responsible for sustaining the pool of eIF4G upon rapamycin treatment in zuo1Δ cells."

However, a change in the [typical] number of ribosomes translating an mRNA can substantially change protein levels with no change in the fraction of polysome-associated mRNA.

9. The argument that elevated eIF4G explained the zuo1Δ phenotype would be much stronger if more specific assays, such as persistent protein synthesis (Figures 3A, 3B, 4D, 5C, 5G, 6E) or ubiquitylated protein accumulation (Figures 1C, 3C, 3E, 5B, 5F, 6D) were performed for eIF4G overexpression.

10. Could a TIF4632-GFP fusion be used to test more specifically whether eIF4G is targeted for autophagy?

** As a service to authors, EMBO Press provides authors with the possibility to transfer a manuscript that one journal cannot offer to publish to another EMBO publication or the open access journal Life Science Alliance launched in partnership between EMBO Press, Rockefeller University Press and Cold Spring Harbor Laboratory Press. The full manuscript and if applicable, reviewers' reports, are automatically sent to the receiving journal to allow for fast handling and a prompt decision on your manuscript. For more details of this service, and to transfer your manuscript please click on Link Not Available. **

Please do not share this URL as it will give anyone who clicks it access to your account.

Referee #3:

In this paper the authors explore the underlying rationale behind the sensitivity of a yeast zuo1 deletion mutant to the widely-used inhibitor of TORC1 - rapamycin. In the zuo1 mutant they show that translation is not inhibited and eIF4G is not degraded. They show that autophagy is defective in the mutant and this appears to prevent eIF4G degradation. On the whole, the paper is well written and put together, although in my view there are some glaring omissions as detailed below.

1. A number of studies have shown that rapamycin causes the phosphorylation of eIF2alpha and that this leads to translation inhibition and activation of GCN4 translation (e.g. Cherkasova and Hinnebusch 2003 G&D; Staschke et al., 2010 JBC- amongst others). Therefore, in mutants that target this regulatory pathway to prevent eIF2alpha phosphorylation (SUI2 S51A or gcn2 deletion mutants) translation persists unabated after rapamycin treatment and under this scenario the mutants are more resistant to rapamycin than the parent strains. Judging from the kinetics and extent of these effects, the increase in eIF2alpha phosphorylation probably accounts for most, if not all, of the regulation of translation caused by rapamycin treatment.

We agree with the reviewer that eIF2alpha phosphorylation is important for the regulation of translation upon rapamycin treatment. However, it does not seem to account for most, if not all, of the regulation of translation, as shown in the paper mentioned by the reviewer (Cherkasova and Hinnebusch 2003 G&D): “Thus, it appears that phosphorylation of eIF2 by GCN2 is responsible for about 50% of the inhibition of translation initiation by rapamycin”. Thus, eiF2alpha is definitely central in regulating translation upon TORC1 inhibition but additional signalling pathways are also involved, such as the RAC/Ssb chaperone system.

The authors don't even mention the impact of rapamycin on eIF2/ eIF2B regulation, which strikes me as a serious omission given they are working on the impact of rapamycin on translation. As a result, they don't experimentally explore this. So they don't address whether a zuo1 mutant still exhibits increased eIF2alpha phosphorylation after rapamycin treatment.

This is a fair point raised by the reviewer and we agree that TORC1 is not the only signalling pathway involved in the regulation of translation upon stress. We have not included data on eIF2alpha regulation in the previous version of the manuscript as we wanted to keep the story focused on TORC1 complex. We have now realised that it may have been perceived as an omission and this has been addressed in the new version of the manuscript. We have now data showing that the induction of eIF2alpha phosphorylation following rapamycin remains unchanged in *zuo1Δ* cells compared to WT cells (see below), indicating that the role of Zuo1 in regulating translation upon TORC1 inhibition is eIF2alpha-independent. In addition, we have now discussed the role of eIF2alpha in regulating translation upon stress in the main text, as suggested by the reviewer.

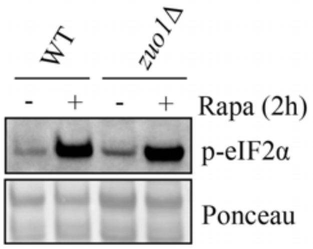

If not, does this mutant fail to increase eIF2 alpha phosphorylation in response to other stresses such as amino acid starvation (this could be tested using the drug sulfometuron methyl). Most importantly though, they don't address the discrepancy between the effects of zuo1 deletion, which prevents the translational inhibition and is sensitive to rapamycin, and the gcn2 delete or SUI2-S51A mutant strains which also prevent the translational inhibition but are resistant to rapamycin.

We don't think that there is any discrepancy between the fact that S51A-eIF2alpha mutant is more resistant to rapamycin while *zuo1Δ* cells are sensitive to rapamycin, this can be easily explain by the additional role of Zuo1 in regulating protein folding at the ribosome and autophagy. In agreement with that, *zuo1Δ* cells are slow growth while S51A-eIF2alpha mutant cells are not, highlighting the stronger phenotype of *zuo1Δ* cells in maintain protein homeostasis. This could be discussed in the paper, and we could also generate S51A CRISPR KI mutation of SUI2 (and GCN2 deletion) in WT and *zuo1Δ* cells (if viable) to see the eIF2alpha mutation restore the rapamycin sensitivity of *zuo1Δ* cells.

So I would envisage another figure of experimental work addressing these issues

Experimental plan:

- Discuss the role of eIF2alpha in regulating translation upon TORC1 inhibition in the main text.
- Add the data showing that phosphorylation of eIF2alpha is not affected by Zuo1 deletion.
- Make CRISPR-Cas9 mutation of SUI2 (S51A) and GCN2 deletion in WT and *zuo1Δ* cells (if viable), and monitor cell growth upon rapamycin treatment.

2. The polysomes presented in the supplementary figures are technically very poor. There is substantial polysome run off evident in the untreated samples which would likely mask the run-off caused by rapamycin treatment (e.g. Barbet et al 1996 MBC or Di Como et al 1996 G&D - and many others). The most likely explanation for this is that the samples have warmed up slightly during extract preparation - I would guess during the bead beating lysis is the most likely stage where this would have occurred. Rather than lysing in a fastprep machine - maybe they should revert to a simple vortex where they can rapidly put into an ice/ water bath between each bead beating.

Polysome profiling will be repeated using a vortex instead of a fastprep, as suggested by the reviewer.

3. Could the authors use their proteomic data to assess which proteins are reduced like eIF4G after rapamycin treatment- is this common? Are there other proteins which have a similar profile to eIF4G?

Unfortunately, this cannot be done, as our proteomic data identified protein co-immunoprecipitating with Zuo1 (elution) and not the total level of proteins in the input.

4. I found the Introduction especially and some of the rest of the paper to be quite overhyped, it makes it sound as if TORC1 is the only player in yeast cells that co-ordinates growth, transcription and translation. Clearly this is not true and so I feel the authors should provide a more 'objective' appraisal of TORC1s role in the context of other components.

We agree with the reviewer and the contribution of eIF2alpha in regulating translation upon TROC1 inhibition will be discussed in the new version of the manuscript.

Referee #4:

In budding yeast, the ribosome-associated chaperone Zuo1 is required for survival in the presence of the mTOR inhibitor rapamycin. Budding yeast displays a conserved starvation-like response to mTOR inhibition, characterized by reduced protein synthesis and elevated autophagy. This manuscript argues that yeast lacking Zuo1 die in the presence of rapamycin because they fail to reduce translation. Analysis of a specific, misfolded substrate and bulk protein ubiquitylation both indicate that zuo1Δ cells maintain high levels of misfolded proteins during rapamycin treatment, while wildtype cells clear these proteins. It is argued that the persistence of misfolded proteins arises because zuo1Δ cells continue to translate new protein under conditions where wildtype cells reduce translation. Deletion of the co-translational Ssb chaperones, which depend on Zuo1, likewise allows high translation in the presence of rapamycin. It is argued that ribosome association of Zuo1 and interactions with Ssb proteins are required for this effect based on the phenotypes of known, structure-guided separation-of-function mutations in Zuo1. After surveying many potential client proteins, it is argued that persistent translation in zuo1Δ cells reflects a failure to degrade the essential translation initiation factor eIF4G. This eIF4G degradation appears to depend on autophagy more than the proteasome, and indeed, zuo1Δ impairs rapamycin-induced autophagy.

The implication of ribosome-associated folding chaperones with translational control in proteostasis is an interesting and novel contribution. Data presented in the manuscript robustly support this basic conclusion, although the links from Zuo1 (and Ssb?), to autophagy, to eIF4G depletion are not yet clear. Greater clarity on this connection would be valuable, but may lie beyond the scope of this work. I would support publication of this manuscript providing the more specific concerns listed below are addressed.

1. In discussing the response of yeast to ER stress, it is written that "[cells treated with] tunicamycin [are] mainly relying on eIF2 phosphorylation for stress survival."

However, budding yeast does not encode a PERK orthologue and does not phosphorylate eIF2α in response to ER stress-it relies solely on the Ire1 branch of the UPR. Translational

effects of ER stress may depend on ribosome ubiquitylation (DOI: 10.1038/s41598-020-76239-3), but in any case yeast do not rely on eIF2 phosphorylation for survival in tunicamycin.

This has been modified in the main text: "This may be due to TORC1 being still active at 37°C and tunicamycin mainly relying on Ire1 branch for stress survival."

2. In many blots (e.g., Figure 3A, Figure 3B, and Figure 4D) it appears that zuo1Δ cells treated with rapamycin actually show elevated translation. Is this accurate? What would be the explanation?

Yes, this is accurate. We think that this is due to puromycylated-nascent chains being less degraded in *zuo1Δ* cells compared to WT cells. In agreement with that, we have data showing that puromycylated-nascent chains are indeed less efficiently degrade in *zuo1Δ* cells compared to WT cells (see below). This will be added and discussed in the new version of the manuscript.

3. As a related point, ssb1/2Δ in Figure 4D, appears to show consistently higher puromycylation, relative to wildtype, and similar to zuo1Δ in rapamycin. Does this reflect an even more direct or constitutive role for Ssb in promoting autophagy?

Yes, we think that it is indeed due to autophagy defects of *ssb1/2Δ* cells. This will be tested.

Experimental plan:

- Analyse the cleavage of GFP-ATG8 in *ssb1/2Δ* cells compared to WT cells.

4. Cycloheximide treatment clearly reduces levels of ubiquitylated proteins in zuo1Δ but levels remain higher than those in wildtype cells treated with cycloheximide. This effect is even stronger in ssb1/2Δ cells.

We agree with the reviewer that the rescue is not complete, as it is often the case in such experiments. This has been specified in the main text: "Confirming this, cycloheximide treatment inhibited translation in both WT and *zuo1Δ* cells, even in the presence of rapamycin (Figure 3B), and partly rescued the impaired clearance of polyubiquitinated proteins observed in *zuo1Δ* cells following TORC1 inhibition (Figure 3C).". Regarding *ssb1/2Δ* cells, we said that cycloheximide improves their clearance of polyubiquitinated proteins, and we think that this is correct.

We think that this may be due to the autophagy defects observed in *zuo1Δ* cells and this will be now discussed in the discussion part of the manuscript.

5. The data from ZUO1 separation-of-function mutations that prevent ribosome binding (RR-AA) or Ssb interaction (HPD-AAA) seem over-interpreted. Notably, both individual mutations are expected to abolish the ribosome-associated function of Zuo1 and it is unclear why loss of ribosome binding and loss of Ssb interaction would synergize.

Hundley et al., reported that mutation in the J-domain of Zuo1 still retains residual function: “Strains expressing this mutant Zuo1 grew slowly and showed sensitivity to paromomycin, but were not as defective as mutants completely lacking the protein (Fig. 4A). The mutant protein was still associated with ribosomes, as was Ssz1 (Fig. 4B).” (PMID: 11929993). Similar observation has been made with the ribosome-binding deficient mutant of Zuo1 (Zuo1-RR-AA) (PMID: 25639645). The rationale of the double mutant is that each mutation will strongly impede Zuo1 function and, together, will almost completely abrogate its activity. This will be clarified in the manuscript.

The manuscript argues that, "It has been reported that even very low levels of Zuo1 are enough to preserve its function suggesting that single mutations may not be enough to fully abrogate Zuo1 activity". However, normal levels of non-functional protein are not the same as low levels of fully functional protein.

This sentence will be removed to prevent any confusion.

6. As a related point, the levels of Zuo1 mutant proteins should be assessed to ensure that their abundance is roughly equivalent to wildtype.

This will be tested experimentally: we will add a Flag tag on WT and mutants Zuo1 and analyse their expression levels by immunoblot. This will be added to the manuscript.

7. Changes in the Zuo1 interactome after rapamycin treatment may be driven most strongly by changes in the overall translatome: Aro10 and Cps1 are certainly induced under starvation conditions and it seems likely that Cpa2 and many of the other metabolic enzymes identified in the screen are likewise changing in expression. This limitation needs to be discussed when interpreting results from Figure 6.

We agree with the point raised by the reviewer, and this will be discussed in the main manuscript, as suggested.

8. In arguing against translational effects on eIF4G2 synthesis, it is argued that:
"In both untreated and rapamycin-treated conditions, a comparable proportion of eIF4G2 mRNA was found to be associated with polysomes in zuo1Δ and WT cells, indicating that eIF4G is not translated to a greater extent in the absence of Zuo1 (Figure 8B). Thus, increased synthesis is not responsible for sustaining the pool of eIF4G upon rapamycin treatment in zuo1Δ cells."

However, a change in the [typical] number of ribosomes translating an mRNA can substantially change protein levels with no change in the fraction of polysome-associated mRNA.

We agree with the reviewer that, even it is often the case, a change in the fraction of polysome-associated mRNA is not always correlated with translation. To prevent any misunderstanding, we will remove Figure 8B from the manuscript. This will not change our conclusion, as we show that the regulation is posttranscriptional and autophagy-dependent.

9. The argument that elevated eIF4G explained the zuo1Δ phenotype would be much stronger if more specific assays, such as persistent protein synthesis (Figures 3A, 3B, 4D, 5C, 5G, 6E) or ubiquitylated protein accumulation (Figures 1C, 3C, 3E, 5B, 5F, 6D) were performed for eIF4G overexpression.

This will be tested experimentally: We will analyse the levels of ubiquitinylated proteins in cells overexpressing or not eIF4G and treated or not with rapamycin.

10. Could a TIF4632-GFP fusion be used to test more specifically whether eIF4G is targeted for autophagy?

This will be tested experimentally: We will express eIF4G-GFP in WT and *zuo1Δ* cells and analyse free GFP levels under rapamycin treatment.

2nd Revision - Editorial Decision 22nd May 2023

Dear Adrien,

Thank you for submitting your manuscript for reconsideration by the EMBO Journal. I have discussed your plan to address the referee concerns with the editorial team and with the referees and all have agreed to have another look at a revised version of this manuscript.

It is EMBO Journal policy to allow a single round of revision only and therefore, acceptance or rejection of the manuscript will depend on the completeness of your responses in this revised version. Upon reviewing your revision plan, Referee 3 was concerned that your response to Q3 implied you have not tested whether proteins in the whole cell extract change. I wanted to make you aware before resubmission that this referee views this as a key control to determine whether changes in elutions are not simply due to changes in the total protein level.

In your revision, please include a detailed point-by-point response to the referees' comments. Please also bear in mind that this will form part of the Review Process File, and will therefore be available online to the community. For more details on our Transparent Editorial Process, please visit our website: https://www.embo.org/embo-press I have also attached a guide for revisions to this email for your convenience.

We generally allow three months as standard revision time. As a matter of policy, competing manuscripts published during this period will not negatively impact on our assessment of the conceptual advance presented by your study. However, we request that you contact the editor as soon as possible upon publication of any related work, to discuss how to proceed. Should you foresee a problem in meeting this three-month deadline, please let us know in advance and we may be able to grant an extension.

Thank you for the opportunity to consider your work for publication. I look forward to your revision.

Yours sincerely,

Kelly M Anderson, PhD
Editor
The EMBO Journal
k.anderson@embojournal.org

Further information is available in our Guide For Authors: https://www.embopress.org/page/journal/14602075/authorguide

The revision must be submitted online within 90 days;

POINTS-BY-POINTS RESPONSE TO REVIEWERS:

We thank the editors and the reviewers for their careful reading of the manuscript and their constructive remarks. We have taken all the comments into consideration to strengthen and clarify the manuscript. We feel that the manuscript has been significantly improve as a consequence. Please find below a detailed point-by-point response to all comments (reviewers' comments in black, our replies in blue).

Referee #3:

In this paper the authors explore the underlying rationale behind the sensitivity of a yeast zuo1 deletion mutant to the widely-used inhibitor of TORC1 - rapamycin. In the zuo1 mutant they show that translation is not inhibited and eIF4G is not degraded. They show that autophagy is defective in the mutant and this appears to prevent eIF4G degradation. On the whole, the paper is well written and put together, although in my view there are some glaring omissions as detailed below.

We would like to thank the reviewer for valuable and constructive comments and suggestions. We have made substantial revision to the manuscript to address them. We direct the reviewer to our responses to each individual question below.

1. A number of studies have shown that rapamycin causes the phosphorylation of eIF2alpha and that this leads to translation inhibition and activation of GCN4 translation (e.g. Cherkasova and Hinnebusch 2003 G&D; Staschke et al., 2010 JBC- amongst others). Therefore, in mutants that target this regulatory pathway to prevent eIF2alpha phosphorylation (SUI2 S51A or gcn2 deletion mutants) translation persists unabated after rapamycin treatment and under this scenario the mutants are more resistant to rapamycin than the parent strains. Judging from the kinetics and extent of these effects, the increase in eIF2alpha phosphorylation probably accounts for most, if not all, of the regulation of translation caused by rapamycin treatment.

We agree with the reviewer that eIF2alpha phosphorylation is important for the regulation of translation upon rapamycin treatment. However, it does not seem to account for most, if not all, of the regulation of translation, as shown in the paper mentioned by the reviewer (Cherkasova and Hinnebusch 2003 G&D): “Thus, it appears that phosphorylation of eIF2 by GCN2 is responsible for about 50% of the inhibition of translation initiation by rapamycin”. Thus, eiF2alpha is regulating translation upon TORC1 inhibition but additional signalling pathways are also involved, such as the RAC/Ssb chaperone system described here.

The authors don't even mention the impact of rapamycin on eIF2/ eIF2B regulation, which strikes me as a serious omission given they are working on the impact of rapamycin on translation. As a result, they don't experimentally explore this. So they don't address whether a zuo1 mutant still exhibits increased eIF2alpha phosphorylation after rapamycin treatment.

This is a fair point raised by the reviewer and we agree that TORC1 is not the only signalling pathway involved in the regulation of translation upon stress. In the first version of the manuscript, we have not included data on eIF2alpha regulation as we wanted to keep the story focused on TORC1 complex. We have now realised that it may have been perceived as an

omission and this has been addressed in the new version of the manuscript. We have now included data showing that the induction of eIF2alpha phosphorylation following rapamycin treatment remains unchanged in *zuo1Δ* cells compared to WT cells, indicating that the role of Zuo1 in regulating translation upon TORC1 inhibition is eIF2alpha-independent: "Moreover, phosphorylation of Sui2 upon rapamycin treatment was unaffected by the absence of Zuo1 (Figure 7H). Together, these results show that, following TORC1 inhibition, *zuo1Δ* cells may be sustaining translation through the remaining pool of eIF4G, independently of Sui2.".

In addition, we have now discussed the role of eIF2alpha in regulating translation upon stress in the main text, as suggested by the reviewer (see below response to point 4).

If not, does this mutant fail to increase eIF2 alpha phosphorylation in response to other stresses such as amino acid starvation (this could be tested using the drug sulfometuron methyl). Most importantly though, they don't address the discrepancy between the effects of zuo1 deletion, which prevents the translational inhibition and is sensitive to rapamycin, and the gcn2 delete or SUI2-S51A mutant strains which also prevent the translational inhibition but are resistant to rapamycin. So I would envisage another figure of experimental work addressing these issues

We don't think that there is any discrepancy between the fact that Sui2-S51A mutant is more resistant to rapamycin while *zuo1Δ* cells are sensitive to rapamycin, this can be easily explain by the additional role of Zuo1 in regulating protein folding at the ribosome and autophagy. In agreement with that, *zuo1Δ* cells are slow growth while the Sui2-S51A mutant cells are not, highlighting the stronger phenotype of *zuo1Δ* cells in maintain protein homeostasis. In addition, Sui2-S51A mutant is more sensitive to histidine-starvation (imposed by the addition of 3-AT) which also induces reduction of translation following Sui2 phosphorylation at S51 (https://doi.org/10.1016/j.molcel.2021.02.037), so impaired translation shutdown is not always associated with higher resistance upon drugs mimicking nutrient starvation. Nonetheless, this is an interesting point raised by the reviewer and we have performed new experiments to investigate the crosslink between Zuo1 and eIF2α pathways.

We first monitored whether Zuo1 deletion has altered phosphorylation of SUI2 at S51 using phospho-specific antibody. We found that, as expected the phosphorylation of Sui2 at S51 is increased upon rapamycin. This was not impacted by ZUO1 deletion indicating that the translation defect of *zuo1Δ* cells upon TORC1 inhibition is not due to the absence of Sui2 phosphorylation (Figure 7H). Next, Sui2-S51A mutation has been made in WT and *zuo1Δ* cells to define whether blocking Sui2 phosphorylation at S51 is beneficial for the translation and growth defects of *zuo1Δ* cells. We used CRISPR-Cas9 gene editing to introduce the S51A mutation at the endogenous SUI2 locus. We observed that when the S51A mutation is introduced at the endogenous locus, it was not inducing rapamycin resistance (Figure 7G). While this is different to previous works, all papers cited by the reviewer use a strain expressing the WT or mutant version of Sui2 on a vector in a Sui2 deletion background, thus SUI2 expression is likely higher than the endogenous expression and it has lost its endogenous regulation, potentially explaining the discrepancy. In agreement with that, we discussed with Claudio De Virgilio's lab that has generated similar CRISPR-Cas9 Sui2-S51A mutant and while they observe sensitivity to 3-AT (https://doi.org/10.1016/j.molcel.2021.02.037) as previously described, they did not observe higher resistance to rapamycin, unpublished data). Further to that, Sui2-S51A mutation was not rescuing translation and growth defects of *zuo1Δ* cells upon rapamycin treatment, while

completely abolishing phosphorylation at S51 (Figure 7H). Analysing another clone in both WT and *zuo1Δ* background showed similar growth results (Figure S4D, E). This indicates that CRISPR-Cas9-mediated SUI2-S51A mutation is not beneficial for *zuo1Δ* cells upon rapamycin treatment. In summary, our latest data show that the translation defect of *zuo1Δ* cells is independent of Sui2 regulation. This has been added to the new version of the manuscript.

2. The polysomes presented in the supplementary figures are technically very poor. There is substantial polysome run off evident in the untreated samples which would likely mask the run-off caused by rapamycin treatment (e.g. Barbet et al 1996 MBC or Di Como et al 1996 G&D - and many others). The most likely explanation for this is that the samples have warmed up slightly during extract preparation - I would guess during the bead beating lysis is the most likely stage where this would have occurred. Rather than lysing in a fastprep machine - maybe they should revert to a simple vortex where they can rapidly put into an ice/ water bath between each bead beating.

We thank the reviewer for the suggestion. We have now preformed polysome profiling implementing the reviewer's suggestion and this has indeed significantly prevented ribosome run-off (Figure S2). The figure and method have now been updated accordingly.

3. Could the authors use their proteomic data to assess which proteins are reduced like eIF4G after rapamycin treatment- is this common? Are there other proteins which have a similar profile to eIF4G?

This is an interesting point raised by the reviewer. While our data show the ability of other proteins to bind to Zuo1-GFP and thus cannot inform about changes of total protein levels, such datasets have been published and are readily available. We then searched our candidate Zuo1 partners (Figure 6C) and proteins belonging to the eIF4F complex for significant changes in their total protein levels upon rapamycin treatment (https://www.science.org/doi/10.1126/scisignal.2002548). We only found two proteins, eIF4G1 and Ura7, to have reduced levels upon rapamycin treatment (Figure S3). Tagging endogenous Ura7, we found that its levels was not affected by rapamycin treatment (Figure S3D). This suggests that the regulation of protein stability by Zuo1 is not a common phenomenon, and it is rather specific. This result has been added to the new version of the manuscript.

4. I found the Introduction especially and some of the rest of the paper to be quite overhyped, it makes it sound as if TORC1 is the only player in yeast cells that co-ordinates growth, transcription and translation. Clearly this is not true and so I feel the authors should provide a more 'objective' appraisal of TORC1s role in the context of other components.

We agree with the reviewer and the contribution of eIF2alpha in regulating translation has now been integrated to the new version of the manuscript, especially in the introduction: "Ternary complex formation and eIF4F complex formation are two main aspects of translation regulation upon TORC1 inhibition. Ternary complex formation is probably the most well-characterised mode of regulation. Upon cell stress, the α subunit of eIF2 referred to as SUI2 is phosphorylated on a conserved serine residue (Serine 51), which increases its affinity for its guanine nucleotide exchange factor (GEF) eIF2B[25]. This inhibits eIF2B GEF activity, preventing the exchange of GDP for GTP on eIF2. The GTP-bound form of eIF2 has a much greater affinity for Met-tRNAi than

eIF2-GDP and so ternary complex regeneration is severely restricted by phosphorylation of eIF2α[26]. In yeast, Gcn2 is responsible for eIF2α phosphorylation. Loss of TORC1 activity or binding by uncharged tRNA results in dephosphorylation and activation of Gcn2 which phosphorylates eIF2α at S51. This induces a global reduction of protein synthesis.”.

Referee #4:

In budding yeast, the ribosome-associated chaperone Zuo1 is required for survival in the presence of the mTOR inhibitor rapamycin. Budding yeast displays a conserved starvation-like response to mTOR inhibition, characterized by reduced protein synthesis and elevated autophagy. This manuscript argues that yeast lacking Zuo1 die in the presence of rapamycin because they fail to reduce translation. Analysis of a specific, misfolded substrate and bulk protein ubiquitylation both indicate that zuo1Δ cells maintain high levels of misfolded proteins during rapamycin treatment, while wildtype cells clear these proteins. It is argued that the persistence of misfolded proteins arises because zuo1Δ cells continue to translate new protein under conditions where wildtype cells reduce translation. Deletion of the co-translational Ssb chaperones, which depend on Zuo1, likewise allows high translation in the presence of rapamycin. It is argued that ribosome association of Zuo1 and interactions with Ssb proteins are required for this effect based on the phenotypes of known, structure-guided separation-of-function mutations in Zuo1. After surveying many potential client proteins, it is argued that persistent translation in zuo1Δ cells reflects a failure to degrade the essential translation initiation factor eIF4G. This eIF4G degradation appears to depend on autophagy more than the proteasome, and indeed, zuo1Δ impairs rapamycin-induced autophagy.

The implication of ribosome-associated folding chaperones with translational control in proteostasis is an interesting and novel contribution. Data presented in the manuscript robustly support this basic conclusion, although the links from Zuo1 (and Ssb?), to autophagy, to eIF4G depletion are not yet clear. Greater clarity on this connection would be valuable, but may lie beyond the scope of this work. I would support publication of this manuscript providing the more specific concerns listed below are addressed.

We thank the reviewer for his/her in-depth assessment of our manuscript and for providing constructive comments and suggestions. We addressed each of them in the revised manuscript. We direct the reviewer to our responses to each individual question below.

1. In discussing the response of yeast to ER stress, it is written that "[cells treated with] tunicamycin [are] mainly relying on eIF2 phosphorylation for stress survival."

However, budding yeast does not encode a PERK orthologue and does not phosphorylate eIF2α in response to ER stress-it relies solely on the Ire1 branch of the UPR. Translational effects of ER stress may depend on ribosome ubiquitylation (DOI: 10.1038/s41598-020-76239-3), but in any case yeast do not rely on eIF2 phosphorylation for survival in tunicamycin.

This has been modified in the main text: “This may be due to TORC1 being still active at 37°C and tunicamycin mainly relying on <u>the Ire1 branch</u> for stress survival.”

2. In many blots (e.g., Figure 3A, Figure 3B, and Figure 4D) it appears that zuo1Δ cells treated with rapamycin actually show elevated translation. Is this accurate? What would be the explanation?

Yes, this is accurate. We think that puromycylated-nascent chains are being less degraded in *zuo1Δ* cells compared to WT cells which contribute to this effect. In agreement with that, we have now data showing that puromycylated-nascent chains are indeed less efficiently degraded in *zuo1Δ* cells compared to WT cells (Figure S1B and C). This has been added in the new version of the manuscript: "To monitor overall protein degradation, we performed a pulse-chase experiment using puromycin. The pulse-chase of puromycin generates a pool of puromycylated proteins that can be subsequently assessed for degradation over time. Similar to Δss-CPY*GFP, the clearance of puromycylated proteins was compromised in *zuo1Δ* cells upon rapamycin treatment (Figure S1B and C). Together, this indicates that *zuo1Δ* cells are unable to properly adapt their proteostasis network in response to TORC1 inhibition by rapamycin.".

3. As a related point, ssb1/2Δ in Figure 4D, appears to show consistently higher puromycylation, relative to wildtype, and similar to zuo1Δ in rapamycin. Does this reflect an even more direct or constitutive role for Ssb in promoting autophagy?

This is an interesting point raised by the reviewer and we have now tested autophagy induction upon rapamycin treatment in *ssb1/2Δ* cells. We found that *ssb1/2Δ* cells have a profound defect in autophagy which could indeed contribute to higher puromycylation levels. This has been added to the new version of the manuscript (Figure S5).

4. Cycloheximide treatment clearly reduces levels of ubiquitylated proteins in zuo1Δ but levels remain higher than those in wildtype cells treated with cycloheximide. This effect is even stronger in ssb1/2Δ cells.

We agree with the reviewer that the rescue is not complete, as it is often the case in such experiments. This has been clearly specified in the main text: "Confirming this, cycloheximide treatment inhibited translation in both WT and *zuo1Δ* cells, even in the presence of rapamycin (Figure 3B), and partly rescued the impaired clearance of polyubiquitinated proteins observed in *zuo1Δ* cells following TORC1 inhibition (Figure 3C).". Regarding *ssb1/2Δ* cells, we said that cycloheximide improves their clearance of polyubiquitinated proteins, and we think that this is a correct statement.

5. The data from ZUO1 separation-of-function mutations that prevent ribosome binding (RR-AA) or Ssb interaction (HPD-AAA) seem over-interpreted. Notably, both individual mutations are expected to abolish the ribosome-associated function of Zuo1 and it is unclear why loss of ribosome binding and loss of Ssb interaction would synergize.

Hundley et al., reported that mutation in the J-domain of Zuo1 still retains residual function: "Strains expressing this mutant Zuo1 grew slowly and showed sensitivity to paromomycin, but were not as defective as mutants completely lacking the protein (Fig. 5A). The mutant protein was still associated with ribosomes, as was Ssz1 (Fig. 5B)." (PMID: 11929993). Similar observation has been made with the ribosome-binding deficient mutant of Zuo1 (Zuo1-RR-AA)

(PMID: 25639645). The rationale of the double mutant is that each mutation will strongly impede Zuo1 function and, together, will almost completely abrogate its activity. This has been clarified in the manuscript: “We next generated a double mutant (RR-AA/HPD-AAA; zuo1-2-mut) lacking both ribosome binding and a functional J domain, as Zuo1 with single mutation is known to keep residual function[39]. Zuo1-2mut showed similar level of expression as WT Zuo1 (Figure S2). Cells expressing Zuo1-2mut displayed greater sensitivity to rapamycin than those expressing the single mutants, more akin to deletion of the protein (Figure 5E).”.

The manuscript argues that, "It has been reported that even very low levels of Zuo1 are enough to preserve its function suggesting that single mutations may not be enough to fully abrogate Zuo1 activity". However, normal levels of non-functional protein are not the same as low levels of fully functional protein.

This sentence has been removed to prevent any confusion.

6. As a related point, the levels of Zuo1 mutant proteins should be assessed to ensure that their abundance is roughly equivalent to wildtype.

This has been added in the new version of the manuscript: “All mutants were efficiently expressed in *zuo1Δ* cells with only Zuo1-RR-AA having lower level of expression, which could also account for its lower ability to rescue *zuo1Δ* cells (Figure S2).” And “Zuo1-2mut showed similar level of expression as WT Zuo1 (Figure S2).”.

7. Changes in the Zuo1 interactome after rapamycin treatment may be driven most strongly by changes in the overall translatome: Aro10 and Cps1 are certainly induced under starvation conditions and it seems likely that Cpa2 and many of the other metabolic enzymes identified in the screen are likewise changing in expression. This limitation needs to be discussed when interpreting results from Figure 6.

We agree with the point raised by the reviewer, and this has been addressed in the manuscript. We have now clearly stated this limitation: “An increase of Zuo1 interaction upon rapamycin treatment can also be due to an increase of Zuo1 interactor levels. Therefore, we have listed Zuo1 interactors significantly changing by more than 1.5-fold upon rapamycin treatment using published quantitative proteomic datasets (Figure S3C)[43]. Hits with level unaffected by rapamycin are more likely to have their interaction with Zuo1 modulated by rapamycin.”.

8. In arguing against translational effects on eIF4G2 synthesis, it is argued that:
"In both untreated and rapamycin-treated conditions, a comparable proportion of eIF4G2 mRNA was found to be associated with polysomes in zuo1Δ and WT cells, indicating that eIF4G is not translated to a greater extent in the absence of Zuo1 (Figure 8B). Thus, increased synthesis is not responsible for sustaining the pool of eIF4G upon rapamycin treatment in zuo1Δ cells."

However, a change in the [typical] number of ribosomes translating an mRNA can substantially change protein levels with no change in the fraction of polysome-associated mRNA.

We agree with the reviewer that a change in the fraction of polysome-associated mRNA is not always correlated with translation. To prevent any misunderstanding, we have now removed

Figure 8B from the manuscript. This will not change our conclusion, as we show that the regulation is posttranscriptional and autophagy-dependent.

9. The argument that elevated eIF4G explained the zuo1Δ phenotype would be much stronger if more specific assays, such as persistent protein synthesis (Figures 3A, 3B, 4D, 5C, 5G, 6E) or ubiquitylated protein accumulation (Figures 1C, 3C, 3E, 5B, 5F, 6D) were performed for eIF4G overexpression.

This has already been reported in the literature and we now clearly reference it in the new version of the manuscript: ". In agreement with that, it has been shown that eIF4G overexpression is preventing the decrease of translation mediated by TORC1 inhibition upon nutrient starvation[47].".

10. Could a TIF4632-GFP fusion be used to test more specifically whether eIF4G is targeted for autophagy?

This has now been tested. While the result confirms that TIF4632-GFP is targeted for autophagy (Free GFP is generated) following rapamycin treatment (see below), two main issues were observed: (1) TIF4632-GFP has higher expression in *zuo1Δ* and *ssb1/21Δ* cells compared to WT cells and (2) the GFP stabilises TIF4632 preventing efficient degradation upon rapamycin treatment. As no clear conclusion can be made, we prefer not to include the dataset in the main manuscript. The dataset will be included in the point-by-point response to reviewers.

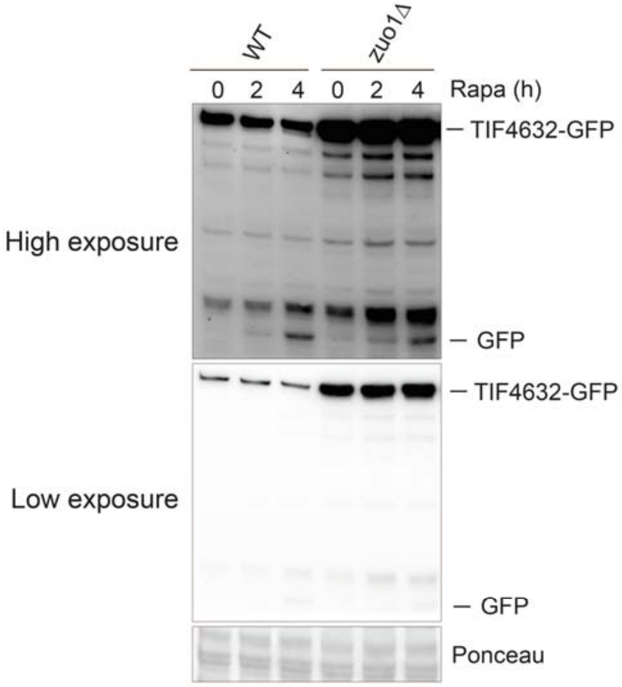

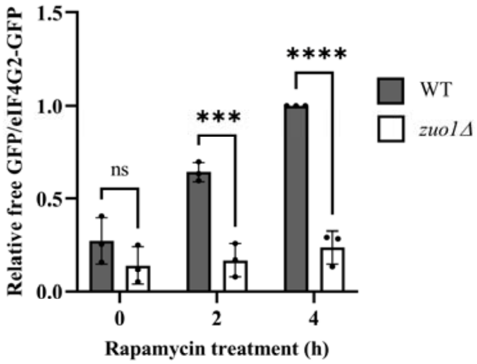

3rd Revision - Editorial Decision 15th Oct 2023

Dear Adrian,

Congratulations on a great revision! Overall, the referees have been positive and in support of publication. However there remain several editorial items that we ask you to attend to in a revised version, addressing the points below:

1. Please provide up to five keywords, which may or may not appear in the title, should be given in alphabetical order, below the abstract, each separated by a slash (/).

2. Please rename the data availability section to "Data Availability".

3. Please remove the author contribution section from the manuscript.

4. Please rename the conflict of interest section to "Disclosure and competing interests statement".

5. The references should be listed alphabetically, with up to 10 followed by et al. DOIs should be removed.

6. We require the publication of source data, particularly for electrophoretic gels and blots and graphs, with the aim of making primary data more accessible and transparent to the reader. It would be great if you could provide me with a PDF file per figure that contains the original, uncropped and unprocessed scans of all or key gels used in the figure or for graphs, an Excel spreadsheet with the original data used to generate the graphs. The PDF files should be labeled with the appropriate figure/panel number, and should have molecular weight marker; further annotation could be useful but is not essential. The PDF files will be published online with the article as supplementary "Source Data" files.

7. We include a synopsis of the paper (see http://emboj.embopress.org/). Please provide me with a general summary statement and 3-5 bullet points that capture the key findings of the paper.

8. We also need a summary figure for the synopsis. The size should be 550 wide by 200-440 high (pixels). You can also use something from the figures if that is easier.

9. Please rename the summary to "Abstract".

10. Please remove Inclusion and Diversity statement.

11. Please provide the specific URL for PXD039550 dataset in the Data Availability statement

12. Please ensure that the legends of Figure 7A-D are in alphabetical order.

13. Please include the statistical test used for data analysis in the legend of figure 8a.

14. Please add N in the legend of figure 6b.

15. Please add a scale bar and its definition for figure 8f.

Thank you for the opportunity to consider your work for publication. I look forward to your revision.

Kind regards,

Kelly

Kelly M Anderson, PhD
Editor, The EMBO Journal
k.anderson@embojournal.org

Further information is available in our Guide For Authors: https://www.embopress.org/page/journal/14602075/

authorguide

Referee #3:

I have been through the manuscript again quite carefully and the authors have addressed all of my previous comments. I believe the manuscript is much improved.

Referee #4:

Revisions have addressed my concerns and I appreciate the data added to exclude a role for eIF2α phosphorylation in the phenomenon described. I support publication in its present form.

The authors addressed the remaining editorial issues.

4th Revision - Editorial Decision 26th Oct 2023

Dear Adrien,

Congratulations on an excellent manuscript, I am pleased to inform you that your manuscript has been accepted for publication in the EMBO Journal. Thank you for your comprehensive response to the referee concerns and for providing detailed source data. It has been a pleasure to work with you to get this to the acceptance stage.

I will begin the final checks on your manuscript before submitting to the publisher next week. Once a the publisher, it will take about 3 weeks for your manuscript to be published online. As a reminder, the entire review process, including referee concerns and your point-by-point response will be available to readers.

I will be in touch throughout the final editorial process until publication. In the meantime, I hope you find time to celebrate!

Kind regards,
Kelly

Kelly M Anderson, PhD
Editor, The EMBO Journal
k.anderson@embojournal.org

Your manuscript will be processed for publication in the journal by EMBO Press. Manuscripts in the PDF and electronic editions of The EMBO Journal will be copy edited, and you will be provided with page proofs prior to publication. Please note that supplementary information is not included in the proofs.

You will be contacted by Wiley Author Services to complete licensing and payment information. The required 'Page Charges Authorization Form' is available here: https://www.embopress.org/pb-assets/embo-site/tej_apc.pdf - please download and complete the form and return to embopressproduction@wiley.com

EMBO Press participates in many Publish and Read agreements that allow authors to publish Open Access with reduced/no publication charges. Check your eligibility: https://authorservices.wiley.com/author-resources/Journal-Authors/open-access/affiliation-policies-payments/index.html

Should you be planning a Press Release on your article, please get in contact with embojournal@wiley.com as early as possible, in order to coordinate publication and release dates.

*

EMBO Press Author Checklist

Corresponding Author Name: Adrien Rousseau	
Journal Submitted to: EMBO Journal	
Manuscript Number: EMBOJ-2022-113240R1-Q	

USEFUL LINKS FOR COMPLETING THIS FORM
The EMBO Journal - Author Guidelines
EMBO Reports - Author Guidelines
Molecular Systems Biology - Author Guidelines
EMBO Molecular Medicine - Author Guidelines

Reporting Checklist for Life Science Articles (updated January
This checklist is adapted from Materials Design Analysis Reporting (MDAR) Checklist for Authors. MDAR establishes a minimum set of requirements in transparent reporting in the life sciences (see Statement of Task: 10.31222/osf.io/9sm4x). Please follow the journal's guidelines in preparing your
Please note that a copy of this checklist will be published alongside your article.

Abridged guidelines for figures
1. Data
The data shown in figures should satisfy the following conditions:
- the data were obtained and processed according to the field’s best practice and are presented to reflect the results of the experiments in an accurate and unbiased manner.
- ideally, figure panels should include only measurements that are directly comparable to each other and obtained with the same assay.
- plots include clearly labeled error bars for independent experiments and sample sizes. Unless justified, error bars should not be shown for technical
- if n<5, the individual data points from each experiment should be plotted. Any statistical test employed should be justified.
- Source Data should be included to report the data underlying figures according to the guidelines set out in the authorship guidelines on Data

2. Captions
Each figure caption should contain the following information, for each panel where they are relevant:
- a specification of the experimental system investigated (eg cell line, species name).
- the assay(s) and method(s) used to carry out the reported observations and measurements.
- an explicit mention of the biological and chemical entity(ies) that are being measured.
- an explicit mention of the biological and chemical entity(ies) that are altered/varied/perturbed in a controlled manner.
- the exact sample size (n) for each experimental group/condition, given as a number, not a range;
- a description of the sample collection allowing the reader to understand whether the samples represent technical or biological replicates (including how many animals, litters, cultures, etc.).
- a statement of how many times the experiment shown was independently replicated in the laboratory.
- definitions of statistical methods and measures:
  - common tests, such as t-test (please specify whether paired vs. unpaired), simple χ2 tests, Wilcoxon and Mann-Whitney tests, can be unambiguously identified by name only, but more complex techniques should be described in the methods section;
  - are tests one-sided or two-sided?
  - are there adjustments for multiple comparisons?
  - exact statistical test results, e.g., P values = x but not P values < x;
  - definition of ‘center values’ as median or average;
  - definition of error bars as s.d. or s.e.m.

Please complete ALL of the questions below.
Select "Not Applicable" only when the requested information is not relevant for your study.

Materials

Newly Created Materials	Information included in the manuscript?	In which section is the information available? (Reagents and Tools Table, Materials and Methods, Figures, Data Availability Section)
New materials and reagents need to be available; do any restrictions apply?	Yes	Materials and Methods

Antibodies	Information included in the manuscript?	In which section is the information available? (Reagents and Tools Table, Materials and Methods, Figures, Data Availability Section)
For **antibodies** provide the following information:<br>- Commercial antibodies: RRID (if possible) or supplier name, catalogue number and or/clone number<br>- Non-commercial: RRID or citation	Yes	Materials and Methods

DNA and RNA sequences	Information included in the manuscript?	In which section is the information available? (Reagents and Tools Table, Materials and Methods, Figures, Data Availability Section)
**Short novel DNA or RNA including primers, probes**: provide the sequences.	Not Applicable	

Cell materials	Information included in the manuscript?	In which section is the information available? (Reagents and Tools Table, Materials and Methods, Figures, Data Availability Section)
**Cell lines:** Provide species information, strain. Provide accession number in repository **OR** supplier name, catalog number, clone number, and/**OR** RRID.	Yes	Table S1
**Primary cultures:** Provide species, strain, sex of origin, genetic modification status.	Not Applicable	
Report if the cell lines were recently **authenticated** (e.g., by STR profiling) and tested for mycoplasma contamination.	Not Applicable	

Experimental animals	Information included in the manuscript?	In which section is the information available? (Reagents and Tools Table, Materials and Methods, Figures, Data Availability Section)
**Laboratory animals or Model organisms:** Provide species, strain, sex, age, genetic modification status. Provide accession number in repository **OR** supplier name, catalog number, clone number, **OR** RRID.	Not Applicable	
**Animal observed in or captured from the field:** Provide species, sex, and age where possible.	Not Applicable	
Please detail **housing and husbandry conditions**.	Not Applicable	

Plants and microbes	Information included in the manuscript?	In which section is the information available? (Reagents and Tools Table, Materials and Methods, Figures, Data Availability Section)
**Plants:** provide species and strain, ecotype and cultivar where relevant, unique accession number if available, and source (including location for collected wild specimens).	Not Applicable	
**Microbes:** provide species and strain, unique accession number if available, and source.	Not Applicable	

Human research participants	Information included in the manuscript?	In which section is the information available? (Reagents and Tools Table, Materials and Methods, Figures, Data Availability Section)
If collected and within the bounds of privacy constraints report on age, sex and gender or ethnicity for all study participants.	Not Applicable	

Core facilities	Information included in the manuscript?	In which section is the information available? (Reagents and Tools Table, Materials and Methods, Figures, Data Availability Section)
If your work benefited from core facilities, was their service mentioned in the acknowledgments section?	Yes	ACKNOWLEDGMENTS

Design

Study protocol	Information included in the manuscript?	In which section is the information available? (Reagents and Tools Table, Materials and Methods, Figures, Data Availability Section)
If study protocol has been **pre-registered, provide DOI in the manuscript**. For clinical trials, provide the trial registration number **OR** cite DOI.	Not Applicable	
Report the **clinical trial registration number** (at ClinicalTrials.gov or equivalent), where applicable.	Not Applicable	

Laboratory protocol	Information included in the manuscript?	In which section is the information available? (Reagents and Tools Table, Materials and Methods, Figures, Data Availability Section)
Provide DOI OR other citation details if **external detailed step-by-step protocols** are available.	Not Applicable	

Experimental study design and statistics	Information included in the manuscript?	In which section is the information available? (Reagents and Tools Table, Materials and Methods, Figures, Data Availability Section)
Include a statement about **sample size** estimate even if no statistical methods were used.	Yes	Figure legends
Were any steps taken to minimize the effects of subjective bias when allocating animals/samples to treatment (e.g. **randomization procedure**)? If yes, have they been described?	Yes	Figure legends
Include a statement about **blinding** even if no blinding was done.	Yes	Materials and Methods
Describe **inclusion/exclusion criteria** if samples or animals were excluded from the analysis. Were the criteria pre-established?<br><br>If sample or data points were omitted from analysis, report if this was due to attrition or intentional exclusion and provide justification.	Not Applicable	
For every figure, are **statistical tests** justified as appropriate? Do the data meet the assumptions of the tests (e.g., normal distribution)? Describe any methods used to assess it. Is there an estimate of variation within each group of data? Is the variance similar between the groups that are being statistically compared?	Yes	Figure legends

Sample definition and in-laboratory replication	Information included in the manuscript?	In which section is the information available? (Reagents and Tools Table, Materials and Methods, Figures, Data Availability Section)
In the figure legends: state number of times the experiment was **replicated** in laboratory.	Yes	Figure legends
In the figure legends: define whether data describe **technical or biological replicates**.	Yes	Figure legends

Ethics

Ethics	Information included in the manuscript?	In which section is the information available? (Reagents and Tools Table, Materials and Methods, Figures, Data Availability Section)
Studies involving **human participants**: State details of **authority granting ethics approval** (IRB or equivalent committee(s), provide reference number for approval.	Not Applicable	
Studies involving **human participants**: Include a statement confirming that **informed consent** was obtained from all subjects and that the experiments conformed to the principles set out in the WMA Declaration of Helsinki and the Department of Health and Human Services Belmont Report.	Not Applicable	
Studies involving **human participants:** For publication of **patient photos**, include a statement confirming that consent to publish was obtained.	Not Applicable	
Studies involving experimental **animals**: State details of **authority granting ethics approval** (IRB or equivalent committee(s), provide reference number for approval. Include a statement of compliance with ethical regulations.	Not Applicable	
Studies involving **specimen and field samples:** State if relevant **permits** obtained, provide details of authority approving study; if none were required, explain why.	Not Applicable	

Dual Use Research of Concern (DURC)	Information included in the manuscript?	In which section is the information available? (Reagents and Tools Table, Materials and Methods, Figures, Data Availability Section)
Could your study fall under dual use research restrictions? Please check biosecurity documents and list of **select agents and toxins** (CDC): https://www.selectagents.gov/sat/list.htm	Not Applicable	
If you used a select agent, is the security level of the lab appropriate and reported in the manuscript?	Not Applicable	
If a study is subject to dual use research of concern regulations, is the name of the **authority granting approval and reference number** for the regulatory approval provided in the manuscript?	Not Applicable	

Reporting

The MDAR framework recommends adoption of discipline-specific guidelines, established and endorsed through community initiatives. Journals have their own policy about requiring specific guidelines and recommendations to complement MDAR.

Adherence to community standards	Information included in the manuscript?	In which section is the information available? (Reagents and Tools Table, Materials and Methods, Figures, Data Availability Section)
State if relevant guidelines or checklists (e.g., **ICMJE, MIBBI, ARRIVE, PRISMA**) have been followed or provided.	Not Applicable	
For **tumor marker prognostic studies,** we recommend that you follow the **REMARK** reporting guidelines (see link list at top right). See author guidelines, under 'Reporting Guidelines'. Please confirm you have followed these guidelines.	Not Applicable	
For **phase II and III randomized controlled trials**, please refer to the **CONSORT** flow diagram (see link list at top right) and submit the CONSORT checklist (see link list at top right) with your submission. See author guidelines, under 'Reporting Guidelines'. Please confirm you have submitted this list.	Not Applicable	

Data Availability

Data availability	Information included in the manuscript?	In which section is the information available? (Reagents and Tools Table, Materials and Methods, Figures, Data Availability Section)
Have **primary datasets** been deposited according to the journal's guidelines (see 'Data Deposition' section) and the respective accession numbers provided in the Data Availability Section?	Yes	Data availability
Were **human clinical and genomic datasets** deposited in a public access-controlled repository in accordance to ethical obligations to the patients and to the applicable consent agreement?	Not Applicable	
Are **computational models** that are central and integral to a study available without restrictions in a machine-readable form? Were the relevant accession numbers or links provided?	Not Applicable	
If publicly available data were reused, provide the respective **data citations in the reference list**.	Yes	Figure legends
