# Supplementary material for: The ribosome‐associated chaperone Zuo1 controls translation upon TORC1 inhibition
Source: EMBO J. 2023 Nov 20;42(24):e113240. doi: 10.15252/embj.2022113240 (PMC10711665; doi:10.15252/embj.2022113240)
Supplement: Supplementary file 4 — Source Data for Expanded View [file EMBJ-42-e113240-s006.zip › EMBOJ-2022-113240R3-Figure_Source_Data_EV2-sd.zip!/Figure EV2/Raw - Figure EV2.pdf]

Figure EV2C

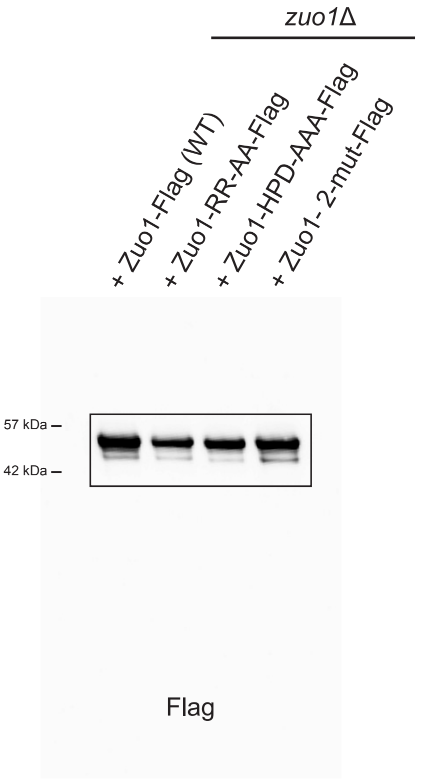

zuo1Δ
+ Zuo1-Flag (WT)
+ Zuo1-RR-AA-Flag
+ Zuo1-HPD-AAA-Flag
+ Zuo1- 2-mut-Flag
57 kDa
42 kDa
Flag

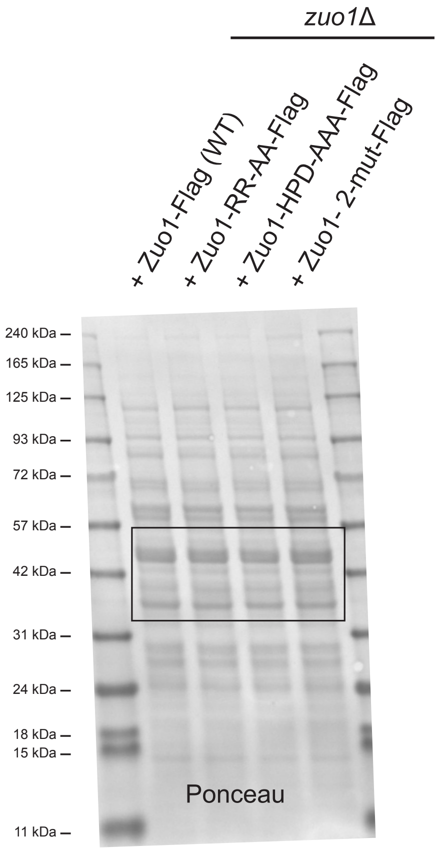

zuo1Δ
+ Zuo1-Flag (WT)
+ Zuo1-RR-AA-Flag
+ Zuo1-HPD-AAA-Flag
+ Zuo1- 2-mut-Flag
240 kDa
165 kDa
125 kDa
93 kDa
72 kDa
57 kDa
42 kDa
31 kDa
24 kDa
18 kDa
15 kDa
11 kDa
Ponceau
