# Supplementary material for: The ribosome‐associated chaperone Zuo1 controls translation upon TORC1 inhibition
Source: EMBO J. 2023 Nov 20;42(24):e113240. doi: 10.15252/embj.2022113240 (PMC10711665; doi:10.15252/embj.2022113240)

Figure EV4A

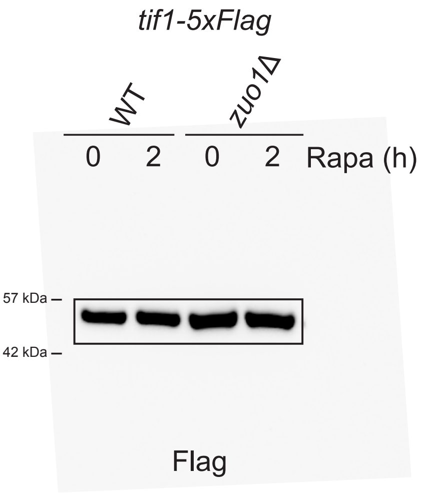

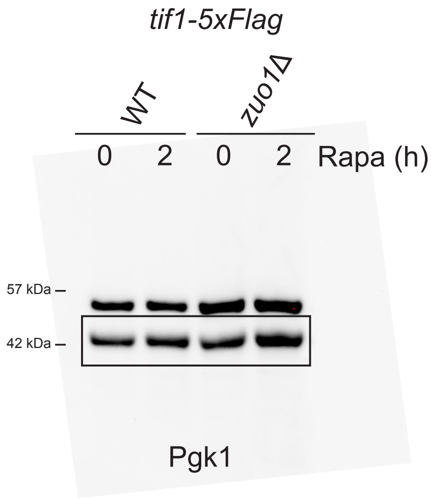

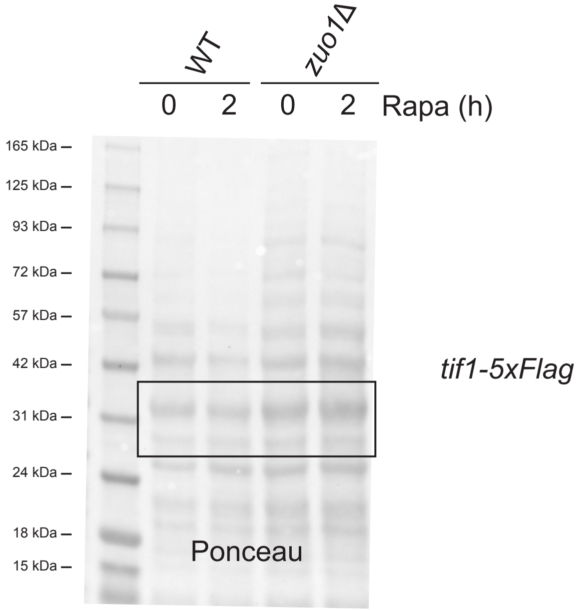

Figure S4B

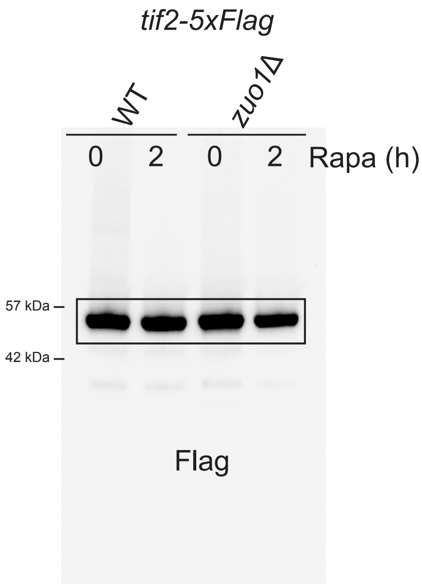

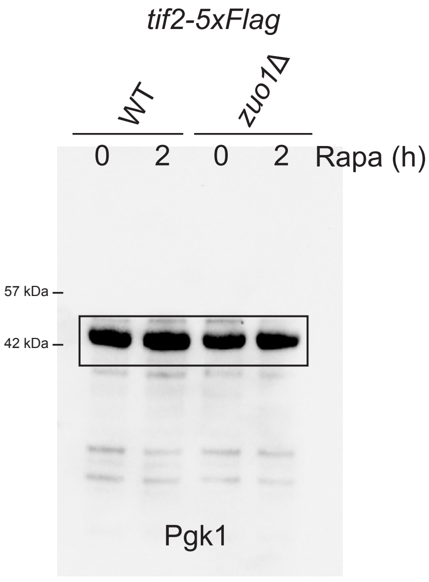

Figure S4B

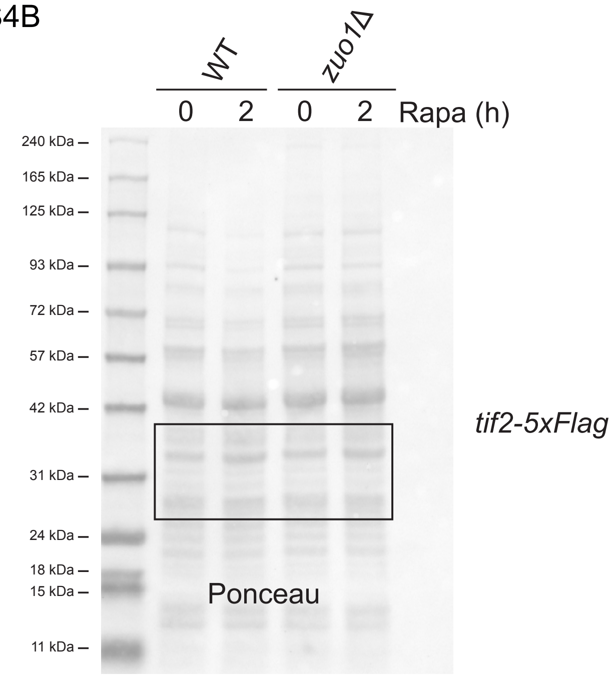

Figure S4C

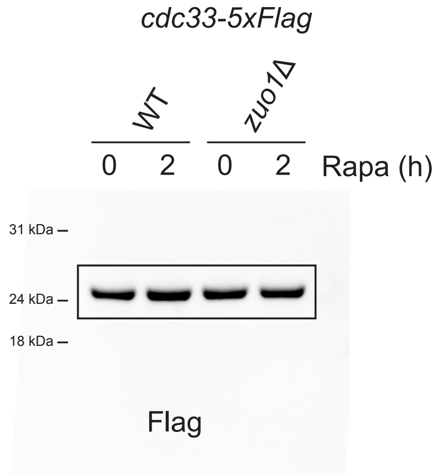

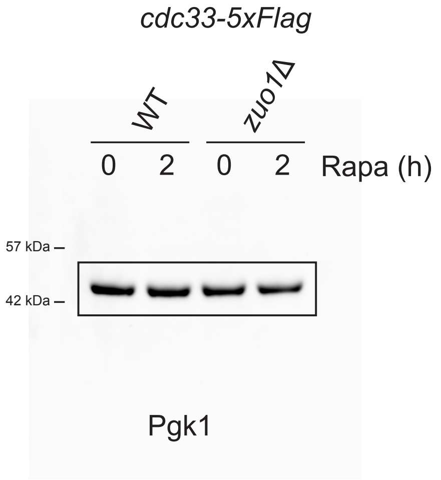

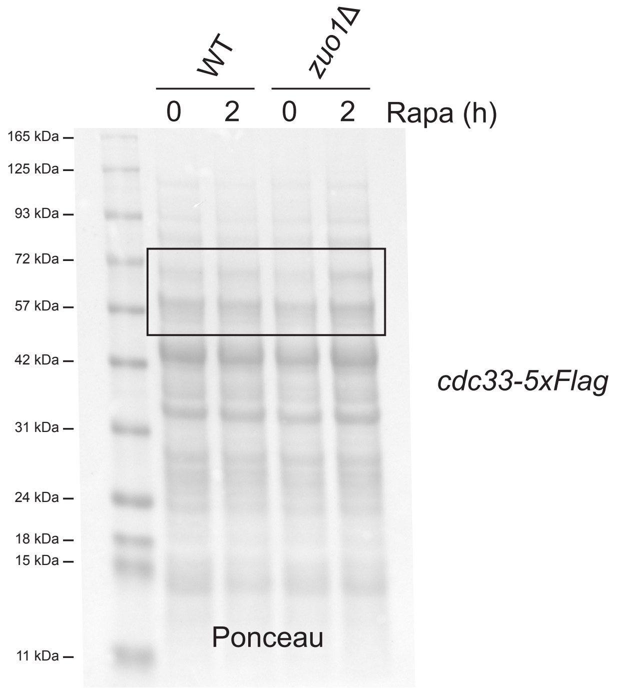

Supplement: Supplementary file 4 — Source Data for Expanded View [file EMBJ-42-e113240-s006.zip › EMBOJ-2022-113240R3-Figure_Source_Data_EV4-sd.zip!/Figure EV4/Raw - Figure EV4.pdf]
